# Evaluation of *Staphylococcus aureus* Lipoproteins: Role in Nutritional Acquisition and Pathogenicity

**DOI:** 10.3389/fmicb.2016.01404

**Published:** 2016-09-13

**Authors:** Shideh V. Shahmirzadi, Minh-Thu Nguyen, Friedrich Götz

**Affiliations:** Microbial Genetics, Interfaculty Institute of Microbiology and Infection Medicine, University of TübingenTübingen, Germany

**Keywords:** *Staphylococcus*, *S. aureus* USA300, lipoprotein, lipoprotein functions, lipoprotein dissemination, ion transporters, pathogenicity

## Abstract

Bacterial lipoproteins (Lpp) represent a major class of membrane proteins. They are distinguished by a lipid moiety at the N-terminus by which they are anchored either in the outer leaflet of the cytoplasmic membrane or, in Gram-negative bacteria, also in the inner leaflet of the outer membrane. In Gram-positive bacteria Lpp significantly contribute to nutrient transport, Toll-like receptor 2 activation and pathogenicity. Here we examine the Lpp of *Staphylococcus aureus* USA300, as a prototype for a multiple antibiotic resistant and community-acquired pathogen that is rapidly spreading worldwide. The compiled Lpp were grouped according to the postulated function and dissemination of homologs in the genus *Staphylococcus* and beyond. Based on this evaluation we also point out Lpp as promising vaccine candidates.

## Introduction

Bacterial lipoproteins (Lpp) are a distinctive class of membrane-anchored proteins. They contain a N-terminal lipid modification, the *N*-acyl-*S*-diacyl-glyceryl-cysteine (Hantke and Braun, 1973). There are three cytoplasmic membrane localized enzymes involved in the biogenesis of Lpp: the phosphatidylglycerol-prolipoprotein diacylglyceryl transferase (Lgt) (Sankaran and Wu, 1994), the specific signal peptidase II (Lsp) that recognizes the diacylglyceryl modification and cleaves between the amino acid at position −1 and the lipid-modified cysteine residue (Hussain et al., 1982), and finally the *N*-acyltransferase (Lnt) to form *N*-acyl diacylglyceryl cysteine (Gan et al., 1995). This maturation of Lpp is uniform in Gram-negative and—positive bacteria. In Gram-positive bacteria Lpp are anchored in the outer leaflet of the cytoplasmic membrane and may extend into the cell wall and beyond. Although their anchoring at the cell envelope is completely different from the covalently cell wall anchored proteins mediated by the sortase (Mazmanian et al., 2001), there is an overlap which have both in common, namely, the binding to external components, such as nutrients and host proteins. Particularly this function is affected when the maturation of pre-Lpp by the Lgt and Lsp does not take place.

The crucial role of maturation of pre-Lpp by the Lgt and Lsp for virulence and TLR2 signaling has been reviewed recently (Nguyen and Götz, 2016). For example the Δ*lgt* mutants of various *S. aureus* strains were severely affected in immune stimulation and pathogenicity (Stoll et al., 2005; Schmaler et al., 2009); and the Δ*lgt* mutants were also affected in iron acquisition under infectious conditions (Schmaler et al., 2009, 2010). In many other Gram-positive bacteria like *Mycobacterium tuberculosis* (Sander et al., 2004), *Streptococcus pneumoniae* (Petit et al., 2001; Khandavilli et al., 2008), *Streptococcus agalactiae* (Henneke et al., 2008; Bray et al., 2009), *Streptococcus pyogenes*, and *Streptococcus equi* (Sutcliffe and Harrington, 2002; Hamilton et al., 2006; Weston et al., 2009; Sutcliffe et al., 2012) or *Listeria monocytogenes* (Baumgärtner et al., 2007; Machata et al., 2008) deletion of the *lgt* or the *lsp* gene likewise impaired growth and pathogenicity.

By screening the *S. aureus* N315 genome with the new Lpp search program ParSeq (Schmollinger et al., 2004), more than 70 putative Lpp were identified, but only 55 contained a signal peptide with the right length (Stoll et al., 2005). 35 of the Lpp could be annotated as transporters for iron, zinc, amino acid, oligopeptide, glycine betaine, sugar, and teichoic acid, other had enzymatic functions such heme/copper-type cytochrome/quinol oxidase, protein-disulfide isomerase, peptidyl-prolyl *cis/trans* isomerase (PrsA), or pyruvate-format-lyase-activating enzyme. In this report it has also been shown that SitC was one of the most abundant Lpp and that in a *lgt* mutant only 20–25% retained in the membrane, while the majority was released into the supernatant (Stoll et al., 2005). In the very detailed review by Sibbald et al. 43 core and a similar amount of variant Lpp were analyzed and they also mention that the translocation pathway is mostly Sec- but in some cases also Tat-mediated (Sibbald et al., 2006).

In the meantime the knowledge as to the function of Lpp in *S. aureus* has increased, justifying a reevaluation of the data. We carefully analyzed the Lpp in strain USA300 as an epidemic prototype and traced the distribution of each Lpp homolog in the *S. aureus* species, the *Staphylococcus* genus and beyond. This allows us to unravel housekeeping and virulence associated Lpp. It turned out that particularly pathogenic strains have a number of additional Lpp serving as transporters for nutrients and contributing to virulence and fitness.

## Materials and methods

### Bioinformatic study

We collected all the predicted Lpp generated by different programs, for example Hidden Markov Model (Bagos et al., 2008), LipoP (Juncker et al., 2003), G+LPP (Sutcliffe and Harrington, 2002), G+LPPv2 (Rahman et al., 2008), LIPPREP (Taylor et al., 2006), Dolop (Babu et al., 2006), and Von Heijne (von Heijne, 1989). These lipoproteins were screened carefully by combination of the different values including hydrophobic plot (http://gcat.davidson.edu/DGPB/kd/kyte-doolittle.htm), lipobox sequence (http://www.mrc-lmb.cam.ac.uk/genomes/dolop/lipobox.shtml) and the cleaving site by PREP-LIPO (http://bioinformatics.biol.uoa.gr/PRED-LIPO/) or LipoP (http://www.cbs.dtu.dk/services/LipoP/). All Lpp sequences were blasted with other staphylococcal species and other genera by following programs NCBI protein blast (http://blast.ncbi.nlm.nih.gov/Blast.cgi?PAGE=Proteins) or KEGG (http://www.genome.jp/kegg/ko.html).

### Results and discussion

#### Evaluation of lipoproteins (Lpp) in *Staphylococcus aureus* USA300

Here, we re-evaluated the Lpp of a pathogenic *S. aureus* strain and categorized the Lpp according to the function and dissemination. Methicillin-resistant *S. aureus* USA300 was chosen as a model strain as it is a major source of community-acquired infections almost worldwide (Diep et al., 2006). The common Lpp prediction programs yielded different numbers of proposed Lpp in the 2560 genes of USA300 (http://biophysics.biol.uoa.gr/PRED-LIPO-results/): DOLOP (**52**), G+LPP (**54**), von heijne (**66**), PS51257 (**68**), PS00013 (**65**) and LipoP (**68**) and PRED-LIPO (**63**). Because of this heterogeneity each proposed Lpp was examined for the presence of an unambiguous Lpp signal peptide (LSP), which must fulfill three criteria: length between 16 and 40 amino acids, presence of a hydrophobic domain, followed by the lipobox. Based on these criteria we propose 67 Lpp in USA300, which is 2.57% of all genes (Table 1). Three of the identified Lpp in USA300 (no **5**, **65**, and **64** of Table 1) were incorrectly annotated by using the wrong start codon; these Lpp were also recently detected in the supernatant of *S. aureus* Newman by proteomic analysis (Vu et al., 2016). The size of the Lpp ranged from 6 to 89 kDa, however, the average size was between 30 and 50 kDa.

**Table 1:** **Lpp of ***S. aureus*** USA300**.

**No**	**Locus tag**	**Function/Annotation**	**PFAM**	**SP (aa)**	**Lipobox**	**Mass (KDa)**	**Dissemination^a^**	**References**
		**Fe transport**						
01	SAUSA300_1978	Ferric hydroxamate receptor/FhuD1	Peripla_BP_2	17	LTA **C**	34	15^*^	Sebulsky and Heinrichs, 2001; Sebulsky et al., 2004
02	SAUSA300_2235	Fe ABC transporter/FhuD2	Peripla_BP_2, ABC2_membrane_3	17	LAA **C**	34	16	Sebulsky and Heinrichs, 2001; Sebulsky et al., 2004; Mariotti et al., 2013
03	SAUSA300_0721	Transferrin receptor/SstD	Peripla_BP_2	18	LAA **C**	38	14	Morrissey et al., 2000
04	SAUSA300_0117	Fe ABC transporter/SirA	Peripla_BP_2	20	LAG **C**	37	10	Heinrichs et al., 1999
05	SAUSA300_1032	Fe ABC transporter/IsdE	Peripla_BP_2	19	LTS **C**	32	10	Mazmanian et al., 2002, 2003; Grigg et al., 2007
06	SAUSA300_0344	FepA, Fe-binding protein, part of fepABC and tat-AC cluster	Peptidase_M75	17	IAA **C**	32	10	Biswas et al., 2009
07	SAUSA300_2136	Fe ABC transporter	Peripla_BP_2	21	VAA **C**	36	14	
08	SAUSA300_0219	Iron Binding Protein	SBP_bac_1, 6, 8, 11	17	LSA **C**	36	4^*^	
		**Other cation transport**						
09	SAUSA300_0618	Manganese-binding protein MntC (SitC)	ZnuA, Nit_Regul_Hom	17	VAA **C**	34	19	Cockayne et al., 1998; Müller et al., 2010; Diep et al., 2014
10	SAUSA300_2351	Zinc-binding, adcA-like	ZnuA, ZinT	20	LAA **C**	57	10	
11	SAUSA300_2411	Cobalt and nickel transporter Cnt (Opp1A)	SBP_bac_5	20	LTG **C**	59	10	Remy et al., 2013
12	SAUSA300_0231	Nickel ABC transporter	SBP_bac_5	18	LSG **C**	55	10^*^	
13	SAUSA300_0203	Nickel-Peptide/transporter substrate-binding protein	SBP_bac_5	18	LSG **C**	66	+	
14	SAUSA300_2230	Molybdenum ABC transporter (ModA)	SBP_bac_11, 1, PBP_like_2	19	LAG **C**	29	15	Neubauer et al., 1999
		**Anion transport**						
15	SAUSA300_1283	Phosphate ABC transporter	PBP_like_2, PBP_like	20	LGA **C**	36	15	
16	SAUSA300_0145	Phosphonate ABC transporter	Phosphonate-bd, SBP_bac_3	20	AAA **C**	35	10	
17	SAUSA300_0175	Nitrate ABC transporter substrate-binding protein	NMT1_2,	17	ITG **C**	36	4	
		**AA and Peptide transport**						
18	SAUSA300_2391	Glycine betaine /carnitine/ choline ABC transporter (OpuCc)	OpuAC	20	LSG **C**	37	19	
19	SAUSA300_2359	Amino acid ABC transporter	SBP_bac_3	17	LAA **C**	13	12	
20	SAUSA300_0073	Peptide ABC transporter	SBP_bac_5	19	LAG **C**	57	11	
21	SAUSA300_0891	Oligopeptide ABC transporter (Opp3A)	SBP_bac_5	20	LSG **C**	61	11	Hiron et al., 2007
22	SAUSA300_0892	Oligopeptide ABC transporter (Opp4A)	SBP_bac_5	20	LSA **C**	63	5	Hiron et al., 2007
23	SAUSA300_0437	NLPA/ D-Methionine binding (GmpC)	Lipoprotein_9 (NLPA)	17	LAA **C**	31	9	Williams et al., 2004
24	SAUSA300_0798	D-Methionine ABC transporter	OpuAC, Lipoprotein_9	19	LAA **C**	30	15	
		**Sugar transport**						
25	SAUSA300_0209	Maltose ABC transporter	SBP_bac_1, 8	20	VTA **C**	47	6	
		**Miscellaneous functions**						
		**Biosynthesis**						
26	SAUSA300_1884	CamS sex pheromone biosynthesis	CamS	17	LAA **C**	44	14	
		**Respiration**						
27	SAUSA300_0963	Quinol oxidase, subunit II (QoxA)	COX2	19	LSG **C**	41	21	
28	SAUSA300_0693	Electron transfer domain/SaeP	CfAFP, DM13	20	LGA **C**	16	22	Makgotlho et al., 2013
		**Chaperone-Foldases**						
29	SAUSA300_1790	Foldase protein PrsA	Rotamase,	20	LGA **C**	36	15	Heikkinen et al., 2009; Jousselin et al., 2012
30	SAUSA300_2354	Thioredoxin/Protein disulfide-isomerase	Thioredoxin_2, 4, 5	18	LTA **C**	22	15^*^	
		**Protein translocation**						
31	SAUSA300_2046	YidC (OxaA)–essential protein	OATP, 60KD_IMP	19	LAG **C**	32	25	
		**Phage and plasmid encoded Lpp**						
32	SAUSA300_1436	PhiSLT ORF144-like	DUF1510, Zip, Presenilin	17	LTA **C**	16	2^*^	
33	pUSA300_HOUMR0011	Membrane bound penicillinase BlaZ		16	LSA **C**	31	11	Nielsen and Lampen, 1982b
		**Lpl cluster**						
34	SAUSA300_0410	Lpl-1 νSaα specific	DUF576	32	IAG **C**	30	+	Nguyen et al., 2015b
35	SAUSA300_0411	Lpl-2 νSaα specific	DUF576	22	IIG **C**	30	+	Nguyen et al., 2015b
36	SAUSA300_0413	Lpl-3 νSaα specific	DUF576	23	IIG **C**	30	+	Nguyen et al., 2015b
37	SAUSA300_0414	Lpl-4 νSaα specific	DUF576	22	VTS **C**	28	+	Nguyen et al., 2015b
38	SAUSA300_0415	Lpl-5 νSaα specific	DUF576	22	IMG **C**	29	+	Nguyen et al., 2015b
39	SAUSA300_0416	Lpl-6 νSaα specific	DUF576	20	MAG **C**	29	+	Nguyen et al., 2015b
40	SAUSA300_0417	Lpl-7 νSaα specific	DUF576	23	IVG **C**	30	+	Nguyen et al., 2015b
41	SAUSA300_0418	Lpl-8 νSaα specific	DUF576	22	VTS **C**	29	+	Nguyen et al., 2015b
42	SAUSA300_0419	Lpl-9 νSaα specific	DUF576	22	IGG **C**	30	+	Nguyen et al., 2015b
43	SAUSA300_2429	Tandem lpp	DUF576	22	IGG **C**	16	3^*^	
44	SAUSA300_2430	Tandem lpp	DUF576	23	IGA **C**	29	+	
45	SAUSA300_0100	Tandem lpp/Conserved staphylococcal antigen 1A (Csa1A)	DUF576	24	TAG **C**	28	+	Schluepen et al., 2013
46	SAUSA300_0101	Tandem lpp	DUF576	24	TAG **C**	28	+	
47	SAUSA300_0102	Tandem lpp	DUF576	24	TAG **C**	28	+	
48	SAUSA300_0103	Tandem lpp	DUF576	23	TAG **C**	28	+	
		**Unknown function**						
49	SAUSA300_0079	Unknown function	DUF1541	18	LSA **C**	20	17	
50	SAUSA300_0372	Unknown function	PepSY	18	LTA **C**	21	17^*^	
51	SAUSA300_0377	Unknown function	DUF1748	19	LTG **C**	23	14	
52	SAUSA300_1492	Unknown function		16	LAG **C**	13	15^*^	
53	SAUSA300_0992	Cell-wall binding lipoprotein	YkyA, EzrA	19	LAG **C**	23	13^*^	
54	SAUSA300_2403	Unknown function	DUF1307	20	LSA **C**	17	12^*^	
55	SAUSA300_0724	Unknown function	IncA, TarH	19	ISA **C**	32	12	
56	SAUSA300_2315	Unknown function	PA26, IncA, CLN3	17	LAA **C**	23	11^*^	
57	SAUSA300_2614	Unknown function	DUF_1980	20	LYS **C**	42	6	
58	SAUSA300_0663	Unknown function	PA26, IncA	17	LTG **C**	15	5	
59	SAUSA300_1106	Unknown function	FAM176	18	VAG **C**	35	5^*^	
60	SAUSA300_0303	Unknown function	DUF4467	17	LAG **C**	14	5^*^	
61	SAUSA300_1478	Unknown function	DUF4467	17	LSA **C**	13	3^*^	
62	SAUSA300_1376	Unknown function	DUF1672	17	LSG **C**	34	2^*^	
63	SAUSA300_1379	Unknown function	DUF1672	17	LSG **C**	34	2^*^	
64	SAUSA300_1440	Unknown function	DUF1672	17	LGG **C**	34	2^*^	
65	SAUSA300_1742	Unknown function		18	LTA **C**	23	2^*^	
66	SAUSA300_1741	Unknown function	ETRAMP, Myco_19_kDa	18	LTA **C**	6	+	
67	SAUSA300_0769	Unknown function	DUF5067	17	LGA **C**	27	+	

a *The number indicates the number of staphylococcal species in which the corresponding homologues gene/protein (more than 40% identity over the whole protein length) is present*.

*+S. aureus specific gene*.

* *Staphylococcal specific gene*.

*The corresponding gene has been compared in 34 staphylococcal species and other genera in which genome sequence has been available. Abbreviations: 60KD_IMP, 60Kd inner membrane protein; ABC2_membrane_3, ABC-2 family transporter protein; CamS, CamS sex pheromone cAM373 precursor; CfAFP, Choristoneura fumiferana antifreeze protein (CfAFP); CLN3, CLN3 protein; DM13, Electron transfer DM13; COX2, Cytochrome C oxidase subunit II; DUF, Domain Unknown Function; ETRAMP, Malarial early transcribed membrane protein (ETRAMP); EzrA, Septation ring formation regulator, IncA, IncA protein; Myco_19_kDa, Mycobacterium 19 kDa lipoprotein antigen; NMT1_2, NMT1-like family; Nit_Regul_Hom, Uncharacterized protein, homolog of nitrogen regulatory protein PII; OATP, Organic Anion Transporter Polypeptide (OATP) family; OpuAC, Substrate binding domain of ABC-type glycine betaine transport system; PA26, PA26 p53-induced protein (sestrin); PBP_like_2, PBP superfamily domain; PepSY, Peptidase propeptide and YPEB domain; Peripla_BP_2, Periplasmic binding protein; Pfam, Protein Families; Phosphonate-bd, ABC transporter, phosphonate, periplasmic substrate-binding protein; Rotamase, PPIC-type PPIASE domain; SBP_bac, Bacterial extracellular solute-binding protein; SP, Signal Peptide; TarH, Tar ligand binding domain homolog; YkyA, Putative cell-wall binding lipoprotein; ZnuA, Zinc-uptake complex component A periplasmic; ZinT, (YodA) periplasmic lipocalin-like zinc-recruitment; Zip, ZIP Zinc transporter*.

#### Functional categorization of Lpp

We grouped the Lpp according to their function. Most of the functions were deduced from studies of homologous counterparts in other bacteria, but in 12 cases the ascribed functions were confirmed by molecular/biochemical studies in *S. aureus*.

##### Iron transporters

The first group in Table 1 represent Lpp that are involved in iron acquisition which is extremely important in order for pathogenic bacteria to obtain some of the limited free iron ions during infection. For this reason intricate iron transport and iron regulatory systems have evolved in pathogenic bacteria to guarantee sufficient iron supply (Braun, 2001). It is therefore not surprising that 8 Lpp are involved iron acquisition or utilization of host-derived heme iron (*isd* operon) as an iron source. Under iron limitation, iron(III)-hydroxamate siderophores are excreted as iron chelators by the FhuCBG system (Sebulsky et al., 2000) and the iron-loaded chelators are bound by two Lpp (FhuD1 and FhuD2) acting as receptors and representing the first step in iron acquisition. Although both receptors are homologous (41% identity) they exhibit different activities. FhuD2 is conserved in other species and binds a broad spectrum of Fe chelators, such as ferric hydroxamate, and various siderophores, such as ferrichrome, ferrioxamineB, aerobactin, and coporgen. FhuD1, on the other hand, is only found in staphylococcal species and binds only ferrichrome and ferrioxamine B, and exhibits a lower affinity for hydroxamate siderophore binding than FhuD2 (Sebulsky and Heinrichs, 2001; Sebulsky et al., 2004). The Lpp SirA belongs to the iron regulated SirABC operon (Heinrichs et al., 1999). Mutants of either *sirA* or *sirB* are unable to take up iron complexes, such as ferric hydroxamates, ferric enterobactin or ferric citrate but they retain the ability to produce staphylobactin (Dale et al., 2004). During infection and iron limitation *S. aureus* is also able to use heme as an iron source via the complex *isdCDEFsrtBisdG* operon. IsdA and IsdB are involved in heme uptake, which is derived from the host hemoglobin. IsdC is a cell wall localized transporter, while IsdD, IsdE, and IsdF are the membrane translocation factors, and IsdG, is the cytoplasmic heme-iron binding protein (Mazmanian et al., 2003). While IsdA, IsdB, and IsdC are covalently cell-wall bound by sortase anchoring, IsdE is the heme-binding Lpp (Mazmanian et al., 2002; Grigg et al., 2007). Another iron-limited expressed siderophore transport system is composed by 4 proteins SstA,B,C, and D where SstD is the Lpp (Morrissey et al., 2000). The twin-arginine translocation (Tat) pathway, is present in only some staphylococcal species, and is composed of TatA and TatC (Biswas et al., 2009). The *tat*AC operon is associated with the *fep*ABC operon. FepA is a Lpp and mediates iron binding, FepB with its typical TAT signal peptide is the iron-dependent peroxidase (FepB), and FepC is supposed to be the high affinity iron permease (Biswas et al., 2009). The advantage of the *fep*-*tat* cluster could lie in iron uptake and in the external detoxification of reactive oxygens. Indeed, the iron uptake under iron limitation was significantly decreased in the *fep*-*tat* mutants, which were less virulent in a mouse kidney abscess model (Biswas et al., 2009). The last two Lpp in this group (**7** and **8**) were annotated as iron binding Lpp, however, their function is unknown. The USA300_2136 (**7**) is encoded in the operon with two other genes, both were annotated as ion ABC transporter permease proteins containing the same motifs as FecCD and ABC3. The USA300_0219 (**8**) contains a SBP_bac motif (Table 1).

##### Other cation and anion transporters

Beside iron transporters there are other cation transporters for Co, Cu, Mn, Mo, Ni, Zn ions, which become important when these trace elements are limited. In some cases, they are crucial in infection. One of the most abundant Lpp in *S. aureus* is the 33 kDa MntC (SitC) (Stoll et al., 2005). Originally it was referred to as SitC because its protein sequence shares 77% identity to SitC of *S. epidermidis*, where it has been described as being involved in iron transport (Cockayne et al., 1998). However, instead of being involved in iron transport, SitC has recently been found to have a role in manganese (Mn) transport and is part of the MntABC system (Horsburgh et al., 2002; Diep et al., 2014). To avoid confusion we use here the term MntC (SitC). Three Lpp were annotated as Ni transporters. The Opp1 transport system is involved in cobalt and nickel uptake and has been renamed as Cnt (Remy et al., 2013). There is a Cnt related protein (25% identity) encoded by SAUSA300_0203 that was annotated as peptide/nickel transport system. The Lpp, ModA, is part of the molybdate transporter complex (ModABC) (Neubauer et al., 1999).

Based on annotation the following Lpp are involved in phosphate (USA300_1283), phosphonate (USA300_0145) and nitrate transport (USA300_0175). They are encoded with other genes in the same operon involved in the transport apparatus.

##### Amino acid and peptide transporters

There are 7 Lpp involved in amino acid and peptide transport. Opp3 is a main system that provides oligopeptides as a nutritional source (Hiron et al., 2007), and GmpC binds the dipeptide glycyl-methionine and is part of an ABC transporter system (Williams et al., 2004).

##### Sugar transport

There is only one Lpp involved in sugar transport, the maltose ABC transporter.

##### Lpp with miscellaneous functions

The group of miscellaneous Lpp comprise Lpp with diverse functions. They are involved in sex pheromone biosynthesis, in terminal electron transfer to oxygen (QoxA) or in global regulation (SaeP). QoxA is part of the terminal cytochrome *aa3* quinol oxidase encoded by *qoxABCD* (Götz and Mayer, 2013; Hammer et al., 2013). The protein complex SaePQ activates the phosphatase activity of sensor kinase SaeS in the SaeRS two-component system of *S. aureus* (Jeong et al., 2012). The Sae system controls the expression of numerous virulence factors, such as the extracellular adherence protein (Eap), which facilitates host cell invasion (Makgotlho et al., 2013). The chaperon, PrsA, belongs to the parvulin PPIase family (pepdidyl-prolyl cis/trans isomerase) that assists posttranslocational folding at the outer surface of the cytoplasmic membrane (Heikkinen et al., 2009). In *Bacillus*, PrsA is an essential protein. In *S. aur*eus it is not essential, but a *prsA* deletion mutant is impaired in post-transcriptional maturation of PBP2A and shows therefore decreased methicillin resistance (Jousselin et al., 2012, 2015). There is another chaperone annotated as a thioredoxin/protein disulfide-isomerase related to DsbA that catalyzes disulfide formation and isomerization and that acts simultaneously as a chaperone by preventing protein aggregation (Kouwen et al., 2007). YidC (short form of OxaI-like protein) acts as a membrane integrase for Sec-dependent substrates, such as ATP synthase subunit a (Foa) or cytochrome *bo*_3_ oxidase CyoA subunit, but can also act as a chaperone and as an assembly site for membrane protein folding (Wang and Dalbey, 2011). YidC is highly conserved in bacteria and appears to be essential; at least we were unable to delete the gene in *S. aureus*. There are also pro-phage and plasmid encoded Lpp. The function of the prophage encoded PhiSLT is unknown. However, the plasmid encoded Lpp in USA300 encodes for a beta-lactamase (BlaZ), which was one of the first Lpp discovered in *S. aureus* (Nielsen and Lampen, 1982a). This gene is plasmid encoded in many other *S. aureus* strains such as JH1, JH9, N315, but it may also be chromosomally located as in MRSA252 or Bmb9393. The advantage of the membrane-anchored penicillinase is that it is retained at the cell surface and is not diluted in the environment like the secreted ones.

##### Paralogous tandem lpp gene cluster

USA300 belongs to the hypervirulent clonal complex CC8 (Robinson et al., 2005; Cockfield et al., 2007), Most of these virulent strains carry a conserved genomic island termed νSaα that encodes a number of homologous *lpp* arranged in tandem, referred to as “lipoprotein-like” (*lpl*) (Babu et al., 2006). Most likely, the *lpl* cluster represents an example of paralogous genes in *S. aureus*, which are homologs genes in this species and that have diverged after a duplication event. USA300 carries nine such *lpl* genes. The exact activity of the Lpl proteins is unknown. However, recently it has been shown that this *lpl* cluster triggers host cell invasion, increases pathogenicity, and it has been speculated that the *lpl* cluster contributes to epidemic of the CC8 and CC5 strains (Nguyen et al., 2015a). Moreover, there are two other clusters of paralogous *lpp* genes that also contain the same conserved motifs as DUF567 of unknown function, and which are taxonomically restricted to staphylococci. With one of the paralogous Lpp Csa1A the structure has been determined that revealed a new structure family (Schluepen et al., 2013).

##### Lpp with unknown function

**Figure 1:**
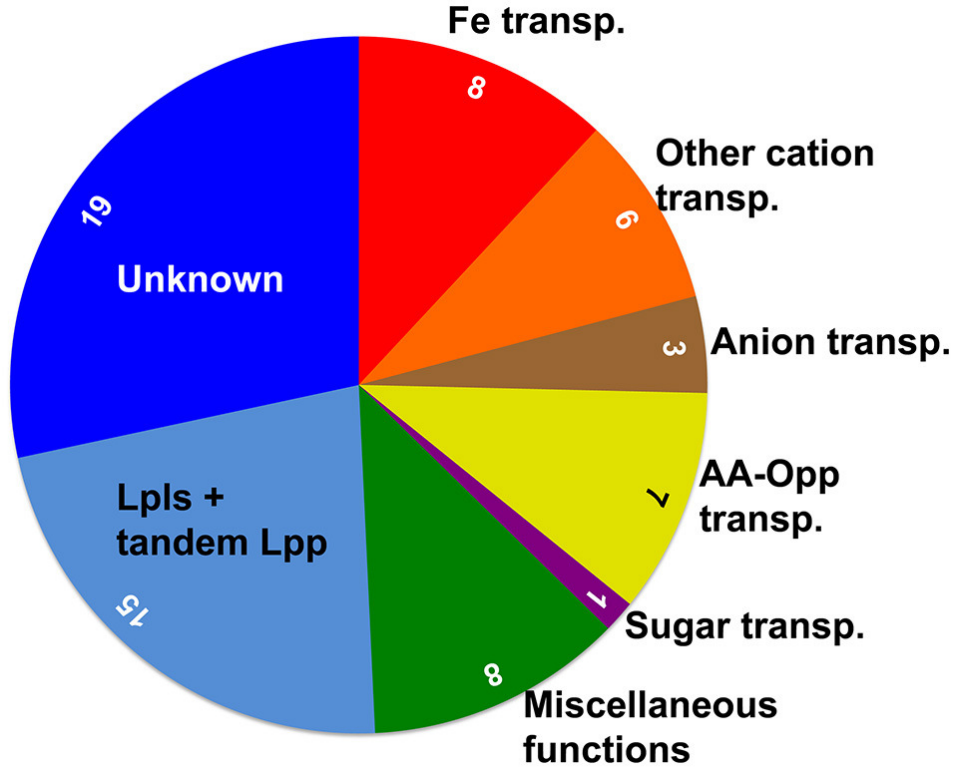
**Schematic representation of the functional distribution of the 67 Lpp in ***S. aureus*** USA300**. The area of the sectors, with the inserted number of Lpp, indicates the proportional distribution. Alone 25 (36%) of the Lpp are involved in ion and nutrient transport, and the ’miscellaneous’ group contains important enzymes and chaperones. USA300 carries relatively high number of 15 (22%) tandem Lpp, to which also the 9 Lpl proteins belong that are encoded by the νSaα genomic island and which play a role in pathogenicity; however, their exact function is still unknown. Finally, the function of 28% of the Lpp is completely unknown.

Among the 67 Lpp there are 19 (28%) with unknown function. A graphical representation of the grouped Lpp is shown in Figure 1.

#### Dissemination of Lpp in the genus *Staphylococcus* and beyond

**Figure 2:**
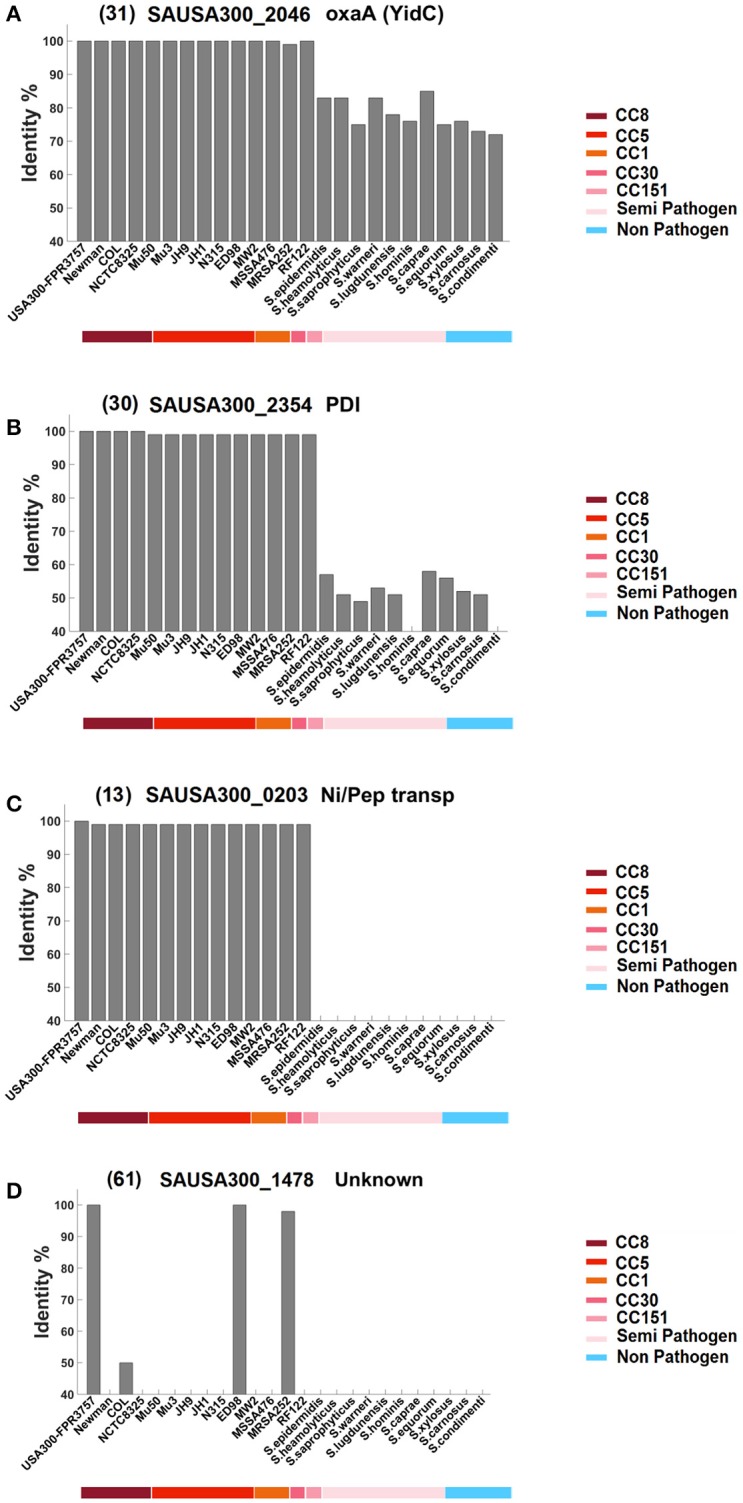
**Classification of the Lpp into four groups based on their similarity and dissemination in bacteria**. Each Lpp was blasted against the indicated *S. aureus* strains and other staphylococcal species representatives. The cut off was ≥40% identity over the entire protein sequence. The colored bar below the listed strains indicates clonal complexes (CC-types) of *S. aureus* (first half), as well as other staphylococcal species representatives, grouped in semi-pathogenic and non-pathogenic (second half). Group **(A)** represents Lpp that are highly conserved in the *Staphylococcus* genus but also in many other genera; an example for this group is YidC, an essential protein in many bacteria. Group **(B)** represents Lpp that are mainly found in the genus *Staphylococcus*; the example for this group is PDI, a proposed thioredoxin disulfide-isomerase. Group **(C)** represents Lpp mainly found in the *S. aureus* species; an example is the proposed nickel-peptide transporter. Group **(D)** represents strain-specific Lpp essentially occurring in the strain USA300; the example is an unknown Lpp. The number in front of the gene ID refers to the corresponding numbering in Table 1.

We questioned how widely distributed the various Lpp are. Do they only occur in single strains, the entire species of *S. aureus*, the whole genus, or even beyond? Therefore, the amino acid sequence of each Lpp was analyzed by BLAST to identify corresponding homologs. As a cut off we have chosen ≥40% identity over the entire protein sequence. The comparison was restricted to those species and their representatives where a genome was available. With regards to their abundance we categorized the Lpp of USA300 into four groups: Group A comprises Lpp that were widely distributed in the entire *Staphylococcus* genus and in many other genera. Group B comprises *Staphylococcus* genus specific Lpp. Group C represent Lpp that are mainly found only in *S. aureus*, and Group D represents only a few Lpp that are essentially USA300 specific. How these 4 groups differ from each other is illustrated in Figure 2, which shows one example of each Lpp group.

The first half of the strains represent *S. aureus* strains; the underlined colored bar indicates their association to the corresponding clonal complexes (CC-types). The second halve of the strains represent a selection of other staphylococcal species; the underlined colored bar indicates whether they are regarded as semi-pathogenic or non-pathogenic. In the A and B group the USA300 Lpp are highly conserved in the *S. aureus* species (>95 identity) thus comprising a homogeneous block. Members of the A group are distinguished by a broad dissemination and are highly conserved in other staphylococcal species (>70% identity) and in many other genera (≥40% identity). The group B Lpp are less conserved in other staphylococcal species and are only occasionally found in other genera. Group C lists Lpp that essentially occur in the species *S. aureus* apart from a few examples that show a slightly broader dissemination. And, finally, group D lists Lpp that are essentially strain specific for USA300 and that are not found in other *S. aureus* strains apart from few exceptions. A complete listing of all 67 Lpp is shown in Supplementary Figure 1.

**Group A** Lpp comprise highly conserved Lpp with a broad dissemination (Table 2A). Most likely they play an important role in metabolism or are involved in basic cellular processes. The majority of these Lpp are annotated as transporters. The corresponding homologous proteins are found in many, but not all, other staphylococcal species, and they are also found in many other genera. Examples of the most conserved Lpp that were even found in completely unrelated genera such Gram-negatives or high-GC Gram-positives are: Fe transporter-SirA (**04**), IsdE (**05**), FepA (**06**), MntC (SitC) (**09**), Phosphate ABC transporter (**15**), Glycine betaine transporter (**18**), Amino acid ABC transporter (**19**), D-Methionine transporter (**24**), Quinol oxidase (QoxA) (**27**), Electron transfer domain/SaeP (**28**), YidC (**31**), BlaZ (**33**), or the Lpp with unknown function (**49**). The latter Lpp is unusual in so far as it is only found in USA300 but not in other *S. aureus* strains; however, it is present in many other staphylococcal species and even in other genera. The most disseminated Lpp is the D-Methionine ABC transporter (**24**). In *E. coli* the MetNIQ transporter, belonging to ABC type permease superfamily, is involved in the uptake of both D- and L-methionine. MetN is the putative ATPase, MetI is a membrane spanning protein and MetQ (the Lpp) is the major binding protein for both L- and D-methionine as well as their analogs, such as N-formyl methionine (Merlin et al., 2002). In complex medium the transporter is not essential for growth, which also applies for most other Lpp. However, under certain nutrient limitations and environmental conditions they are crucial for growth and survival. So far we know only one Lpp that is essential even in complex medium, and that is YidC (**31**). YidC is evolutionarily conserved and is involved in membrane biogenesis in bacteria, mitochondria, and chloroplasts. It comprises several activities, such as acting as a protein insertase, as chaperone, and as an assembly factor for transmembrane proteins (Wang and Dalbey, 2011).

**Table 2A:** **USA300 Lpp with broad dissemination**.

**No^1^**	**Function/annotation**	**Staphylococcal species and other genera^2^**
02	Fe ABC transporter/FhuD2	*S.aureus, S.capitis, S.carnosus, S.equorum, S.gallinarum, S.haemolyticus, S.hyicus, S.lugdunensis, S.pasteuri, S.pettenkoferi, S.pseudintermedius, S.saprophyticus, S.schleiferi, S.simiae, S.warneri*,
		*S.xylosus*,
		***Bacillus, Exiguobacterium, Macrococcus, Salinicoccus***
04	Fe ABC transporter/SirA	*S.aureus, S.agnetis, S.arlettae, S.delphini, S.equorum, S.hyicus, S.intermedius, S.lugdunensis, S.pseudintermedius, S.schleiferi*
		***Advenella, Bacillus, Chromohalobacter, Clostridium, Gynuella, Hahella, Halobacteroides, Halomonas, Haloplasma, Jeotgalibacillus, Lysinibacillus, Marinomonas, Paenibacillus, Pectobacterium, Planococcus, Sporosarcina***
05	Fe ABC transporter/IsdE	*S.aureus, S.auricularis, S.capitis, S.caprae, S.condimenti, S.lentus, S.lugdunensis, S.pasteuri, S.sciuri, S.simulans*
		***Bacillus, Carnobacterium, Coprococcus, Eubacterium, Lactobacillus, Listeria, Lysinibacillus, Paenibacillus, Solibacillus, Streptococcus, Terribacillus***
06	FepA, Fe-binding protein, part of fep ABC and tat-AC cluster	*S.aureus, S.capitis, S.carnosus, S.condimenti, S.epidermidis, S.haemolyticus, S.lugdunensis, S.pasteuri, S.simiae, S.warneri*
		***Actinobacillus, Actinoplanes, Bacillus, Bibersteinia, Brevibacillus, Clavibacter, Corynebacterium, Dermacoccus, Exiguobacterium, Gallibacterium, Hyphomicrobium, Kineococcus, Kitasatospora, Kyrpidia, Leptotrichia, Listeria, Lysinibacillus, Mannheimia, Moraxella, Mycobacterium, Neisseria, Paenibacillus, Planococcus, Pseudomonas, Streptococcus, Streptomyces, Yersinia***
09	Manganese-binding protein MntC (SitC)	*S.aureus, S.capitis, S.caprae, S.carnosus, S.condimenti, S.epidermidis, S.equorum, S.haemolyticus, S.hominis, S.hyicus, S.lugdunensis, S.pasteuri, S.pseudintermedius, S.saprophyticus, S.schleiferi, S.simiae, S.simulans, S.warneri, S.xylosus*
		***Aerococcus, Amphibacillus, Bacillus, Carnobacterium, Clostridium, Enterococcus, Exiguobacterium, Finegoldia, Lactobacillus, Macrococcus, Oceanobacillus, Paenibacillus, Streptomyces, Terribacillus, Tetragenococcus***
10	Zinc-binding, adcA-like	*S.aureus, S.capitis, S.haemolyticus, S.hyicus, S.pasteuri, S.pseudintermedius, S.saprophyticus, S.schleiferi, S.warneri, S.xylosus*
		***Bacillus, Enterococcus, Halobacillus, Streptococcus***
11	Cobalt and nickel transporter Cnt (Opp1A)	*S.aureus, S.epidermidis, S. hyicus, S.lugdunensis, S.pasteuri, S.pseudintermedius, S.saprophyticus, S.schleiferi, S.warneri, S.xylosus*,
		***Aggregatibacter, Bacillus, Brevibacillus, Eubacterium, Mannheimia, Methanosarcina, Paenibacillus, Proteus***
14	Molybdenum ABC transporter (ModA)	*S.aureus, S.capitis, S.caprae, S.carnosus, S.epidermidis, S.haemolyticus, S.hyicus, S.lugdunensis, S.pasteuri, S.pseudintermedius, S.saprophyticus, S.schleiferi, S.simiae, S.warneri, S.xylosus*
		***Bacillus, Clostridium, Macrococcus, Paenibacillus, Salinicoccus, Syntrophobotulus***
15	Phosphate ABC transporter	*S.aureus, S.capitis, S.caprae, S.carnosus, S.epidermidis, S.haemolyticus, S.hyicus, S.lugdunensis, S.pasteuri, S.pseudintermedius, S.saprophyticus, S.schleiferi, S.simiae, S.warneri, S.xylosus*
		***Alkaliphilus, Anoxybacillus, Bacillus, Clostridium, Enterobacter, Escherichia, Eubacterium, Exiguobacterium, Geobacillus, Halobacillus, Jeotgalibacillus, Lysinibacillus, Macrococcus, Maribacter, Paenibacillus, Planococcus, Pleurocapsa, Salinicoccus, Sebaldella, Streptomyces, Thermobacillus, Thermosediminibacter***
16	Phosphonate ABC transporter	*S.aureus, S.capitis, S.caprae, S.epidermidis, S.haemolyticus, S.hominis, S.lugdunensis, S.pasteuri, S.saprophyticus, S.warneri*
		***Bacillus, Carnobacterium, Enterobacter, Enterococcus, Lactobacillus, Melissococcus, Paenibacillus, Terribacillus, Weissella***
17	Nitrate ABC transporter	*S.aureus, S.pseudintermedius, S.saprophyticus, S.schleiferi*
		***Bacillus, Clostridium, Geobacillus, Methanosarcina, Ruminiclostridium, Solibacillus***
18	Glycine betaine /carnitine/choline ABC transporter (OpuCc)	*S.aureus, S.capitis, S.carnosus, S.cohnii*, *S.condimenti, S.epidermidis, S.equorum, S.gallinarum, S.haemolyticus, S.hyicus, S.lugdunensis, S.pasteuri, S.saprophyticus, S.schleiferi, S.simiae, S.simulans, S.succinus, S.warneri, S.xylosus*
		***Bacillus, Carnobacterium, Enterococcus, Lactobacillus, Listeria, Macrococcus, Pediococcus, Solibacillus, Streptococcus, Tetragenococcus, Virgibacillus***
19	Amino acid ABC transporter	*S.aureus, S.capitis, S.caprae, S.carnosus, S.epidermidis, haemolyticus, S.lugdunensis, S.pasteuri, S.saprophyticus, S.simiae, S.warneri, S. S.xylosus*
		***Aggregatibacter, Anoxybacillus, Arthrobacter, Bacillus, Brevibacillus, Brucella, Campylobacter, Clostridium, Ensifer, Gallibacterium, Geobacillus, Haemophilus, Hafnia, Halobacillus, Jeotgalibacillus, Macrococcus, Mesorhizobium, Neisseria, Neorhizobium, Nocardia, Ochrobactrum, Paenibacillus, Photorhabdus, Raoultella, Rhizobium, Sinorhizobium, Streptococcus, Xenorhabdus***
21	Oligopeptide ABC transporter (Opp3A)	*S.aureus, S.capitis, S.carnosus, S.epidermidis, S.haemolyticus, S.lugdunensis, S.pasteuri, S.pseudintermedius, S.saprophyticus, S.schleiferi, S.xylosus*
		***Anoxybacillus, Bacillus, Carnobacterium, Geobacillus, Macrococcus***
23	NLPA/D-methionine binding, (GmpC)	*S.aureus, S.epidermidis, S.simiae, S.capitis, S.lugdunensis, S.xylosus, S.carnosus, S.schleiferi, S.pseudintermedius*
		***Aerococcus, Amphibacillus, Bacillus, Carnobacterium, Clostridium, Enterococcus, Exiguobacterium, Finegoldia, Lactobacillus, Listeria, Macrococcus, Megasphaera, Oceanobacillus, Paenibacillus, Ralstonia, Streptococcus, Streptomyces, Terribacillus, Tetragenococcus***
24	D-Methionine ABC transporter	*S.aureus, S.capitis, S.caprae, S.carnosus, S.cohnii*, *S.epidermidis, S.haemolyticus, S.hyicus, S.lugdunensis, S.pasteuri, S.pseudintermedius, S.saprophyticus, S.schleiferi, S.warneri, S.xylosus*
		***Acetohalobium, Acidaminococcus, Actinoplanes, Advenella, Amphibacillus, Anoxybacillus, Arcobacter, Azospirillum, Bacillus, Bifidobacterium, Caldicellulosiruptor, Campylobacter, Carnobacterium, Cedecea, Citrobacter, Clostridium, Cronobacter, Desulfitobacterium, Desulfosporosinus, Enterobacter, Enterobacteriaceae, Enterococcus, Erwinia, Exiguobacterium, Finegoldia, Geobacillus, Halobacillus, Jeotgalibacillus, Ketogulonicigenium, Klebsiella, Kluyvera, Kosakonia, Lactobacillus, Leptotrichia, Lipotes, Listeria, Macrococcus, Megamonas, Megasphaera, Melissococcus, Paenibacillus, Pectobacterium, Pediococcus, Pelosinus, Planococcus, Pseudomonas, Salinicoccus, Sebaldella, Selenomonas, Serratia, Sodalis, Solibacillus, Streptomyces, Sulfurospirillum, Terribacillus, Thermoanaerobacterium, Veillonella, Virgibacillus, Xanthomonas***
25	Maltose ABC transporter	*S.aureus, S.delphini, S.intermedius, S.lentus, S.pseudintermedius, S.sciuri*
		***Bacillus, Geobacillus, Salinicoccus***
26	CamS sex pheromone biosynthesis	*S.aureus, S.capitis, S.carnosus, S.epidermidis, S.haemolyticus, S.hyicus, S.lugdunensis, S.pasteuri, S.pseudintermedius, S.saprophyticus, S.schleiferi, S.simiae, S.warneri, S.xylosus*
		***Escherichia, Macrococcus, Salinicoccus***
27	Quinol oxidase, subunit II (QoxA)	*S.aureus, S.agnetis, S.auricularis, S.capitis, S.caprae, S.carnosus, S.chromogenes, S.cohnii, S.epidermidis, S.haemolyticus, S.hominis, S.hyicus, S.lugdunensis, S.pasteuri, S.pettenkoferi, S.pseudintermedius, S.saprophyticus, S.schleiferi, S.simiae, S.warneri, S.xylosus*,
		***Bacillus, Geobacillus, Halobacillus, Listeria, Lysinibacillus, Paenibacillus, Salinicoccus, Streptococcus, Virgibacillus***
28	Electron transfer domain/SaeP	*S.aureus, S.auricularis, S.capitis, S.caprae, S.carnosus, S.condimenti, S.delphini, S.epidermidis, S.haemolyticus, S.intermedius*, *S.lentus, S.lugdunensis, S.microti, S.pettenkoferi, S.pseudintermedius, S.saprophyticus, S.schleiferi, S.sciuri, S.simiae, S.simulans, S.vitulinus, S.xylosus*
		***Bacillus, Brochothrix, Carnobacterium, Enterococcus, Fictibacillus, Listeria, Mycobacterium, Paenibacillus***
31	YidC (OxaA) - essential protein	*S.aureus, S.arlettae, S.capitis, S.caprae, S.carnosus, S.cohnii, S.condimenti, S.delphini, S.epidermidis, S.equorum, S.gallinarum, S.haemolyticus, S.hominis, S.hyicus, S.lugdunensis, S.massiliensis, S.pasteuri, S.pseudintermedius, S.saprophyticus, S.schleiferi, S.simiae, S.simulans, S.succinus, S.warneri, S.xylosus*
		***Bacillus, Halobacillus, Lysinibacillus, Macrococcus, Planococcus, Salinicoccus, Solibacillus, Xylanimonas***
33	Membrane bound penicillinases BlaZ	*S.aureus, S.agnetis, S.capitis, S.epidermidis, S.equorum, S.haemolyticus, S.lugdunensis, S.pseudintermedius, S.saprophyticus, S.warneri, S.xylosus*,
		***Acidaminococcus, Bacillus, Carnobacterium, Enterococcus, Escherichia, Kribbella, Macrococcus, Paenibacillus, Salinicoccus, Streptococcus, Streptomyces***
49	Unknown function	USA300, *S.arlettae, S.capitis, S.carnosus, S.cohnii, S.epidermidis, S.equorum, S.haemolyticus, S.hominis, S.lugdunensis, S.pasteuri, S.pettenkoferi, S.pseudintermedius, S.saprophyticus, S.sciuri, S.warneri, S.xylosus*
		***Bacillus, Carnobacterium, Corynebacterium, Dermacoccus, Enterococcus, Exiguobacterium, Geobacillus, Jeotgalibacillus, Kocuria, Kytococcus, Listeria, Lysinibacillus, Macrococcus, Oceanobacillus, Paenibacillus, Planococcus, Salinicoccus, Solibacillus, Streptococcus, Terribacillus, Virgibacillus*** (see Table 2D)
57	Unknown function	*S.aureus, S.agnetis, S.capitis, S.haemolyticus, S.hyicus, S.simiae*,
		***Bacillus, Clostridium, Enterococcus, Herbinix, Lactobacillus, Leuconostoc, Streptococcus***

*Numbering and proposed function are the same as listed in Table 1*.

*The other genera are in bold letters*.

**Group B** represents Lpp that are mainly found in the genus *Staphylococcus*. Only exceptionally they were also found in the related genera *Salinicoccus* and *Bacillus*, or in the unrelated genus *Xylanimonas*, a high-GC Gram-positive cocci belonging to the *Actinomycetales* (Table 2B). Only 8 of the 16 Lpp in this list have an annotated function such as the ferric hydroxamate receptor (FhuD1) (**01**), nickel- (**12**), oligopeptide (Opp4A) (**22**) transporters, as well as thioredoxin disulfide-isomerase (**30**). The functions of the other 8 Lpp are unknown.

**Table 2B:** **USA300 Lpp mainly occurring in the genus ***Staphylococcus*****.

**No^1^**	**Function/annotation**	**Staphylococcal species and other genera^2^**
01	Ferric hydroxamate receptor/FhuD1	*S.aureus, S.capitis, S.carnosus, S.equorum, S.gallinarum, S.haemolyticus, S.hyicus, S.lugdunensis, S.pasteuri, S.saprophyticus, S.schleiferi, S.sciuri, S.succinus, S.warneri, S.xylosus*
03	Transferrin receptor/SstD	*S.aureus, S.carnosus, S.condimenti, S.epidermidis, S.haemolyticus, S.hyicus, S.lugdunensis, S.pasteuri, S.saprophyticus, S.schleiferi, S.simiae, S.simulans, S.warneri, S.xylosus*,
		***Bacillus, Macrococcus***
07	Fe ABC transporter	*S.aureus, S.capitis, S.carnosus, S.epidermidis, S.haemolyticus, S.hyicus, S.lugdunensis, S.pasteuri, S.pseudintermedius, S.saprophyticus, S.schleiferi, S.simiae, S.warneri, S.xylosus **Bacillus, Paenibacillus***
12	Nickel ABC transporter	*S.aureus, S.capitis, S.carnosus, S.epidermidis, S.pasteuri, S.pseudintermedius, S.saprophyticus, S.schleiferi, S.warneri, S.xylosus*
20	Peptide ABC transporter	USA300, *S.caprae, S.chromogenes, S.delphini, S.epidermidis, S.haemolyticus, S.hominis, S.lugdunensis, S.pasteuri, S.schleiferi, S.warneri*
		***Brevibacillus, Streptococcus*** (see Table 2D)
22	OligopeptideABC transporter (Opp4A)	*S.aureus, S.carnosus, S.hyicus, S.pseudintermedius, S.schleiferi **Bacillus***
29	Foldase protein PrsA	*S.aureus, S.capitis, S.caprae, S.carnosus, S.epidermidis, S.haemolyticus, S.hyicus, S.lugdunensis, S.pasteuri, S.pseudintermedius, S.saprophyticus, S.schleiferi, S.simiae, S.warneri, S.xylosus*
		***Macrococcus, Salinicoccus***
30	Thioredoxin/Protein disulfide-isomerase	*S.aureus, S.capitis, S.caprae, S.carnosus, S.epidermidis, S.equorum, S.gallinarum, S.haemolyticus, S.hyicus, S.lugdunensis, S.pasteuri, S.saprophyticus, S.simiae, S.warneri, S.xylosus*
50	Unknown function	*S.aureus, S.carnosus, S.condimenti, S.delphini, S.equorum, S.gallinarum, S.lugdunensis, S.pasteuri, S.pettenkoferi, S.pseudintermedius, S.schleiferi, S.sciuri, S.simiae, S.simulans, S.succinus, S.warneri, S.xylosus*
51	Unknown function	*S.aureus, S.auricularis, S.capitis, S.caprae, S.carnosus, S.epidermidis, S.haemolyticus, S.hominis, S.lugdunensis, S.pasteuri, S.saprophyticus, S.simiae, S.warneri, S.xylosus*
		***Xylanimonas***
52	Unknown function	*S.aureus, S.arlettae, S.capitis, S.caprae, S.carnosus, S.cohnii, S.epidermidis, S.haemolyticus, S.lugdunensis, S.pasteuri, S.saprophyticus, S.simiae, S.succinus, S.warneri, S.xylosus*
53	Cell-wall binding lpp	*S.aureus, S.capitis, S.caprae, S.epidermidis, S.haemolyticus, S.hominis, S.lugdunensis, S.pasteuri, S.saprophyticus, S.simiae, S.succinus, S.warneri, S.xylosus*
54	Unknown function	*S.aureus, S.capitis, S.caprae, S.carnosus, S.epidermidis, S.haemolyticus, S.hominis, S.lugdunensis, S.pasteuri, S.simiae, S.simulans, S.warneri*
55	Unknown function	*S.aureus, S.capitis, S.caprae, S.carnosus, S.epidermidis, S.haemolyticus, S.lugdunensis, S.pasteuri, S.saprophyticus, S.schleiferi, S.warneri, S.xylosus*
		***Escherichia, Streptococcus***
56	Unknown function	*S.aureus, S.capitis, S.caprae, S.epidermidis, S.hyicus, S.intermedius, S.lugdunensis, S.pasteuri, S.pseudintermedius, S.simiae, S.warneri*
58	Unknown function	*S.aureus, S.epidermidis, S.haemolyticus, S.pasteuri, S.warneri*,
		***Fusarium, Nasonia***

*Numbering and proposed function are the same as listed in Table 1*.

*The other genera are in bold letters*.

**Group C** represent Lpp that are mainly found in the *S. aureus* species (Table 2C). Only two Lpp are ascribed a function, the iron binding protein (**08**) and the nickel-peptide transporter (**13**). Besides *S. aureus*, this latter Lpp is also found in various *Bacillus* genera, suggesting that the corresponding gene was exchanged by horizontal transfer and was maintained in *S. aureus* probably because of its beneficial effect in infection. Interestingly, all 9 Lpl proteins encoded on the νSaα island, as well as two other tandem Lpp clusters, were only found in *S. aureus*. These genes likely arose by gene duplication, an important mechanism for acquiring new genes and creating genetic novelty in organisms (Magadum et al., 2013). It is striking why only *S. aureus* accumulated and preserved these Lpl and tandem Lpp. Their contribution in virulence has been shown for the Lpl proteins; they modulate the innate immunity, and enhance host cell invasion and pathogenicity (Nguyen et al., 2015a). Finding out their precise function warrants further investigation.

**Table 2C:** **USA300 Lpp essentially occurring in the ***S. aureus*** species**.

**No^1^**	**Function/annotation**	***S. aureus* and few other species/genera^2^**
08	Iron Binding Protein	*S.aureus, S.epidermidis, S.pseudintermedius, S.schleiferi*
13	Nickel-Peptide/transporter	*S. aureus* only - but also in:
		***Amphibacillus, Bacillus, Lysinibacillus, Solibacillus***
34	Lpl-1 νSaα specific	*S.aureus*
35	Lpl-2 νSaα specific	*S.aureus*
36	Lpl-3 νSaα specific	*S.aureus*
37	Lpl-4 νSaα specific	*S.aureus*
38	Lpl-5 νSaα specific	*S.aureus*
39	Lpl-6 νSaα specific	*S.aureus*
40	Lpl-7 νSaα specific	*S.aureus*
41	Lpl-8 νSaα specific	*S.aureus*
42	Lpl-9 νSaα specific	*S.aureus*
43	Tandem lpp	*S.aureus, S.heamolyticus, S.lugdunensis*
44	Tandem lpp	*S.aureus*
45	Tandem lpp (Csa1A)	*S.aureus*
46	Tandem lpp	*S.aureus*
47	Tandem lpp	*S.aureus*
48	Tandem lpp	*S.aureus*
59	Unknown function	*S.aureus, S.epidermidis, S.lugdunensis S.pasteuri, S.warneri*
60	Unknown function	*S.aureus, S.caprae, S.epidermidis, S.lugdunensis, S.warneri*
62	Unknown function	*S.aureus, S.epidermidis*
63	Unknown function	*S.aureus, S.epidermidis*
64	Unknown function	*S.aureus, S.epidermidis*
65	Unknown function	*S.aureus, S.epidermidis*
66	Unknown function	*S.aureus*
		***Bacillus***
67	Unknown function	*S.aureus*

*Numbering and proposed function are the same as listed in Table 1*.

*The other genera are in bold letters*.

**Group D** represents Lpp that are essentially USA300 specific and are not found in other *S. aureus* strains, but some are found in few other staphylococcal species or even in other genera as the Lpp (**49**) (Table 2D). The prophage encoded PhiSLT (**32**) is only found in USA300 and a limited number of other *S. aureus* strains as well as in *S. xylosus*, suggesting a limited distribution of this prophage in the genus.

**Table 2D:** **USA300 Lpp essentially occurring in strain USA300**.

20	Peptide ABC transporter	Only USA300 not in other *S. aureus* strains, but other species and genera (see Table 2B)
32	PhiSLT ORF144-like	Only USA300, ED98, MW2, MSSA476, MRSA252 and *S.xylosus*
49	Unknown function	Only USA300 not in other *S. aureus* strains, but other species and genera (see Table 2A)
61	Unknown function	Only USA300, COL, ED98, MRSA252

#### Correlation between the number of Lpp and pathogenicity

We compared the total number of Lpp (based on PREP-LIPO method) in various *S. aureus* strains belonging to different clonal complex groups (CC 8, 5, 1, 30, 151) and in representatives of other staphylococcal species (Table 3). As can be seen, the highly epidemic categorized strains (*S. aureus* USA300, Newman, Mu50, Mu3, JH1, JH9, N315) contain more than 60 putative Lpp, whereas the moderate epidemic strains (*S. aureus* NCTC8325, *S. epiderminis* RP62A, ATCC_12228, *S. haemolyticus* JCSC1435*, S. saprophyticus* ATCC 15305) contain only around 50 putative Lpp. Although speculative, this suggests that epidemic/pathogenic strains are distinguished by a higher number of Lpp.

**Table 3:** **The number of Lpp in different staphylococcal strains and species**.

**Species**		**Strains**	**Number of Lpp**
*S.aureus*	CC8	USA300	67
		Newman	64
		COL	61
		NCTC8325	50
	CC5	Mu50	66
		Mu3	66
		JH1	65
		JH9	65
		N315	64
	CC1	MSSA476	62
		MW2	60
	CC30	MRSA252	57
	CC151	RF122	56
*S. epidermidis*		RP62A	53
		ATCC_12228	48
*S. haemolyticus*		JCSC1435	51
*S. saprophyticus*		ATCC 15305	42
*S. carnosus*		TM300	58

#### Lpp as TLR2 agonists

It is long known that bacterial Lpp are recognized by Toll-like receptor 2 (TLR2) of the innate immune system and are sensed at very low concentrations (Zähringer et al., 2008). Dependent on the degree of acylation Lpp are recognized by different TLR2 heterodimers: Diacylated Lpp are recognized by TLR2 and TLR6 (Bulut et al., 2001; Takeuchi et al., 2001), while triacylated Lpp by TLR2 and TLR1 heterodimers (Takeda et al., 2002; Takeuchi et al., 2002). In Gram-negative bacteria Lpp biogenesis consists of three successive reactions catalyzed by prolipoprotein diacylglyceryl transferase (Lgt), signal peptidase II (Lsp), and apolipoprotein N-acyltransferase (Lnt) (Sankaran and Wu, 1994; Buddelmeijer, 2015). A Lnt homolog has so far not been found in staphylococci. However, Kurokawa and colleagues showed that MntC (SitC) is triacylated in exponential growth phase but becomes diacylated (lacking the alpha-aminoacylation) in post-exponential phase (Kurokawa et al., 2009, 2012). The occurrence of triacylated MntC (SitC) indicates that *S. aureus* has a apolipoprotein N-acyltransferase, which adds a fatty acid to the amino group of *S*-(diacylglyceryl) cysteine residue. MntC (SitC) was also the first native Lpp for which its TLR2 activation and its co-localization with TLR2 have been demonstrated (Kurokawa et al., 2009; Müller et al., 2010). The structure of the lipid moiety of Lpp has an enormous influence on the immune response. For example it has been shown that di- but not tri-acylated Lpp suppress immune responses and might play a role in immune tolerance (Skabytska et al., 2014).

#### Lpp as vaccine candidates

**Figure 3:**
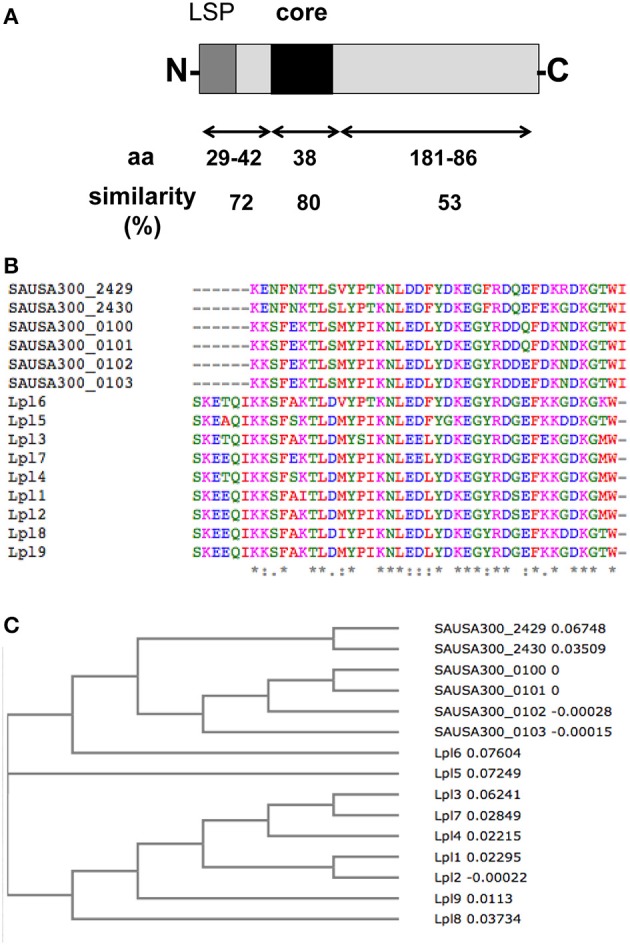
**Conserved sequence motif in Lpl and the tandem Lpp. (A)** Alignment of the 9 Lpl and the 4 tandem Lpp revealed a core sequence over 38 amino acids with 80% similarity. **(B)** Sequence alignment of the core region of 15 tandem Lpp by using Clustal Omega program. **(C)** Phylogenetic tree of the core region of 15 tandem Lpp by using Clustal Omega program.

In principle all Lpp listed in Table 1 are potential vaccine candidates, however, some are more or less suitable. All Lpp listed in Table 2C are promising vaccine candidates because they occur essentially in the *S. aureus* species. Promising vaccine candidates would be the 9 Lpl proteins (**34**–**42** in Table 1) because they share a highly conserved (92% similarity) core region of 44 amino acids (Nguyen et al., 2015a). A vaccine against this core domain would simultaneously cross-react with several antigens. There is also a set of 4 tandem Lpp (**45**–**48** in Table 1) that show a very high similarity (>80%) over the entire protein length; most likely these tandem Lpp arose by gene duplication. However, both these tandem Lpp and Lpl share also a conserved 38 amino acid long domain Figure 3. Therefore, one can expect that antibodies against this conserved domain would cross-react with 13 Lpp. The advantage of these tandem Lpp is also that they are highly present only in *S. aureus*. A vaccine would therefore not target staphylococcal skin residents such as *S. epidermidis* and other species. Another parameter for a promising Lpp-antigen is its size. The size should be large enough that the protein part can penetrate the cell wall to be accessible for antibodies. The size of the Lpp varies enormously, ranging from 6 to 89 kDa (Table 1). All Lpp with a mass >40 kDa are also good candidates like the zinc-binding (**10**), cobalt and nickel transporter Cnt (**11**), nickel ABC transporter (**12**), nickel-Peptide/ transporter (**13**), peptide ABC transporter (**20**), oligopeptide ABC transporter (Opp3A) (**21**), CamS sex pheromone biosynthesis (**26**), quinol oxidase, subunit II (QoxA) (**27**).

A third parameter for a promising vaccine candidate is the abundance of an antigen. The most abundant Lpp is MntC (SitC), which is essential for MRSA virulence during murine systemic infection (Kehl-Fie et al., 2013). It was therefore concluded that MntABC might be a potential vaccine candidate (Diep et al., 2014). Another potential vaccine candidate is the FhuD2 (**02**) involved in ferric-hydroxamate uptake. FhuD2 binds ferrichrome with nanomolar affinity and the structure of FhuD2-ferrichrome has been determined (Mariotti et al., 2013; Podkowa et al., 2014). Immunization with FhuD2 alone or together with hydroxamate siderophores was protective in a murine staphylococcal infection model (Mariotti et al., 2013). However, a break-through was reported recently with a combination of five antigens, including FhuD2 (**02**), and Csa1A (**45**), formulated with a novel adjuvant containing a TLR7-dependent agonist adsorbed to alum. This vaccine provided close to 100% protection against four different staphylococcal strains (Bagnoli et al., 2015). As can seen, certain Lpp have been already turned out experimentally as promising vaccine candidates.

## Conclusion

The re-evaluation of the chromosomal encoded Lpp in *S. aureus* USA300 was necessary, as none of the Lpp prediction tools of the public domain yielded the complete inventory of Lpp. The 67 identified Lpp constituted a solid basis for a systematic analysis. A large proportion of the Lpp is involved in the uptake of essential ions and nutrients. Frequently they function as receptors for the target molecule and are part of ABC transporter complexes. Generally one can say that 39% of the Lpp are involved in ion and nutrient transport, indicating, that this is one of their major physiological task. However, two Lpp in the miscellaneous group should be mentioned, QoxA and YidC, which play important functions in respiration and folding of membrane proteins.

Regarding the dissemination of Lpp within the *S. aureus* species and in other species we can group them into four categories: (i) those that are highly conserved and broadly disseminated, (ii) that are mainly found in the genus *Staphylococcus*, (iii) that are mainly found in the species *S. aureus*, and (iv) a few Lpp are rather strain specific. Particularly the group of *S. aureus* specific Lpp are promising vaccine candidates for future work. Our data show that the relatively high number of *lpp* genes (>60) in the *S. aureus* species is mainly due to the tandem *lpp*. There was only one *S. aur*eus strain, NCTC8325, that shows only 50 *lpp*. As this strain is used for almost 50 years as a lab strain, it is conceivable that under non-selective conditions a reversal of the *lpp* amplification occurred. Indeed, this strain contains only three tandem *lpl* genes in the νSaα island, which might represent an example of adaptive evolution. There are many open questions regarding the Lpp in *S. aureus* and also the Lpp of the staphylococcal skin microbiota that need to be studied in the future.

## Author contributions

FG designed the work, SS and MN analyzed and interpreted data, SS and MN prepared the draft of the Manuscript (MS), figures and tables; and FG wrote the MS.

## Funding

This work was supported by grants from the Deutsche Forschungsgemeinschaft (DFG; GO 371/9-1, SFB766 and TRR34) and Open Access Publishing Fund of Tuebingen University.

### Conflict of interest statement

The authors declare that the research was conducted in the absence of any commercial or financial relationships that could be construed as a potential conflict of interest.

## Abbreviations

Lpp: Lipoproteins
*lgt*: diacylglyceryl transferase enzyme encoding gene
Lpl: lipoprotein-like
*S*: *Staphylococcus*
TLR: Toll-like receptor
CC: clonal complex
AA: Amino acid Numbers behind Lpp in parenthesis refer to the numbers in Table 1.

## References

[B1] Babu M. M. Priya M. L. Selvan A. T. Madera M. Gough J. Aravind L.. (2006). A database of bacterial lipoproteins (DOLOP) with functional assignments to predicted lipoproteins. J. Bacteriol. 188, 2761–2773. 10.1128/Jb.188.8.2761-2773.2006 16585737 PMC1446993

[B2] Bagnoli F. Fontana M. R. Soldaini E. Mishra R. P. Fiaschi L. Cartocci E.. (2015). Vaccine composition formulated with a novel TLR7-dependent adjuvant induces high and broad protection against *Staphylococcus aureus*. Proc. Natl. Acad. Sci. U.S.A. 112, 3680–3685. 10.1073/pnas.1424924112 25775551 PMC4378396

[B3] Bagos P. G. Tsirigos K. D. Liakopoulos T. D. Hamodrakas S. J. (2008). Prediction of lipoprotein signal peptides in Gram-positive bacteria with a Hidden Markov Model. J. Proteome Res. 7, 5082–5093. 10.1021/pr800162c 19367716

[B4] Baumgärtner M. Kärst U. Gerstel B. Loessner M. Wehland J. Jänsch L. (2007). Inactivation of Lgt allows systematic characterization of lipoproteins from *Listeria monocytogenes*. J. Bacteriol. 189, 313–324. 10.1128/jb.00976-06 17041050 PMC1797373

[B5] Biswas L. Biswas R. Nerz C. Ohlsen K. Schlag M. Schäfer T.. (2009). Role of the twin-arginine translocation pathway in *Staphylococcus*. J. Bacteriol. 191, 5921–5929. 10.1128/jb.00642-09 19633084 PMC2747896

[B6] Braun V. (2001). Iron uptake mechanisms and their regulation in pathogenic bacteria. Int. J. Med. Microbiol. 291, 67–79. 10.1078/1438-4221-00103 11437341

[B7] Bray B. A. Sutcliffe I. C. Harrington D. J. (2009). Impact of lgt mutation on lipoprotein biosynthesis and *in vitro* phenotypes of *Streptococcus agalactiae*. Microbiology 155(Pt 5), 1451–1458. 10.1099/mic.0.025213-0 19383708

[B8] Buddelmeijer N. (2015). The molecular mechanism of bacterial lipoprotein modification–how, when and why? FEMS Microbiol. Rev. 39, 246–261. 10.1093/femsre/fuu006 25670733

[B9] Bulut Y. Faure E. Thomas L. Equils O. Arditi M. (2001). Cooperation of Toll-like receptor 2 and 6 for cellular activation by soluble tuberculosis factor and *Borrelia burgdorferi* outer surface protein A lipoprotein: role of Toll-interacting protein and IL-1 receptor signaling molecules in Toll-like receptor 2 signaling. J. Immunol. 167, 987–994. 10.4049/jimmunol.167.2.987 11441107

[B10] Cockayne A. Hill P. J. Powell N. B. Bishop K. Sims C. Williams P. (1998). Molecular cloning of a 32-kilodalton lipoprotein component of a novel iron-regulated *Staphylococcus epidermidis* ABC transporter. Infect. Immun. 66, 3767–3774. 9673260 10.1128/iai.66.8.3767-3774.1998 PMC108413

[B11] Cockfield J. D. Pathak S. Edgeworth J. D. Lindsay J. A. (2007). Rapid determination of hospital-acquired meticillin-resistant *Staphylococcus aureus* lineages. J. Med. Microbiol. 56(Pt 5), 614–619. 10.1099/jmm.0.47074-0 17446283

[B12] Dale S. E. Sebulsky M. T. Heinrichs D. E. (2004). Involvement of SirABC in iron-siderophore import in *Staphylococcus aureus*. J. Bacteriol. 186, 8356–8362. 10.1128/jb.186.24.8356-8362.2004 15576785 PMC532444

[B13] Diep B. A. Gill S. R. Chang R. F. Phan T. H. Chen J. H. Davidson M. G.. (2006). Complete genome sequence of USA300, an epidemic clone of community-acquired meticillin-resistant *Staphylococcus aureus*. Lancet 367, 731–739. 10.1016/S0140-673668231-7 16517273

[B14] Diep B. A. Phung Q. Date S. Arnott D. Bakalarski C. Xu M.. (2014). Identifying potential therapeutic targets of methicillin-resistant *Staphylococcus aureus* through *in vivo* proteomic analysis. J. Infect. Dis. 209, 1533–1541. 10.1093/infdis/jit662 24280367 PMC3997574

[B15] Gan K. Sankaran K. Williams M. G. Aldea M. Rudd K. E. Kushner S. R.. (1995). The *umpA* gene of *Escherichia coli* encodes phosphatidylglycerol:prolipoprotein diacylglyceryl transferase (lgt) and regulates thymidylate synthase levels through translational coupling. J. Bacteriol. 177, 1879–1882. 7896715 10.1128/jb.177.7.1879-1882.1995 PMC176820

[B16] Götz F. Mayer S. (2013). Both terminal oxidases contribute to fitness and virulence during organ-specific *Staphylococcus aureus* colonization. MBio 4, e00976–e00913. 10.1128/mBio.00976-13 24302255 PMC3870253

[B17] Grigg J. C. Vermeiren C. L. Heinrichs D. E. Murphy M. E. (2007). Heme coordination by *Staphylococcus aureus* IsdE. J. Biol. Chem. 282, 28815–28822. 10.1074/jbc.M704602200 17666394

[B18] Hamilton A. Robinson C. Sutcliffe I. C. Slater J. Maskell D. J. Davis-Poynter N.. (2006). Mutation of the maturase lipoprotein attenuates the virulence of *Streptococcus equi* to a greater extent than does loss of general lipoprotein lipidation. Infect. Immun. 74, 6907–6919. 10.1128/IAI.01116-06 17015455 PMC1698103

[B19] Hammer N. D. Reniere M. L. Cassat J. E. Zhang Y. Hirsch A. O. Indriati Hood M.. (2013). Two heme-dependent terminal oxidases power *Staphylococcus aureus* organ-specific colonization of the vertebrate host. MBio 4:e00241–13. 10.1128/mBio.00241-13 23900169 PMC3735196

[B20] Hantke K. Braun V. (1973). Covalent binding of lipid to protein. Diglyceride and amide-linked fatty acid at the N-terminal end of the murein-lipoprotein of the *Escherichia coli* outer membrane. Eur. J. Biochem. 34, 284–296. 4575979 10.1111/j.1432-1033.1973.tb02757.x

[B21] Heikkinen O. Seppala R. Tossavainen H. Heikkinen S. Koskela H. Permi P.. (2009). Solution structure of the parvulin-type PPIase domain of *Staphylococcus aureus* PrsA–implications for the catalytic mechanism of parvulins. BMC Struct. Biol. 9:17. 10.1186/1472-6807-9-17 19309529 PMC2678132

[B22] Heinrichs J. H. Gatlin L. E. Kunsch C. Choi G. H. Hanson M. S. (1999). Identification and characterization of SirA, an iron-regulated protein from *Staphylococcus aureus*. J. Bacteriol. 181, 1436–1443. 10049373 10.1128/jb.181.5.1436-1443.1999 PMC93531

[B23] Henneke P. Dramsi S. Mancuso G. Chraibi K. Pellegrini E. Theilacker C.. (2008). Lipoproteins are critical TLR2 activating toxins in group B streptococcal sepsis. J. Immunol. 180, 6149–6158. 10.4049/jimmunol.180.9.6149 18424736

[B24] Hiron A. Borezée-Durant E. Piard J. C. Juillard V. (2007). Only one of four oligopeptide transport systems mediates nitrogen nutrition in *Staphylococcus aureus*. J. Bacteriol. 189, 5119–5129. 10.1128/jb.00274-07 17496096 PMC1951871

[B25] Horsburgh M. J. Wharton S. J. Cox A. G. Ingham E. Peacock S. Foster S. J. (2002). MntR modulates expression of the PerR regulon and superoxide resistance in *Staphylococcus aureus* through control of manganese uptake. Mol. Microbiol. 44, 1269–1286. 10.1046/j.1365-2958.2002.02944.x 12028379

[B26] Hussain M. Ichihara S. Mizushima S. (1982). Mechanism of signal peptide cleavage in the biosynthesis of the major lipoprotein of the *Escherichia coli* outer membrane. J. Biol. Chem. 257, 5177–5182. 7040395

[B27] Jeong D. W. Cho H. Jones M. B. Shatzkes K. Sun F. Ji Q.. (2012). The auxiliary protein complex SaePQ activates the phosphatase activity of sensor kinase SaeS in the SaeRS two-component system of *Staphylococcus aureus*. Mol. Microbiol. 86, 331–348. 10.1111/j.1365-2958.2012.08198.x 22882143 PMC3468659

[B28] Jousselin A. Manzano C. Biette A. Reed P. Pinho M. Rosato A.. (2015). The *Staphylococcus aureus* chaperone PrsA is a new auxiliary factor of oxacillin resistance affecting Penicillin-binding protein 2A. Antimicrob. Agents Chemother. 60, 1656–1666. 10.1128/AAC.02333-15 26711778 PMC4775990

[B29] Jousselin A. Renzoni A. Andrey D. O. Monod A. Lew D. P. Kelley W. L. (2012). The posttranslocational chaperone lipoprotein PrsA is involved in both glycopeptide and oxacillin resistance in *Staphylococcus aureus*. Antimicrob. Agents Chemother. 56, 3629–3640. 10.1128/AAC.06264-11 22526301 PMC3393433

[B30] Juncker A. S. Willenbrock H. Von Heijne G. Brunak S. Nielsen H. Krogh A. (2003). Prediction of lipoprotein signal peptides in Gram-negative bacteria. Protein Sci. 12, 1652–1662. 10.1110/ps.0303703 12876315 PMC2323952

[B31] Kehl-Fie T. E. Zhang Y. Moore J. L. Farrand A. J. Hood M. I. Rathi S.. (2013). MntABC and MntH contribute to systemic *Staphylococcus aureus* infection by competing with calprotectin for nutrient manganese. Infect. Immun. 81, 3395–3405. 10.1128/IAI.00420-13 23817615 PMC3754211

[B32] Khandavilli S. Homer K. A. Yuste J. Basavanna S. Mitchell T. Brown J. S. (2008). Maturation of Streptococcus pneumoniae lipoproteins by a type II signal peptidase is required for ABC transporter function and full virulence. Mol. Microbiol. 67, 541–557. 10.1111/j.1365-2958.2007.06065.x 18086214 PMC2228790

[B33] Kouwen T. R. van der Goot A. Dorenbos R. Winter T. Antelmann H. Plaisier M. C.. (2007). Thiol-disulphide oxidoreductase modules in the low-GC Gram-positive bacteria. Mol. Microbiol. 64, 984–999. 10.1111/j.1365-2958.2007.05707.x 17501922

[B34] Kurokawa K. Kim M. S. Ichikawa R. Ryu K. H. Dohmae N. Nakayama H.. (2012). Environment-mediated accumulation of diacyl lipoproteins over their triacyl counterparts in *Staphylococcus aureus*. J. Bacteriol. 194, 3299–3306. 10.1128/JB.00314-12 22467779 PMC3434734

[B35] Kurokawa K. Lee H. Roh K. B. Asanuma M. Kim Y. S. Nakyama H.. (2009). The triacylated ATP binding cluster transporter substrate-binding lipoprotein of *Staphylococcus aureus* functions as a native ligand for the toll-like receptor 2. J. Biol. Chem. 284, 8406–8411. 10.1074/jbc.M809618200 19139093 PMC2659198

[B36] Machata S. Tchatalbachev S. Mohamed W. Jänsch L. Hain T. Chakraborty T. (2008). Lipoproteins of *Listeria monocytogenes* are critical for virulence and TLR2-mediated immune activation. J. Immunol. 181, 2028–2035. 10.4049/jimmunol.181.3.2028 18641340

[B37] Magadum S. Banerjee U. Murugan P. Gangapur D. Ravikesavan R. (2013). Gene duplication as a major force in evolution. J. Genet. 92, 155–161. 10.1007/s12041-013-0212-8 23640422

[B38] Makgotlho P. E. Marincola G. Schäfer D. Liu Q. Bae T. Geiger T.. (2013). SDS interferes with SaeS signaling of *Staphylococcus aureus* independently of SaePQ. PLoS ONE 8:e71644. 10.1371/journal.pone.0071644 23977102 PMC3748130

[B39] Mariotti P. Malito E. Biancucci M. Lo Surdo P. Mishra R. P. Nardi-Dei V.. (2013). Structural and functional characterization of the *Staphylococcus aureus* virulence factor and vaccine candidate FhuD2. Biochem. J. 449, 683–693. 10.1042/BJ20121426 23113737

[B40] Mazmanian S. K. Skaar E. P. Gaspar A. H. Humayun M. Gornicki P. Jelenska J.. (2003). Passage of heme-iron across the envelope of *Staphylococcus aureus*. Science 299, 906–909. 10.1126/science.1081147 12574635

[B41] Mazmanian S. K. Ton-That H. Schneewind O. (2001). Sortase-catalysed anchoring of surface proteins to the cell wall of *Staphylococcus aureus*. Mol. Microbiol. 40, 1049–1057. 10.1046/j.1365-2958.2001.02411.x 11401711

[B42] Mazmanian S. K. Ton-That H. Su K. Schneewind O. (2002). An iron-regulated sortase anchors a class of surface protein during *Staphylococcus aureus* pathogenesis. Proc. Natl. Acad. Sci. U.S.A. 99, 2293–2298. 10.1073/pnas.032523999 11830639 PMC122358

[B43] Merlin C. Gardiner G. Durand S. Masters M. (2002). The *Escherichia coli metD* locus encodes an ABC transporter which includes Abc (MetN), YaeE (MetI), and YaeC (MetQ). J. Bacteriol. 184, 5513–5517. 10.1128/JB.184.19.5513-5517.2002 12218041 PMC135343

[B44] Morrissey J. A. Cockayne A. Hill P. J. Williams P. (2000). Molecular cloning and analysis of a putative siderophore ABC transporter from *Staphylococcus aureus*. Infect. Immun. 68, 6281–6288. 10.1128/IAI.68.11.6281-6288.2000 11035736 PMC97710

[B45] Müller P. Müller-Anstett M. Wagener J. Gao Q. Kaesler S. Schaller M.. (2010). The *Staphylococcus aureus* lipoprotein SitC colocalizes with Toll-like receptor 2 (TLR2) in murine keratinocytes and elicits intracellular TLR2 accumulation. Infect. Immun. 78, 4243–4250. 10.1128/IAI.00538-10 20679445 PMC2950364

[B46] Neubauer H. Pantel I. Lindgren P. E. Götz F. (1999). Characterization of the molybdate transport system ModABC of *Staphylococcus carnosus*. Arch. Microbiol. 172, 109–115. 10415172 10.1007/s002030050747

[B47] Nguyen M. T. Götz F. (2016). Lipoproteins of Gram-Positive bacteria: key players in the immune response and virulence. Microbiol. Mol. Biol. Rev. 80, 891–903. 10.1128/MMBR.00028-16 27512100 PMC4981669

[B48] Nguyen M. T. Hanzelmann D. Hartner T. Peschel A. Götz F. (2015a). Skin-specific unsaturated fatty acids boost the *Staphylococcus aureus* innate immune response. Infect. Immun. 84, 205–215. 10.1128/IAI.00822-15 26502910 PMC4693987

[B49] Nguyen M. T. Kraft B. Yu W. Demicrioglu D. D. Hertlein T. Burian M.. (2015b). The νSaα specific Lipoprotein Like Cluster (lpl) of *S. aureus* USA300 contributes to immune stimulation and invasion in human cells. PLoS Pathog. 11:e1004984. 10.1371/journal.ppat.1004984 26083414 PMC4470592

[B50] Nielsen J. B. Lampen J. O. (1982a). Glyceride-cysteine lipoproteins and secretion by Gram-positive bacteria. J. Bacteriol. 152, 315–322. 6811555 10.1128/jb.152.1.315-322.1982 PMC221407

[B51] Nielsen J. B. Lampen J. O. (1982b). Membrane-bound penicillinases in Gram-positive bacteria. J. Biol. Chem. 257, 4490–4495. 6802832

[B52] Petit C. M. Brown J. R. Ingraham K. Bryant A. P. Holmes D. J. (2001). Lipid modification of prelipoproteins is dispensable for growth *in vitro* but essential for virulence in *Streptococcus pneumoniae*. FEMS Microbiol. Lett. 200, 229–233. 10.1111/j.1574-6968.2001.tb10720.x 11425480

[B53] Podkowa K. J. Briere L. A. Heinrichs D. E. Shilton B. H. (2014). Crystal and solution structure analysis of FhuD2 from *Staphylococcus aureus* in multiple unliganded conformations and bound to ferrioxamine-B. Biochemistry 53, 2017–2031. 10.1021/bi401349d 24606332

[B54] Rahman O. Cummings S. P. Harrington D. J. Sutcliffe I. C. (2008). Methods for the bioinformatic identification of bacterial lipoproteins encoded in the genomes of Gram-positive bacteria. World J. Microbiol. Biotechnol. 24, 2377–2382. 10.1007/s11274-008-9795-2

[B55] Remy L. Carrière M. Derré-Bobillot A. Martini C. Sanguinetti M. Borezee-Durant E. (2013). The *Staphylococcus aureus* Opp1 ABC transporter imports nickel and cobalt in zinc-depleted conditions and contributes to virulence. Mol. Microbiol. 87, 730–743. 10.1111/mmi.12126 23279021

[B56] Robinson D. A. Kearns A. M. Holmes A. Morrison D. Grundmann H. Edwards G.. (2005). Re-emergence of early pandemic *Staphylococcus aureus* as a community-acquired meticillin-resistant clone. Lancet 365, 1256–1258. 10.1016/S0140-673674814-5 15811459

[B57] Sander P. Rezwan M. Walker B. Rampini S. K. Kroppenstedt R. M. Ehlers S.. (2004). Lipoprotein processing is required for virulence of *Mycobacterium tuberculosis*. Mol. Microbiol. 52, 1543–1552. 10.1111/j.1365-2958.2004.04041.x 15186407

[B58] Sankaran K. Wu H. C. (1994). Lipid modification of bacterial prolipoprotein. Transfer of diacylglyceryl moiety from phosphatidylglycerol. J. Biol. Chem. 269, 19701–19706. 8051048

[B59] Schluepen C. Malito E. Marongiu A. Schirle M. McWhinnie E. Lo Surdo P.. (2013). Mining the bacterial unknown proteome: identification and characterization of a novel family of highly conserved protective antigens in *Staphylococcus aureus*. Biochem. J. 455, 273–284. 10.1042/BJ20130540 23895222

[B60] Schmaler M. Jann N. J. Ferracin F. Landolt L. Z. Biswas L. Götz F.. (2009). Lipoproteins in *Staphylococcus aureus* mediate inflammation by TLR2 and iron-dependent growth *in vivo*. J. Immunol. 182, 7110–7118. 10.4049/jimmunol.0804292 19454708

[B61] Schmaler M. Jann N. J. Götz F. Landmann R. (2010). Staphylococcal lipoproteins and their role in bacterial survival in mice. Int. J. Med. Microbiol. 300, 155–160. 10.1016/j.ijmm.2009.08.018 19805005

[B62] Schmollinger M. Fischer I. Nerz C. Pinkenburg S. Götz F. Kaufmann M.. (2004). ParSeq: searching motifs with structural and biochemical properties. Bioinformatics 20, 1459–1461. 10.1093/bioinformatics/bth083 14962930

[B63] Sebulsky M. T. Heinrichs D. E. (2001). Identification and characterization of fhuD1 and fhuD2, two genes involved in iron-hydroxamate uptake in *Staphylococcus aureus*. J. Bacteriol. 183, 4994–5000. 10.1128/JB.183.17.4994-5000.2001 11489851 PMC95374

[B64] Sebulsky M. T. Hohnstein D. Hunter M. D. Heinrichs D. E. (2000). Identification and characterization of a membrane permease involved in iron-hydroxamate transport in *Staphylococcus aureus*. J. Bacteriol. 182, 4394–4400. 10.1128/JB.182.16.4394-4400.2000 10913070 PMC94608

[B65] Sebulsky M. T. Speziali C. D. Shilton B. H. Edgell D. R. Heinrichs D. E. (2004). FhuD1, a ferric hydroxamate-binding lipoprotein in *Staphylococcus aureus*: a case of gene duplication and lateral transfer. J. Biol. Chem. 279, 53152–53159. 10.1074/jbc.M409793200 15475351

[B66] Sibbald M. J. Ziebandt A. K. Engelmann S. Hecker M. de Jong A. Harmsen H. J.. (2006). Mapping the pathways to staphylococcal pathogenesis by comparative secretomics. Microbiol. Mol. Biol. Rev. 70, 755–788. 10.1128/MMBR.00008-06 16959968 PMC1594592

[B67] Skabytska Y. Wölbing F. Gunther C. Koberle M. Kaesler S. Chen K. M.. (2014). Cutaneous innate immune sensing of Toll-like receptor 2–6 ligands suppresses T cell immunity by inducing myeloid-derived suppressor cells. Immunity 41, 762–775. 10.1016/j.immuni.2014.10.009 25456159

[B68] Stoll H. Dengjel J. Nerz C. Götz F. (2005). *Staphylococcus aureus* deficient in lipidation of prelipoproteins is attenuated in growth and immune activation. Infect. Immun. 73, 2411–2423. 10.1128/IAI.73.4.2411-2423.2005 15784587 PMC1087423

[B69] Sutcliffe I. C. Harrington D. J. (2002). Pattern searches for the identification of putative lipoprotein genes in Gram-positive bacterial genomes. Microbiology 148(Pt 7), 2065–2077. 10.1099/00221287-148-7-2065 12101295

[B70] Sutcliffe I. C. Harrington D. J. Hutchings M. I. (2012). A phylum level analysis reveals lipoprotein biosynthesis to be a fundamental property of bacteria. Protein Cell 3, 163–170. 10.1007/s13238-012-2023-8 22410786 PMC4875425

[B71] Takeda K. Takeuchi O. Akira S. (2002). Recognition of lipopeptides by Toll-like receptors. J. Endotoxin Res. 8, 459–463. 10.1179/096805102125001073 12697090

[B72] Takeuchi O. Kawai T. Mühlradt P. F. Morr M. Radolf J. D. Zychlinsky A.. (2001). Discrimination of bacterial lipoproteins by Toll-like receptor 6. Int. Immunol. 13, 933–940. 10.1093/intimm/13.7.933 11431423

[B73] Takeuchi O. Sato S. Horiuchi T. Hoshino K. Takeda K. Dong Z.. (2002). Cutting edge: role of Toll-like receptor 1 in mediating immune response to microbial lipoproteins. J. Immunol. 169, 10–14. 10.4049/jimmunol.169.1.10 12077222

[B74] Taylor P. D. Toseland C. P. Attwood T. K. Flower D. R. (2006). LIPPRED: A web server for accurate prediction of lipoprotein signal sequences and cleavage sites. Bioinformation 1, 176–179. 10.6026/97320630001176 17597883 PMC1891677

[B75] von Heijne G. (1989). The structure of signal peptides from bacterial lipoproteins. Protein Eng. 2, 531–534. 2664762 10.1093/protein/2.7.531

[B76] Vu C. H. Kolata J. Stentzel S. Beyer A. Salazar M. G. Steil L.. (2016). Adaptive immune response to lipoproteins of *Staphylococcus aureus* in healthy subjects. Proteomics. [Epub ahead of print]. 10.1002/pmic.201600151. 27324828 PMC5096053

[B77] Wang P. Dalbey R. E. (2011). Inserting membrane proteins: the YidC/Oxa1/Alb3 machinery in bacteria, mitochondria, and chloroplasts. Biochim. Biophys. Acta 1808, 866–875. 10.1016/j.bbamem.2010.08.014 20800571

[B78] Weston B. F. Brenot A. Caparon M. G. (2009). The metal homeostasis protein, Lsp, of *Streptococcus pyogenes* is necessary for acquisition of zinc and virulence. Infect. Immun. 77, 2840–2848. 10.1128/IAI.01299-08 19398546 PMC2708593

[B79] Williams W. A. Zhang R. G. Zhou M. Joachimiak G. Gornicki P. Missiakas D.. (2004). The membrane-associated lipoprotein-9 GmpC from *Staphylococcus aureus* binds the dipeptide GlyMet via side chain interactions. Biochemistry 43, 16193–16202. 10.1021/bi048877o 15610013 PMC2792005

[B80] Zähringer U. Lindner B. Inamura S. Heine H. Alexander C. (2008). TLR2 — promiscuous or specific? A critical re-evaluation of a receptor expressing apparent broad specificity. Immunobiology 213, 205–224. 10.1016/j.imbio.2008.02.005 18406368

