# Supplementary material for: Evaluation of Staphylococcus aureus Lipoproteins: Role in Nutritional Acquisition and Pathogenicity
Source: Front Microbiol. 2016 Sep 13;7:1404. doi: 10.3389/fmicb.2016.01404 (PMC5020093; doi:10.3389/fmicb.2016.01404)
Supplement: Supplementary file 1 [file Image1.PDF]

(A)

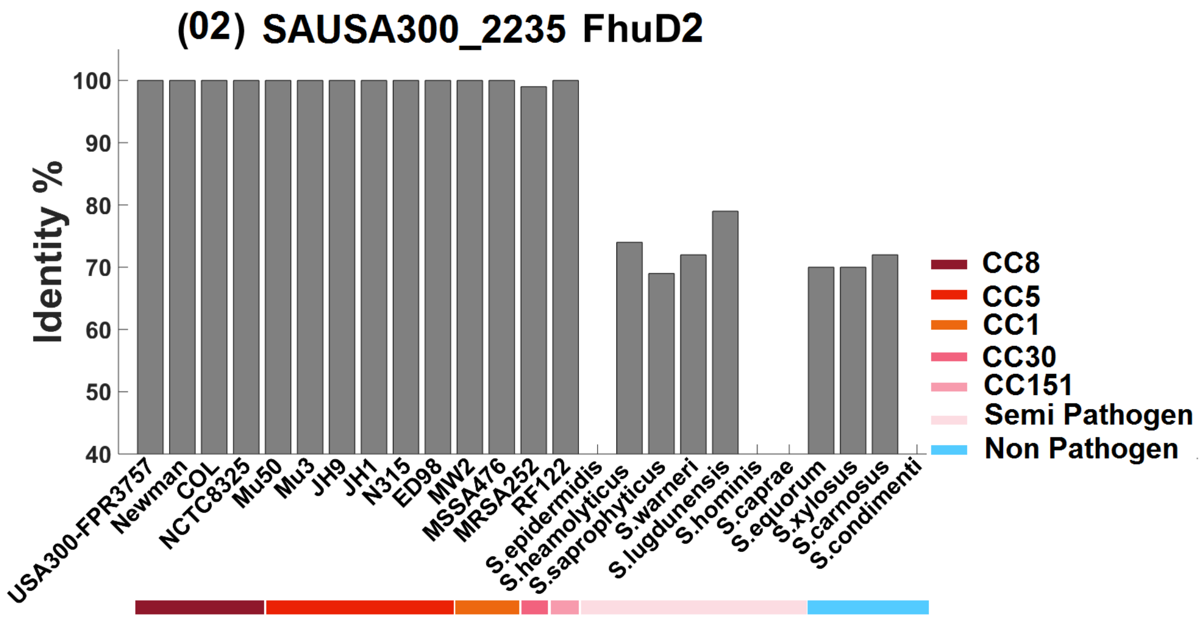
(02) SAUSA300_2235 FhuD2
Identity %
100
90
80
70
60
50
40
USA300-FPR3757
Newman
COL
NCTC8325
Mu50
Mu3
JH9
JH1
N315
ED98
MW2
MSSA476
MRSA252
RF122
S.epidermidis
S.heamolyticus
S.saprophyticus
S.warneri
S.lugdunensis
S.hominis
S.caprae
S.equorum
S.xylosus
S.carnosus
S.condimenti
CC8
CC5
CC1
CC30
CC151
Semi Pathogen
Non Pathogen

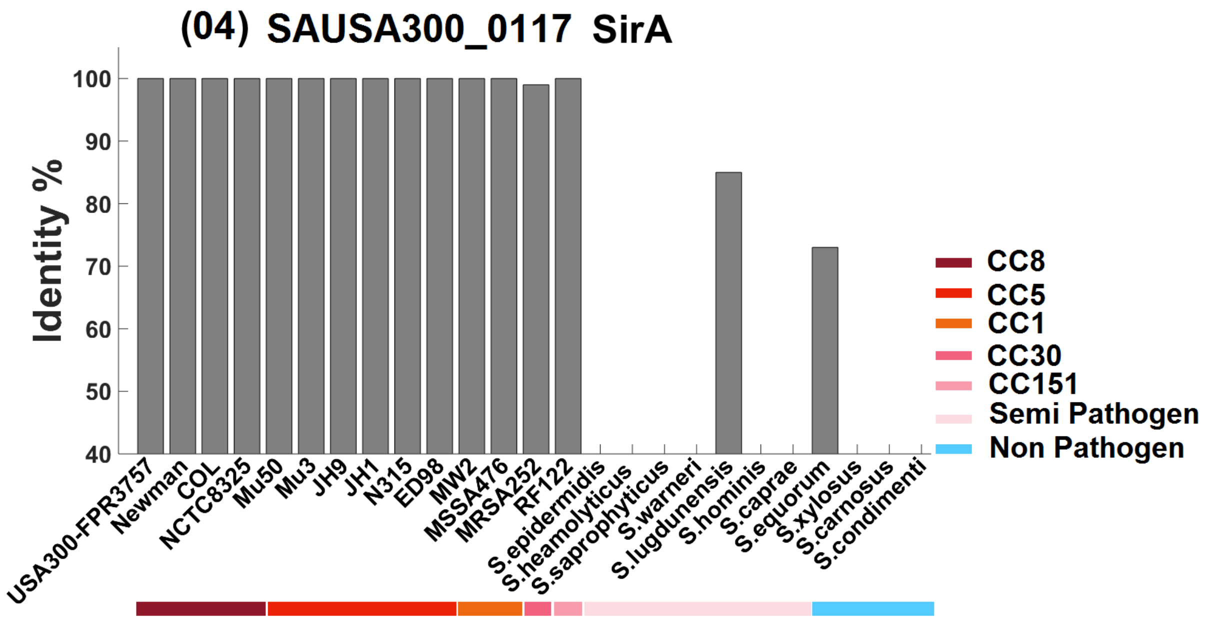
(04) SAUSA300_0117 SirA
Identity %
100
90
80
70
60
50
40
USA300-FPR3757
Newman
COL
NCTC8325
Mu50
Mu3
JH9
JH1
N315
ED98
MW2
MSSA476
MRSA252
RF122
S.epidermidis
S.heamolyticus
S.saprophyticus
S.warneri
S.lugdunensis
S.hominis
S.caprae
S.equorum
S.xylosus
S.carnosus
S.condimenti
CC8
CC5
CC1
CC30
CC151
Semi Pathogen
Non Pathogen

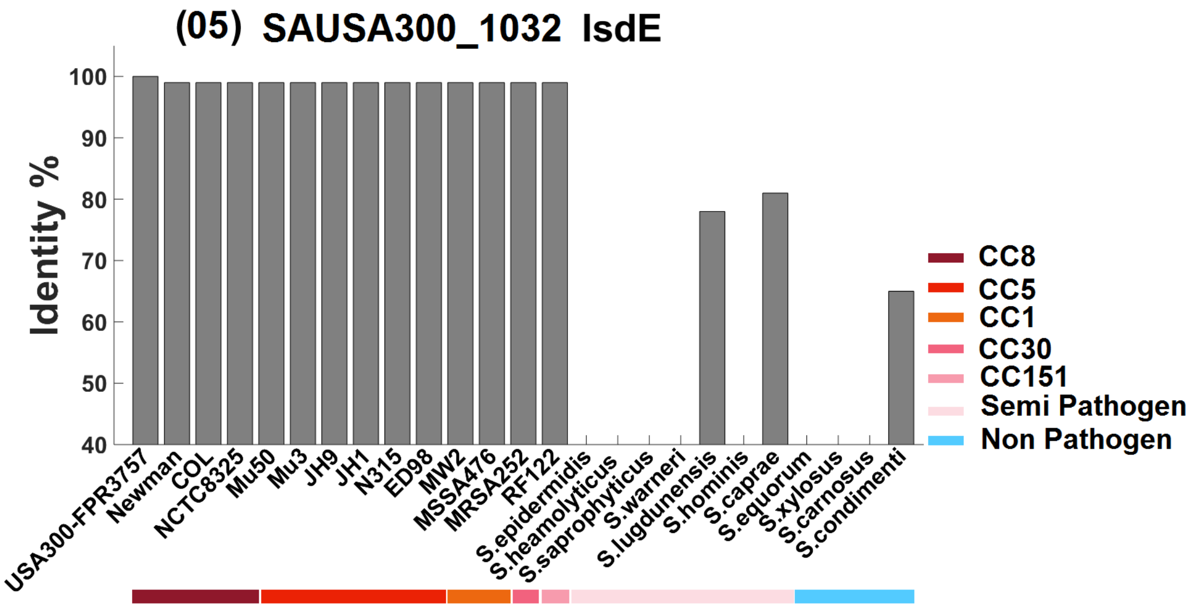
(05) SAUSA300_1032 IsdE
Identity %
100
90
80
70
60
50
40
USA300-FPR3757
Newman
COL
NCTC8325
Mu50
Mu3
JH9
JH1
N315
ED98
MW2
MSSA476
MRSA252
RF122
S.epidermidis
S.heamolyticus
S.saprophyticus
S.warneri
S.lugdunensis
S.hominis
S.caprae
S.equorum
S.xylosus
S.carnosus
S.condimenti
CC8
CC5
CC1
CC30
CC151
Semi Pathogen
Non Pathogen

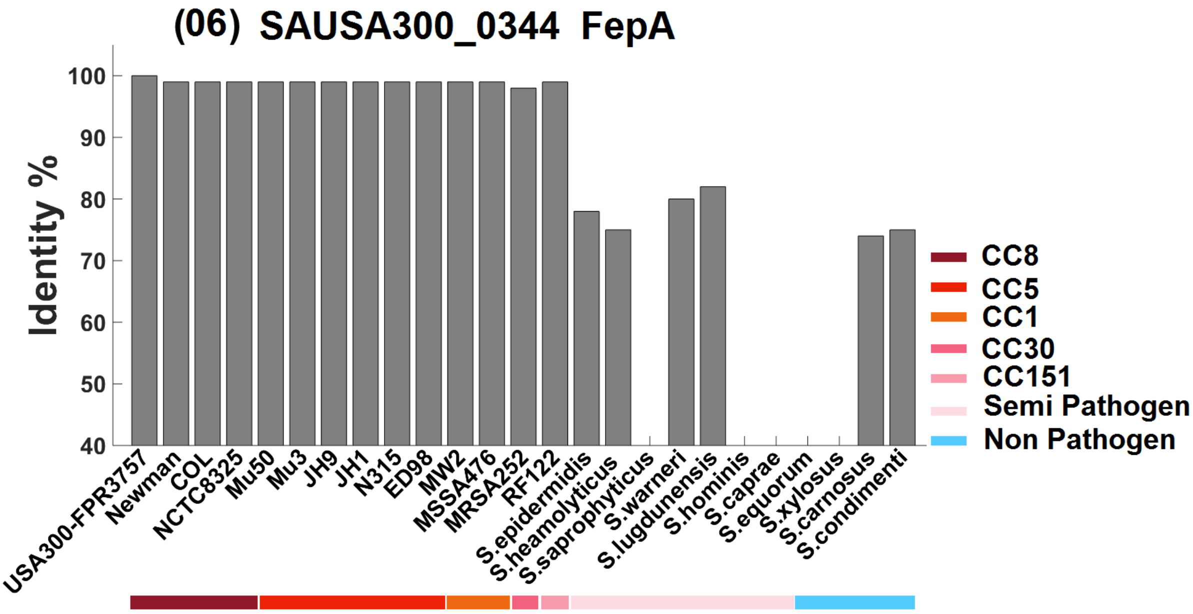
(06) SAUSA300_0344 FepA
Identity %
100
90
80
70
60
50
40
USA300-FPR3757
Newman
COL
NCTC8325
Mu50
Mu3
JH9
JH1
N315
ED98
MW2
MSSA476
MRSA252
RF122
S.epidermidis
S.heamolyticus
S.saprophyticus
S.warneri
S.lugdunensis
S.hominis
S.caprae
S.equorum
S.xylosus
S.carnosus
S.condimenti
CC8
CC5
CC1
CC30
CC151
Semi Pathogen
Non Pathogen

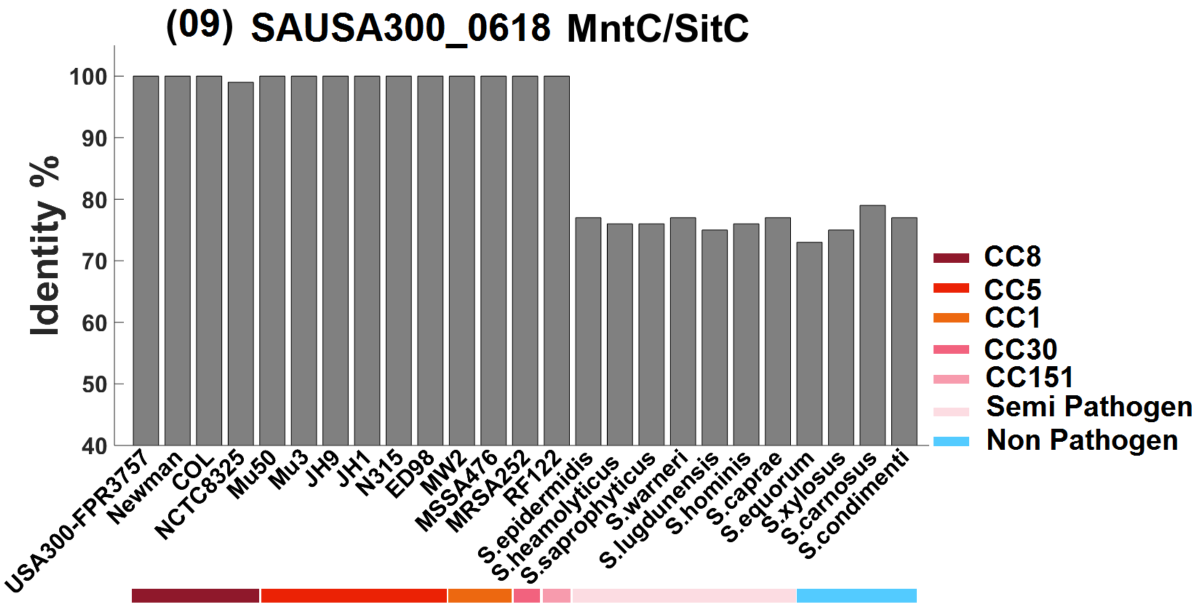

(09) SAUSA300_0618 MntC/SitC
Identity %
100
90
80
70
60
50
40
USA300-FPR3757
Newman
COL
NCTC8325
Mu50
Mu3
JH9
JH1
N315
ED98
MW2
MSSA476
MRSA252
RF122
S.epidermidis
S.heamolyticus
S.saprophyticus
S.warneri
S.lugdunensis
S.hominis
S.caprae
S.equorum
S.xylosus
S.carnosus
S.condimenti
CC8
CC5
CC1
CC30
CC151
Semi Pathogen
Non Pathogen

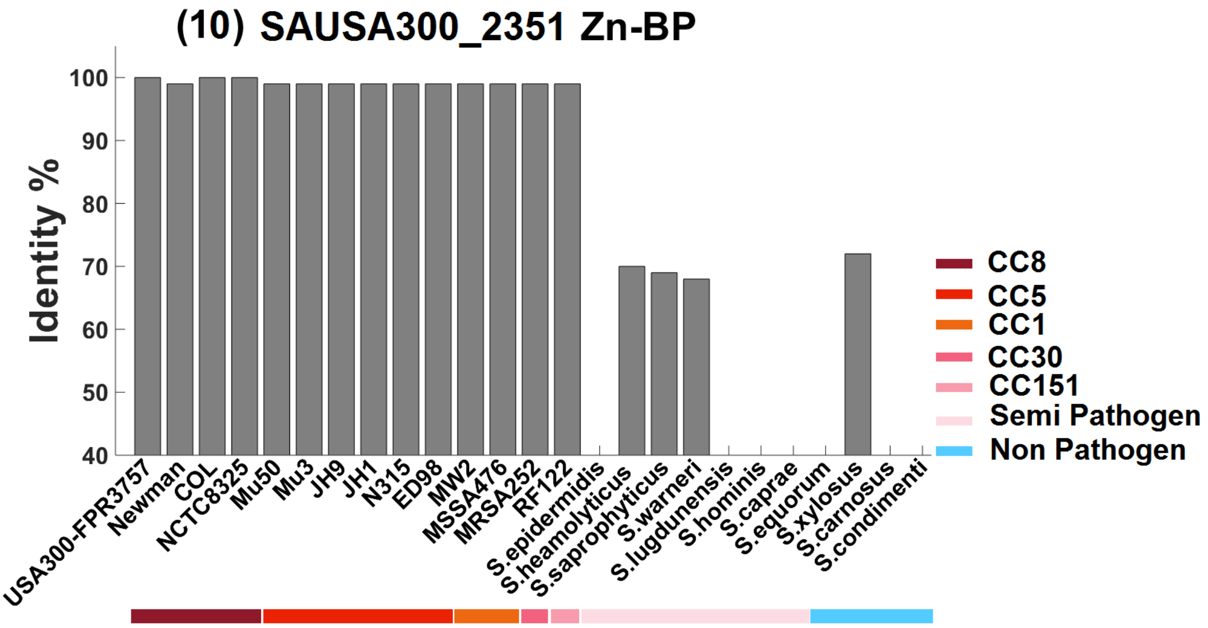

(10) SAUSA300_2351 Zn-BP
Identity %
100
90
80
70
60
50
40
USA300-FPR3757
Newman
COL
NCTC8325
Mu50
Mu3
JH9
JH1
N315
ED98
MW2
MSSA476
MRSA252
RF122
S.epidermidis
S.heamolyticus
S.saprophyticus
S.warneri
S.lugdunensis
S.hominis
S.caprae
S.equorum
S.xylosus
S.carnosus
S.condimenti
CC8
CC5
CC1
CC30
CC151
Semi Pathogen
Non Pathogen

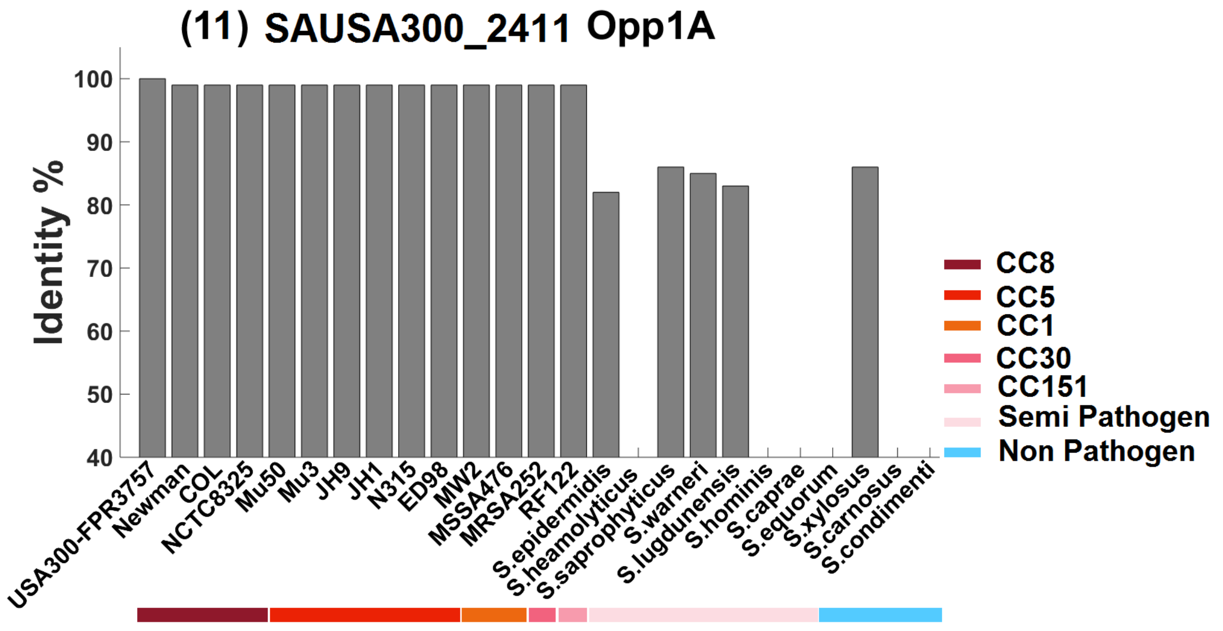
(11) SAUSA300_2411 Opp1A
Identity %
100
90
80
70
60
50
40
USA300-FPR3757
Newman
COL
NCTC8325
Mu50
Mu3
JH9
JH1
N315
ED98
MW2
MSSA476
MRSA252
RF122
S.epidermidis
S.heamolyticus
S.saprophyticus
S.warneri
S.lugdunensis
S.hominis
S.caprae
S.equorum
S.xylosus
S.carnosus
S.condimenti
CC8
CC5
CC1
CC30
CC151
Semi Pathogen
Non Pathogen

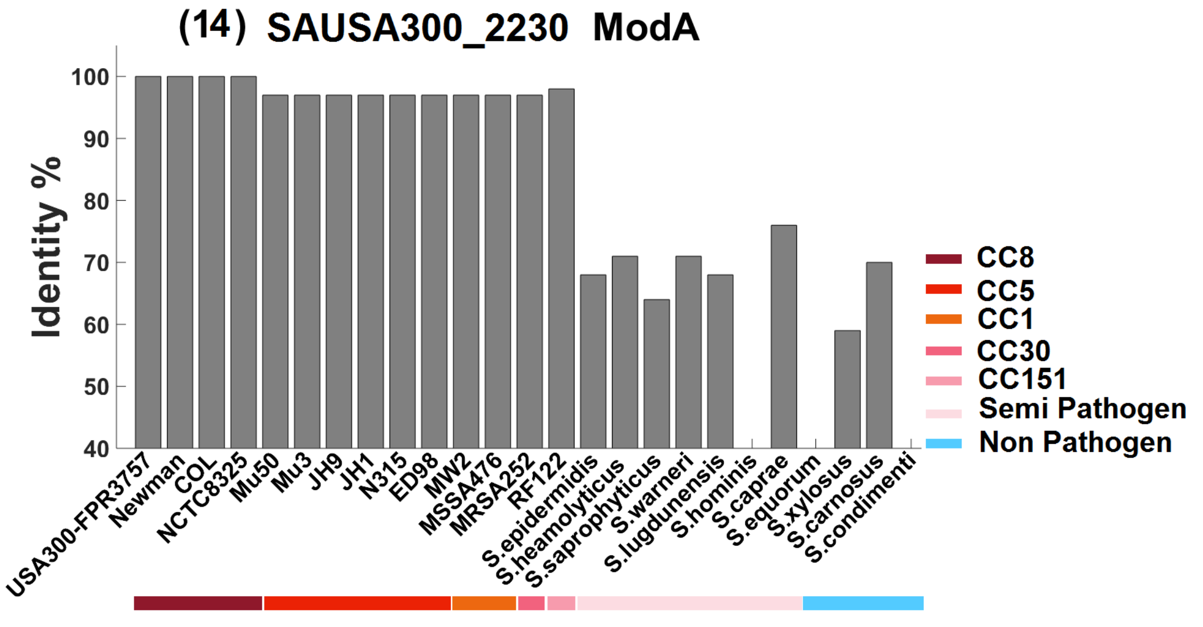
(14) SAUSA300_2230 ModA
Identity %
100
90
80
70
60
50
40
USA300-FPR3757
Newman
COL
NCTC8325
Mu50
Mu3
JH9
JH1
N315
ED98
MW2
MSSA476
MRSA252
RF122
S.epidermidis
S.heamolyticus
S.saprophyticus
S.warneri
S.lugdunensis
S.hominis
S.caprae
S.equorum
S.xylosus
S.carnosus
S.condimenti
CC8
CC5
CC1
CC30
CC151
Semi Pathogen
Non Pathogen

(15) SAUSA300_1283 PO_4^{2-} transp

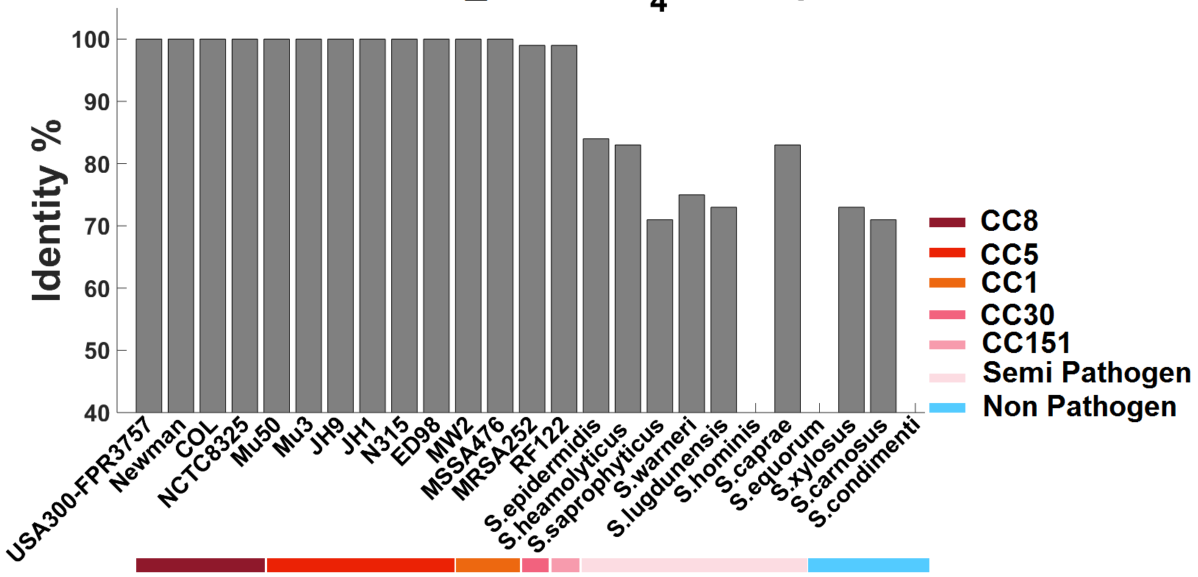

(16) SAUSA300_0145 PO_3^{2-} transp

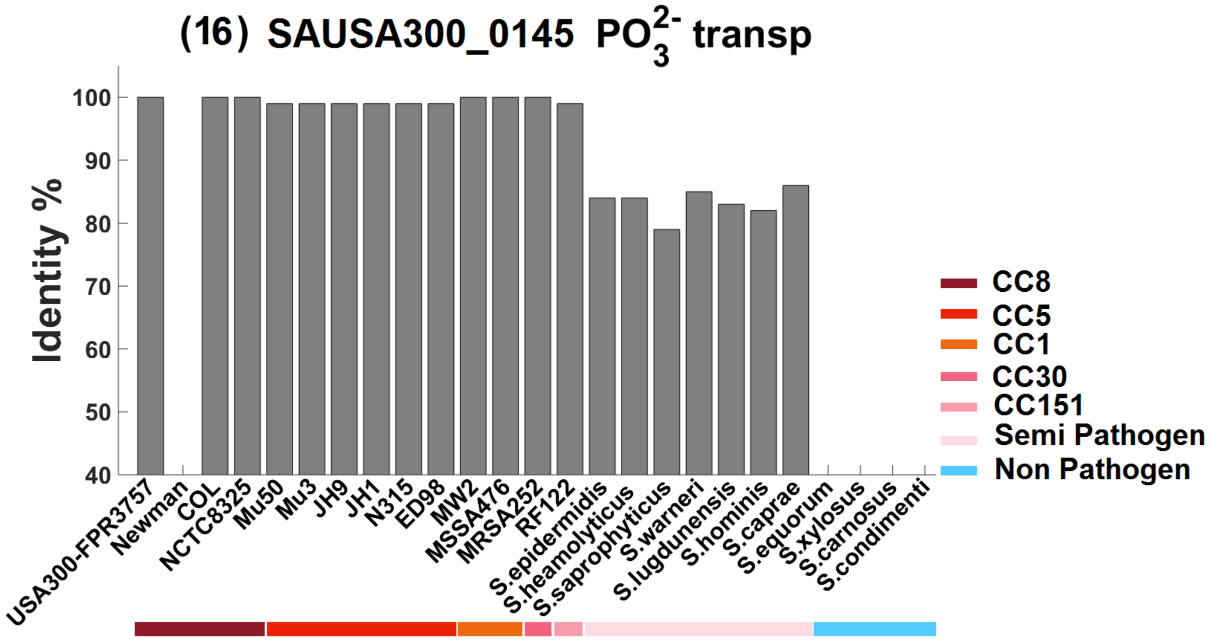

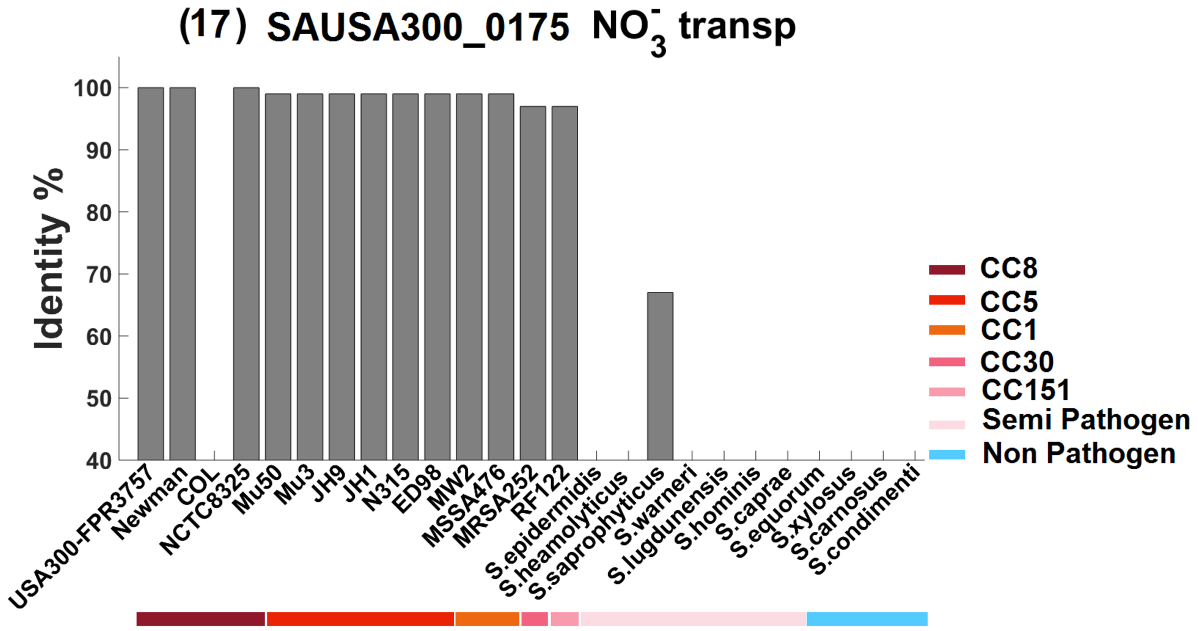

(17) SAUSA300_0175 NO_3^- transp
Identity %
100
90
80
70
60
50
40
USA300-FPR3757
Newman
COL
NCTC8325
Mu50
Mu3
JH9
JH1
N315
ED98
MW2
MSSA476
MRSA252
RF122
S.epidermidis
S.heamolyticus
S.saprophyticus
S.warneri
S.lugdunensis
S.hominis
S.caprae
S.equorum
S.xylosus
S.carnosus
S.condimenti
CC8
CC5
CC1
CC30
CC151
Semi Pathogen
Non Pathogen

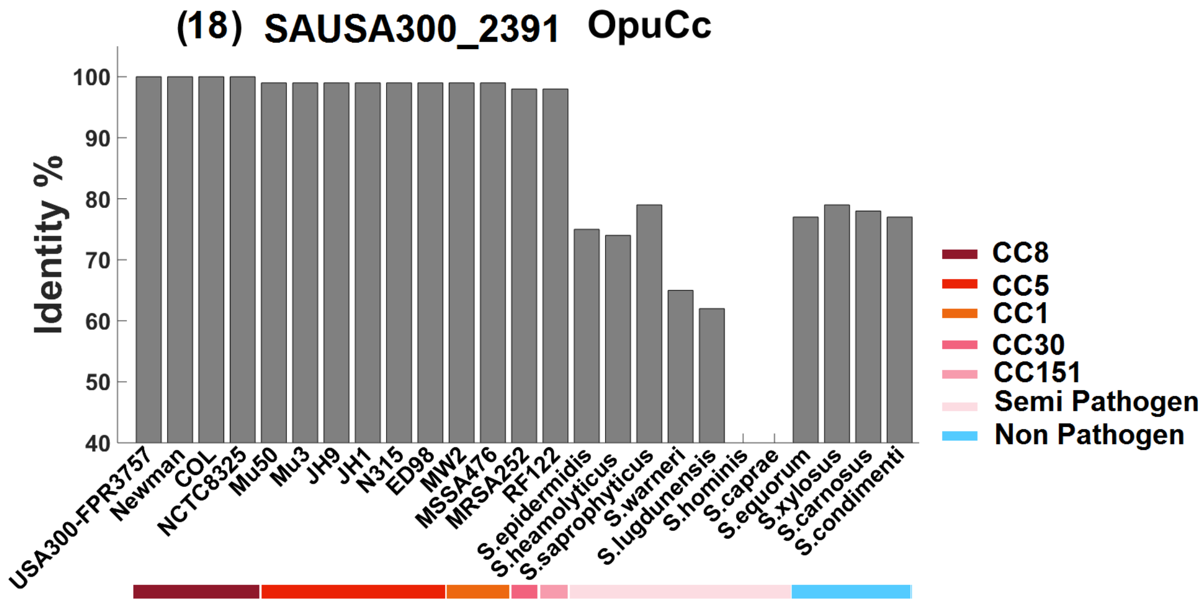

(18) SAUSA300_2391 OpuCc
Identity %
100
90
80
70
60
50
40
USA300-FPR3757
Newman
COL
NCTC8325
Mu50
Mu3
JH9
JH1
N315
ED98
MW2
MSSA476
MRSA252
RF122
S.epidermidis
S.heamolyticus
S.saprophyticus
S.warneri
S.lugdunensis
S.hominis
S.caprae
S.equorum
S.xylosus
S.carnosus
S.condimenti
CC8
CC5
CC1
CC30
CC151
Semi Pathogen
Non Pathogen

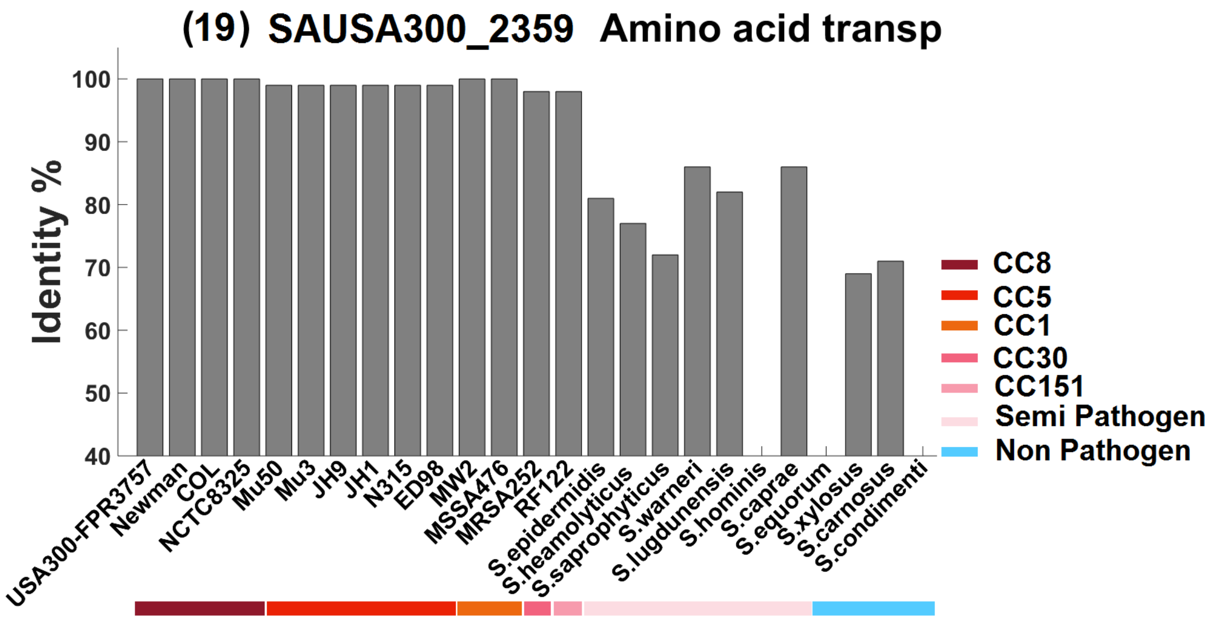
(19) SAUSA300_2359 Amino acid transp
Identity %
100
90
80
70
60
50
40
USA300-FPR3757
Newman
COL
NCTC8325
Mu50
Mu3
JH9
JH1
N315
ED98
MW2
MSSA476
MRSA252
RF122
S.epidermidis
S.heamolyticus
S.saprophyticus
S.warneri
S.lugdunensis
S.hominis
S.caprae
S.equorum
S.xylosus
S.carnosus
S.condimenti
CC8
CC5
CC1
CC30
CC151
Semi Pathogen
Non Pathogen

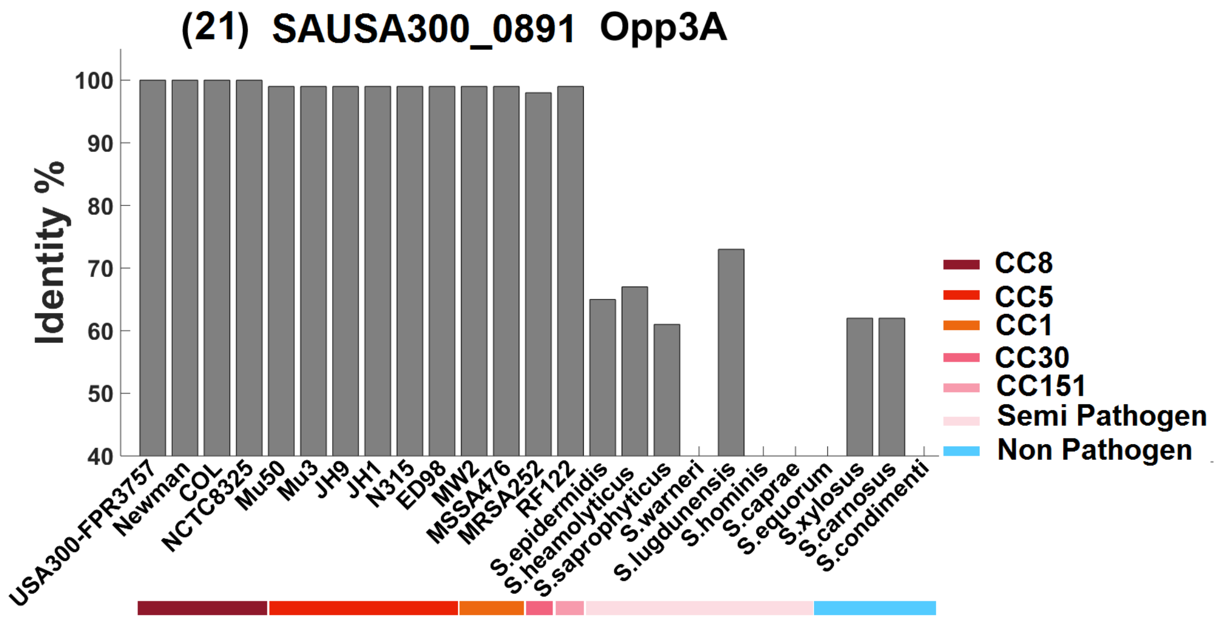
(21) SAUSA300_0891 Opp3A
Identity %
100
90
80
70
60
50
40
USA300-FPR3757
Newman
COL
NCTC8325
Mu50
Mu3
JH9
JH1
N315
ED98
MW2
MSSA476
MRSA252
RF122
S.epidermidis
S.heamolyticus
S.saprophyticus
S.warneri
S.lugdunensis
S.hominis
S.caprae
S.equorum
S.xylosus
S.carnosus
S.condimenti
CC8
CC5
CC1
CC30
CC151
Semi Pathogen
Non Pathogen

(23) SAUSA300_0437 GmpC

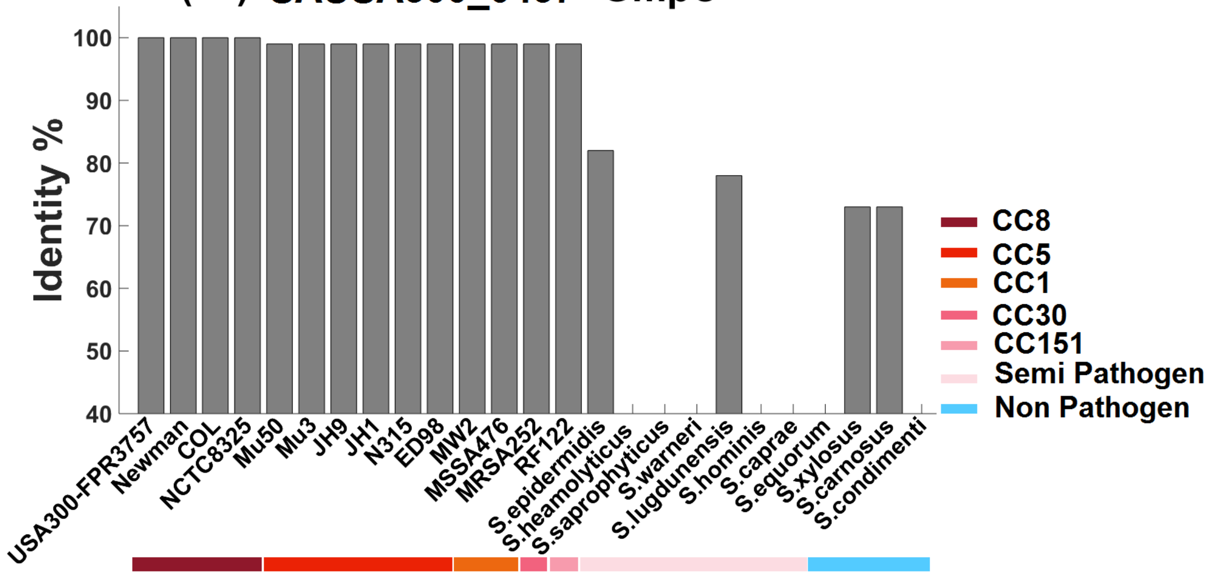

(24) SAUSA300_0798 D-Met transp

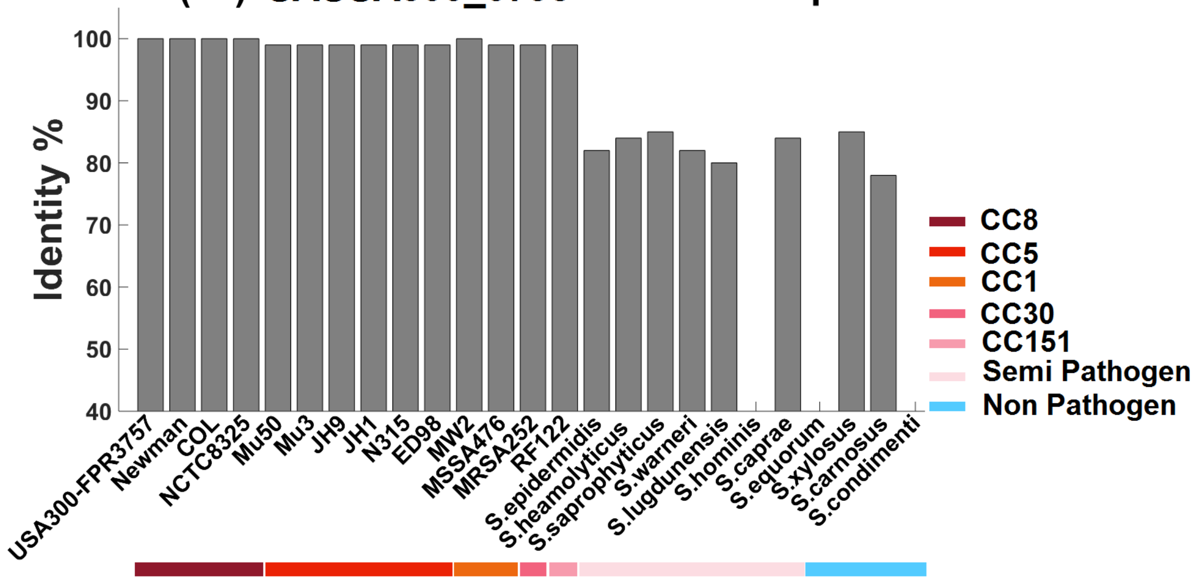

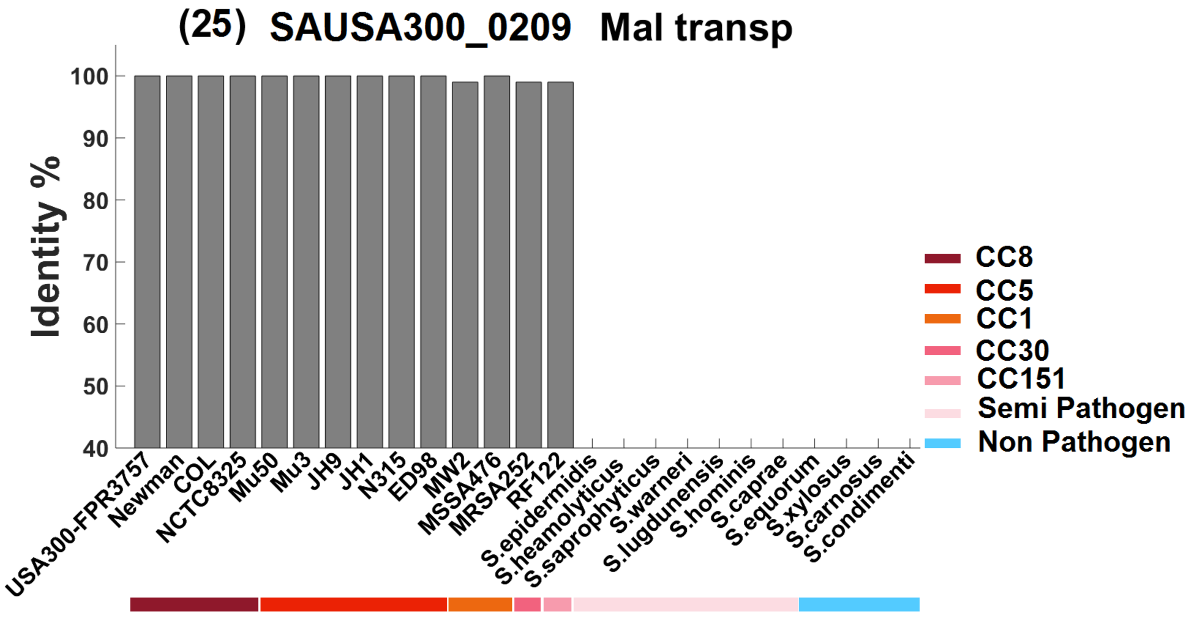
(25) SAUSA300_0209 Mal transp
Identity %
100
90
80
70
60
50
40
USA300-FPR3757
Newman
COL
NCTC8325
Mu50
Mu3
JH9
JH1
N315
ED98
MW2
MSSA476
MRSA252
RF122
S.epidermidis
S.heamolyticus
S.saprophyticus
S.warneri
S.lugdunensis
S.hominis
S.caprae
S.equorum
S.xylosus
S.carnosus
S.condimenti
CC8
CC5
CC1
CC30
CC151
Semi Pathogen
Non Pathogen

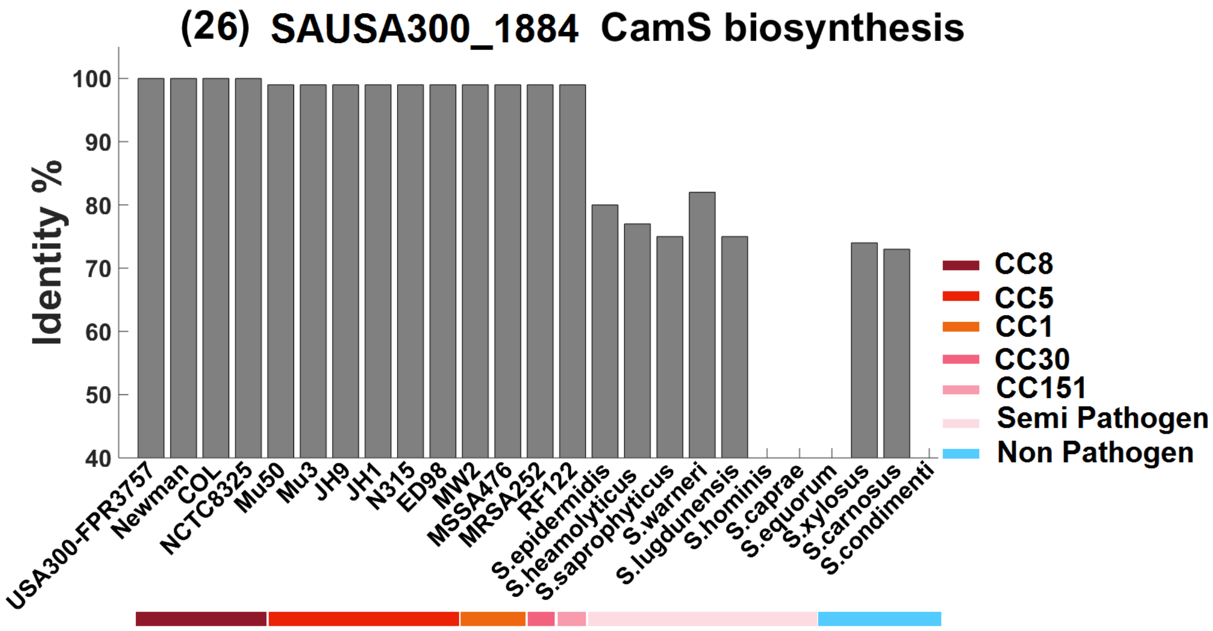
(26) SAUSA300_1884 CamS biosynthesis
Identity %
100
90
80
70
60
50
40
USA300-FPR3757
Newman
COL
NCTC8325
Mu50
Mu3
JH9
JH1
N315
ED98
MW2
MSSA476
MRSA252
RF122
S.epidermidis
S.heamolyticus
S.saprophyticus
S.warneri
S.lugdunensis
S.hominis
S.caprae
S.equorum
S.xylosus
S.carnosus
S.condimenti
CC8
CC5
CC1
CC30
CC151
Semi Pathogen
Non Pathogen

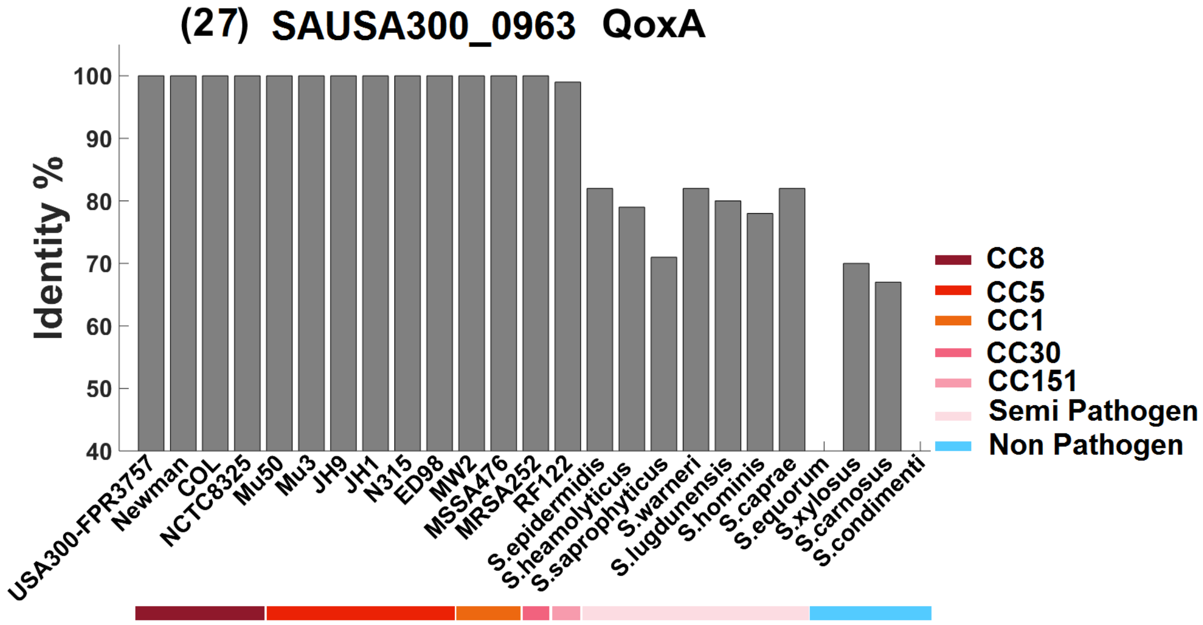

(27) SAUSA300_0963 QoxA
Identity %
100
90
80
70
60
50
40
USA300-FPR3757
Newman
COL
NCTC8325
Mu50
Mu3
JH9
JH1
N315
ED98
MW2
MSSA476
MRSA252
RF122
S.epidermidis
S.heamolyticus
S.saprophyticus
S.warneri
S.lugdunensis
S.hominis
S.caprae
S.equorum
S.xylosus
S.carnosus
S.condimenti
CC8
CC5
CC1
CC30
CC151
Semi Pathogen
Non Pathogen

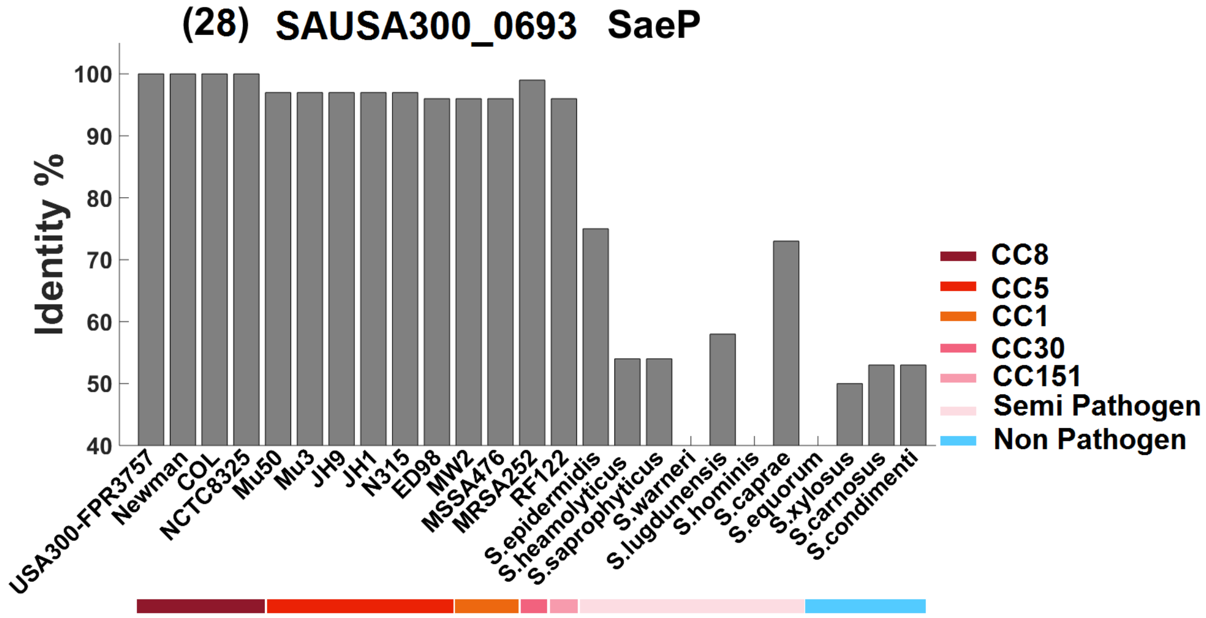

(28) SAUSA300_0693 SaeP
Identity %
100
90
80
70
60
50
40
USA300-FPR3757
Newman
COL
NCTC8325
Mu50
Mu3
JH9
JH1
N315
ED98
MW2
MSSA476
MRSA252
RF122
S.epidermidis
S.heamolyticus
S.saprophyticus
S.warneri
S.lugdunensis
S.hominis
S.caprae
S.equorum
S.xylosus
S.carnosus
S.condimenti
CC8
CC5
CC1
CC30
CC151
Semi Pathogen
Non Pathogen

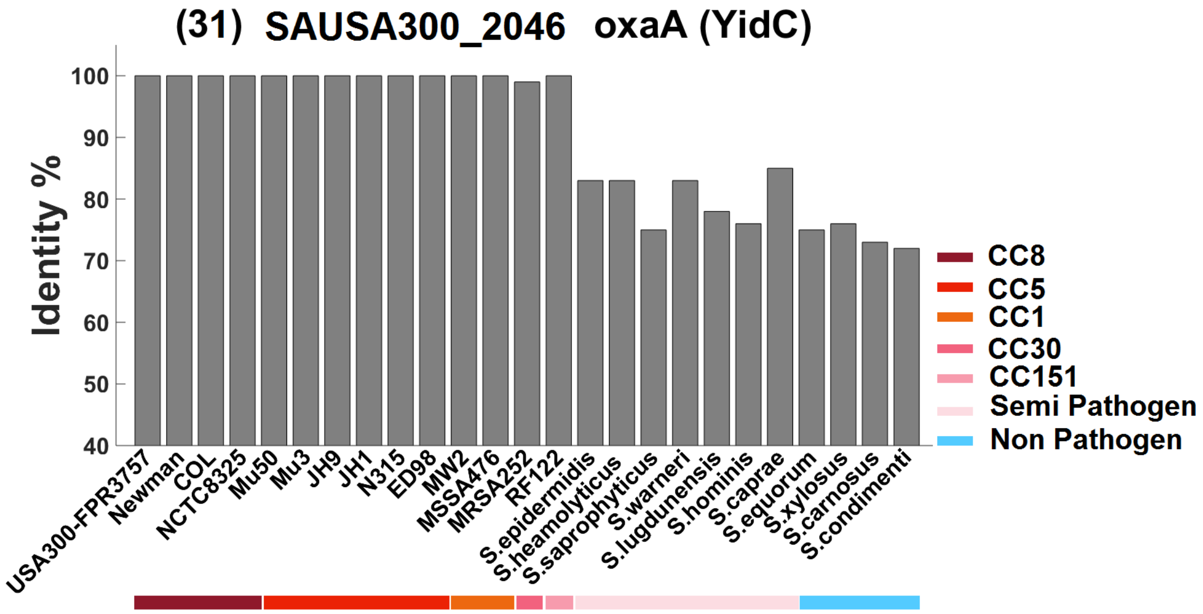
(31) SAUSA300_2046 oxaA (YidC)
Identity %
100
90
80
70
60
50
40
USA300-FPR3757
Newman
COL
NCTC8325
Mu50
Mu3
JH9
JH1
N315
ED98
MW2
MSSA476
MRSA252
RF122
S.epidermidis
S.heamolyticus
S.saprophyticus
S.warneri
S.lugdunensis
S.hominis
S.caprae
S.equorum
S.xylosus
S.carnosus
S.condimenti
CC8
CC5
CC1
CC30
CC151
Semi Pathogen
Non Pathogen

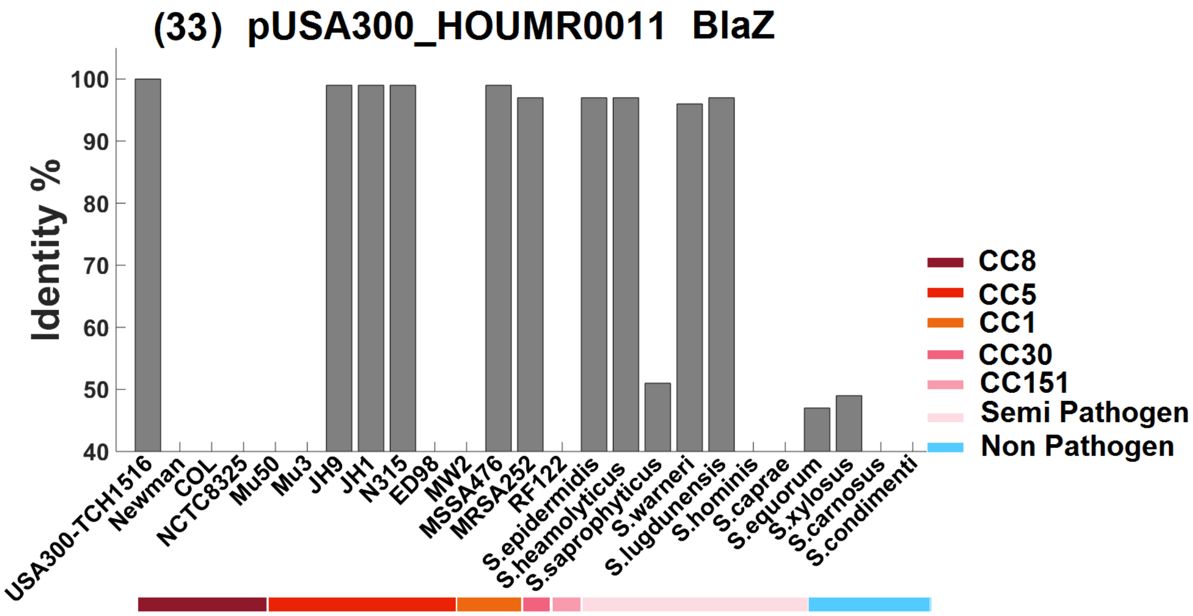
(33) pUSA300_HOUMR0011 BlaZ
Identity %
100
90
80
70
60
50
40
USA300-TCH1516
Newman
COL
NCTC8325
Mu50
Mu3
JH9
JH1
N315
ED98
MW2
MSSA476
MRSA252
RF122
S.epidermidis
S.heamolyticus
S.saprophyticus
S.warneri
S.lugdunensis
S.hominis
S.caprae
S.equorum
S.xylosus
S.carnosus
S.condimenti
CC8
CC5
CC1
CC30
CC151
Semi Pathogen
Non Pathogen

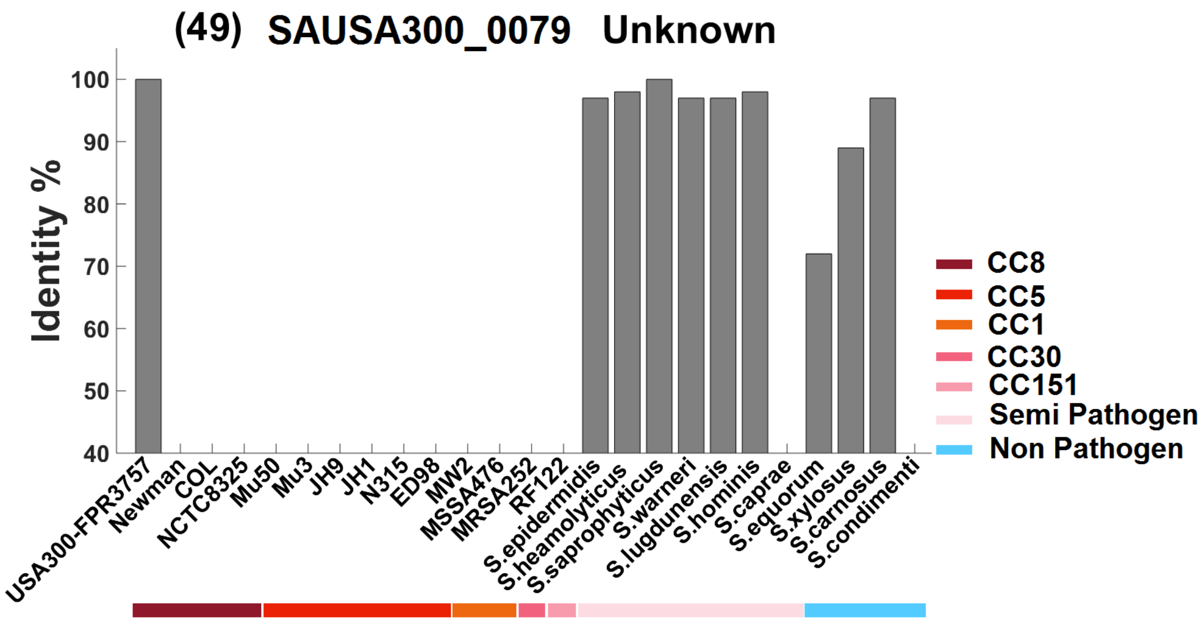
(49) SAUSA300_0079 Unknown
Identity %
100
90
80
70
60
50
40
USA300-FPR3757
Newman
COL
NCTC8325
Mu50
Mu3
JH9
JH1
N315
ED98
MW2
MSSA476
MRSA252
RF122
S.epidermidis
S.heamolyticus
S.saprophyticus
S.warneri
S.lugdunensis
S.hominis
S.caprae
S.equorum
S.xylosus
S.carnosus
S.condimenti
CC8
CC5
CC1
CC30
CC151
Semi Pathogen
Non Pathogen

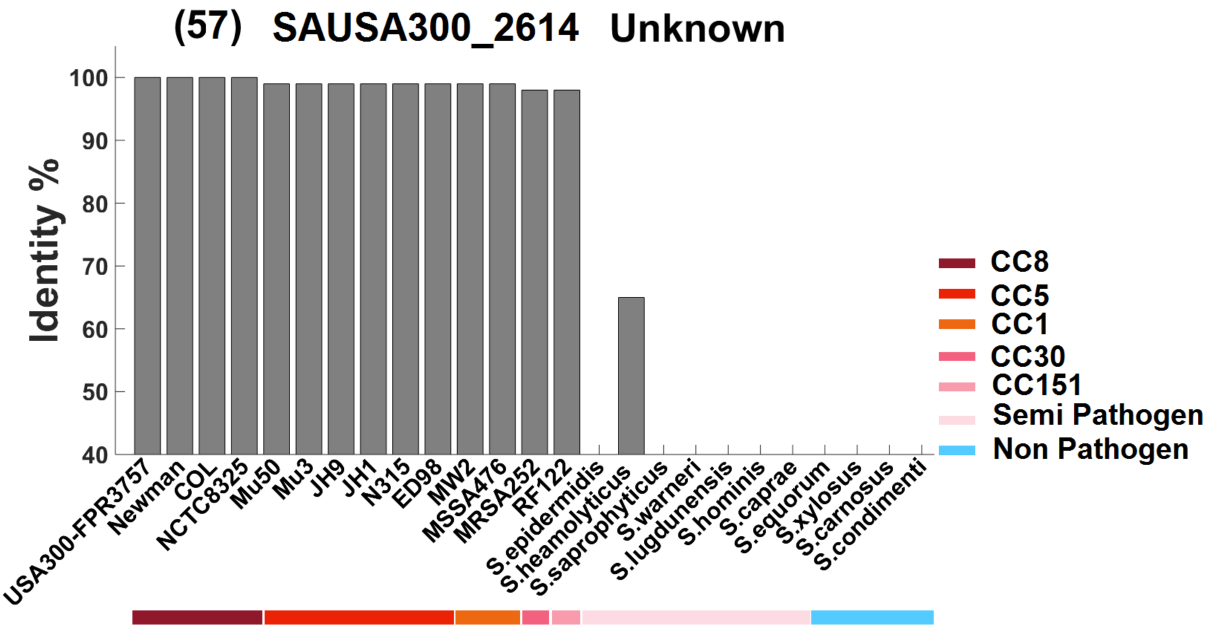
(57) SAUSA300_2614 Unknown
Identity %
100
90
80
70
60
50
40
USA300-FPR3757
Newman
COL
NCTC8325
Mu50
Mu3
JH9
JH1
N315
ED98
MW2
MSSA476
MRSA252
RF122
S.epidermidis
S.heamolyticus
S.saprophyticus
S.warneri
S.lugdunensis
S.hominis
S.caprae
S.equorum
S.xylosus
S.carnosus
S.condimenti
CC8
CC5
CC1
CC30
CC151
Semi Pathogen
Non Pathogen

(B)

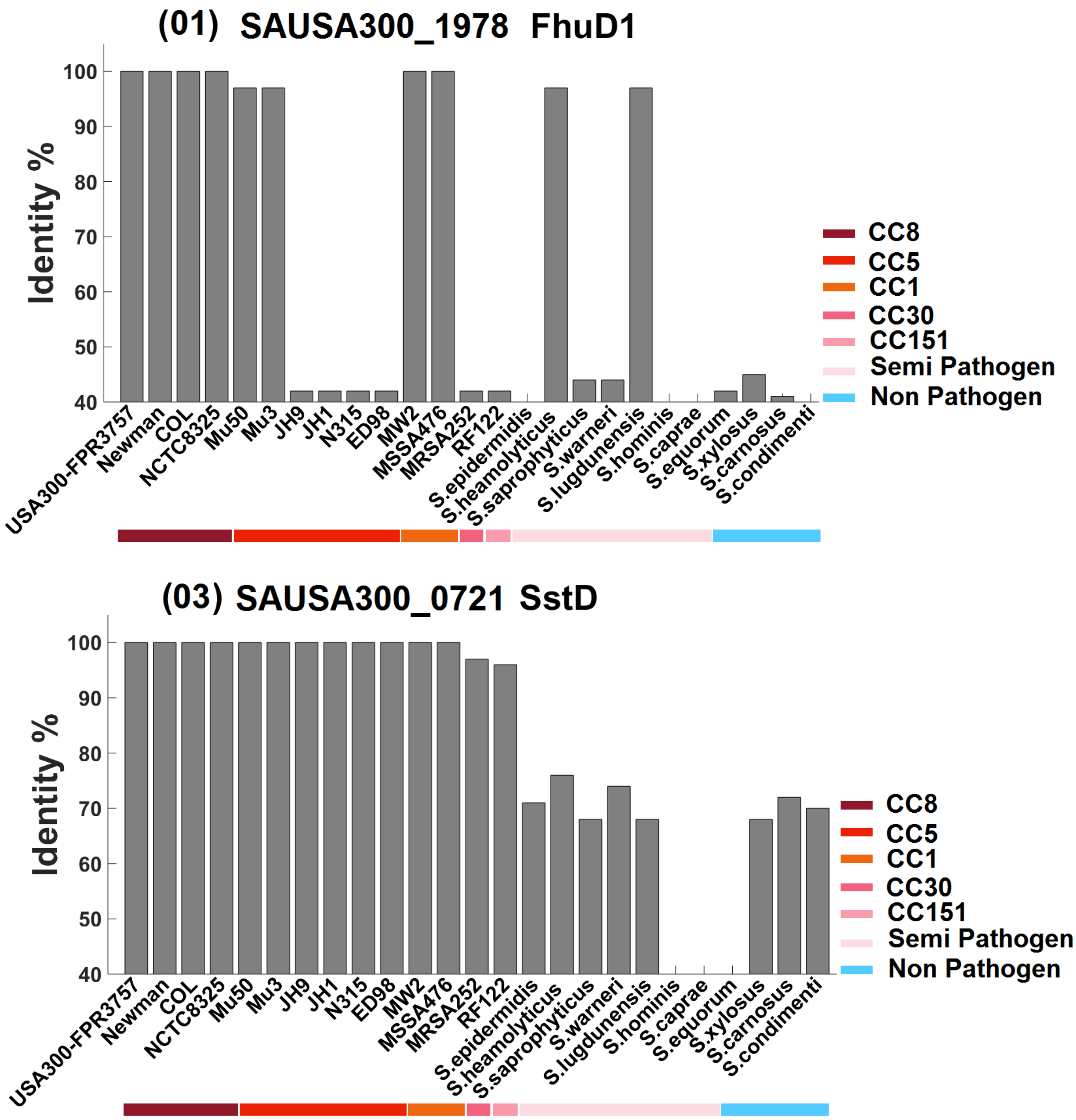

(01) SAUSA300_1978 FhuD1
Identity %
100
90
80
70
60
50
40
USA300-FPR3757
Newman
COL
NCTC8325
Mu50
Mu3
JH9
JH1
N315
ED98
MW2
MSSA476
MRSA252
RF122
S.epidermidis
S.heamolyticus
S.saprophyticus
S.warneri
S.lugdunensis
S.hominis
S.caprae
S.equorum
S.xylosus
S.carnosus
S.condimenti
CC8
CC5
CC1
CC30
CC151
Semi Pathogen
Non Pathogen
(03) SAUSA300_0721 SstD

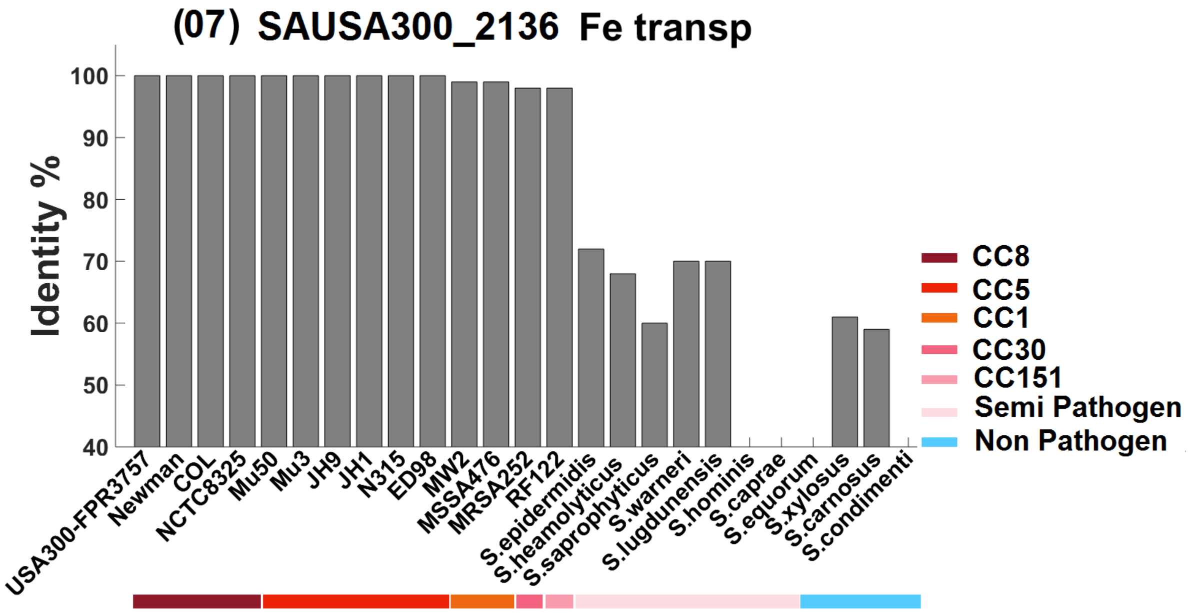
(07) SAUSA300_2136 Fe transp
Identity %
100
90
80
70
60
50
40
USA300-FPR3757
Newman
COL
NCTC8325
Mu50
Mu3
JH9
JH1
N315
ED98
MW2
MSSA476
MRSA252
RF122
S.epidermidis
S.heamolyticus
S.saprophyticus
S.warneri
S.lugdunensis
S.hominis
S.caprae
S.equorum
S.xylosus
S.carnosus
S.condimenti
CC8
CC5
CC1
CC30
CC151
Semi Pathogen
Non Pathogen

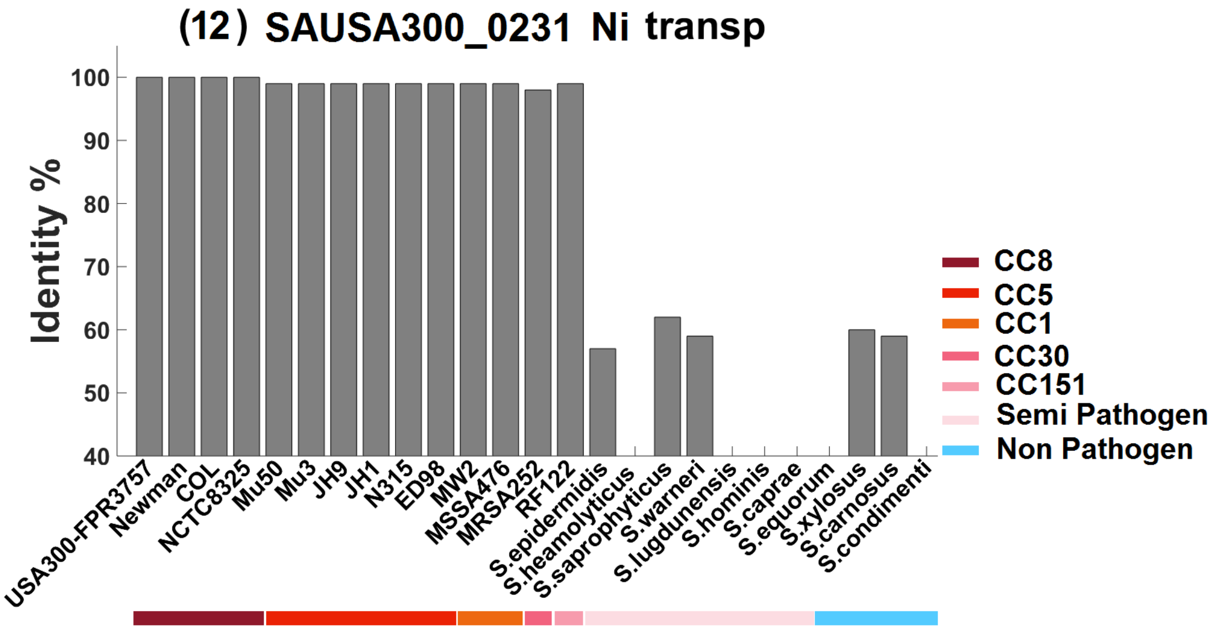
(12) SAUSA300_0231 Ni transp
Identity %
100
90
80
70
60
50
40
USA300-FPR3757
Newman
COL
NCTC8325
Mu50
Mu3
JH9
JH1
N315
ED98
MW2
MSSA476
MRSA252
RF122
S.epidermidis
S.heamolyticus
S.saprophyticus
S.warneri
S.lugdunensis
S.hominis
S.caprae
S.equorum
S.xylosus
S.carnosus
S.condimenti
CC8
CC5
CC1
CC30
CC151
Semi Pathogen
Non Pathogen

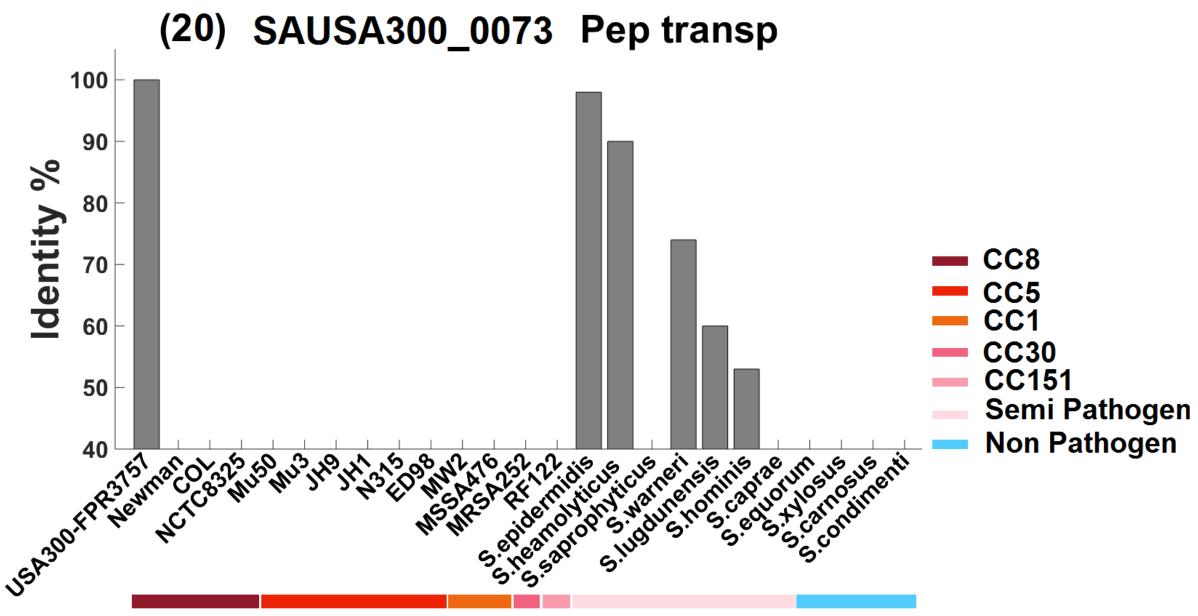
(20) SAUSA300_0073 Pep transp
Identity %
100
90
80
70
60
50
40
USA300-FPR3757
Newman
COL
NCTC8325
Mu50
Mu3
JH9
JH1
N315
ED98
MW2
MSSA476
MRSA252
RF122
S.epidermidis
S.heamolyticus
S.saprophyticus
S.warneri
S.lugdunensis
S.hominis
S.caprae
S.equorum
S.xylosus
S.carnosus
S.condimenti
CC8
CC5
CC1
CC30
CC151
Semi Pathogen
Non Pathogen

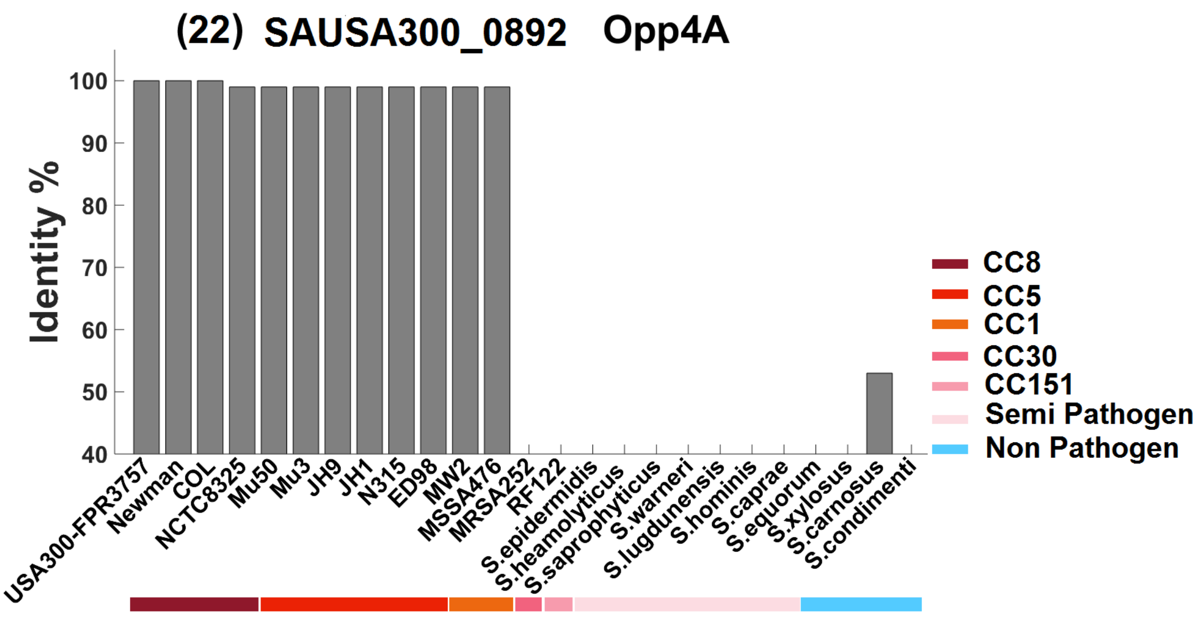
(22) SAUSA300_0892 Opp4A
Identity %
100
90
80
70
60
50
40
USA300-FPR3757
Newman
COL
NCTC8325
Mu50
Mu3
JH9
JH1
N315
ED98
MW2
MSSA476
MRSA252
RF122
S.epidermidis
S.heamolyticus
S.saprophyticus
S.warneri
S.lugdunensis
S.hominis
S.caprae
S.equorum
S.xylosus
S.carnosus
S.condimenti
CC8
CC5
CC1
CC30
CC151
Semi Pathogen
Non Pathogen

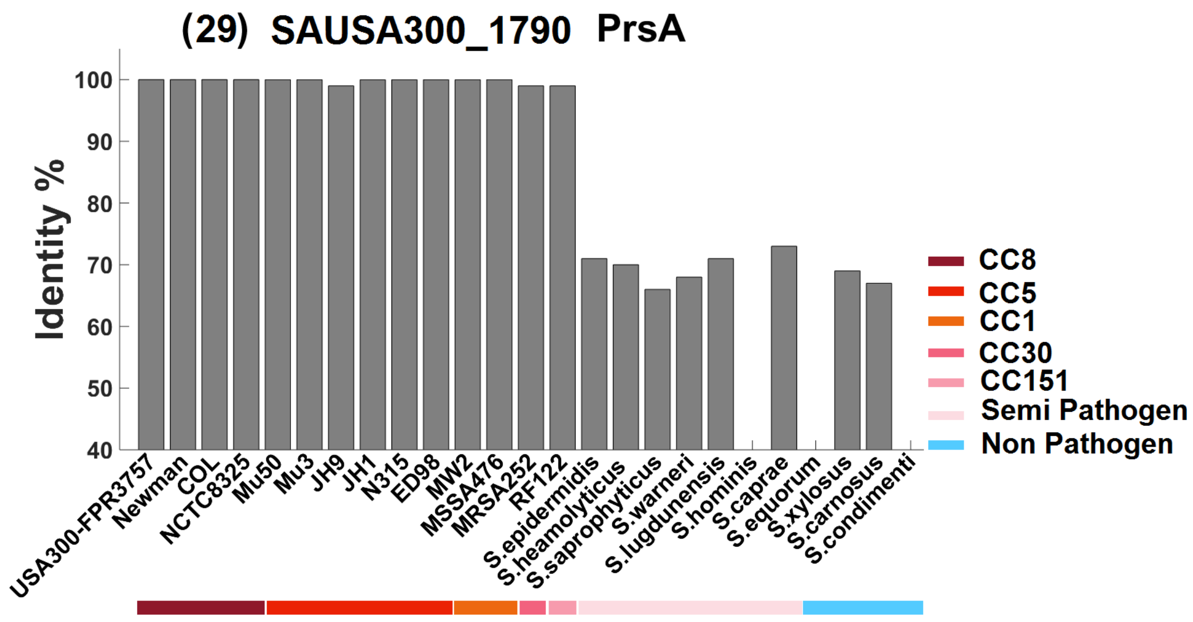
(29) SAUSA300_1790 PrsA
Identity %
100
90
80
70
60
50
40
USA300-FPR3757
Newman
COL
NCTC8325
Mu50
Mu3
JH9
JH1
N315
ED98
MW2
MSSA476
MRSA252
RF122
S.epidermidis
S.heamolyticus
S.saprophyticus
S.warneri
S.lugdunensis
S.hominis
S.caprae
S.equorum
S.xylosus
S.carnosus
S.condimenti
CC8
CC5
CC1
CC30
CC151
Semi Pathogen
Non Pathogen

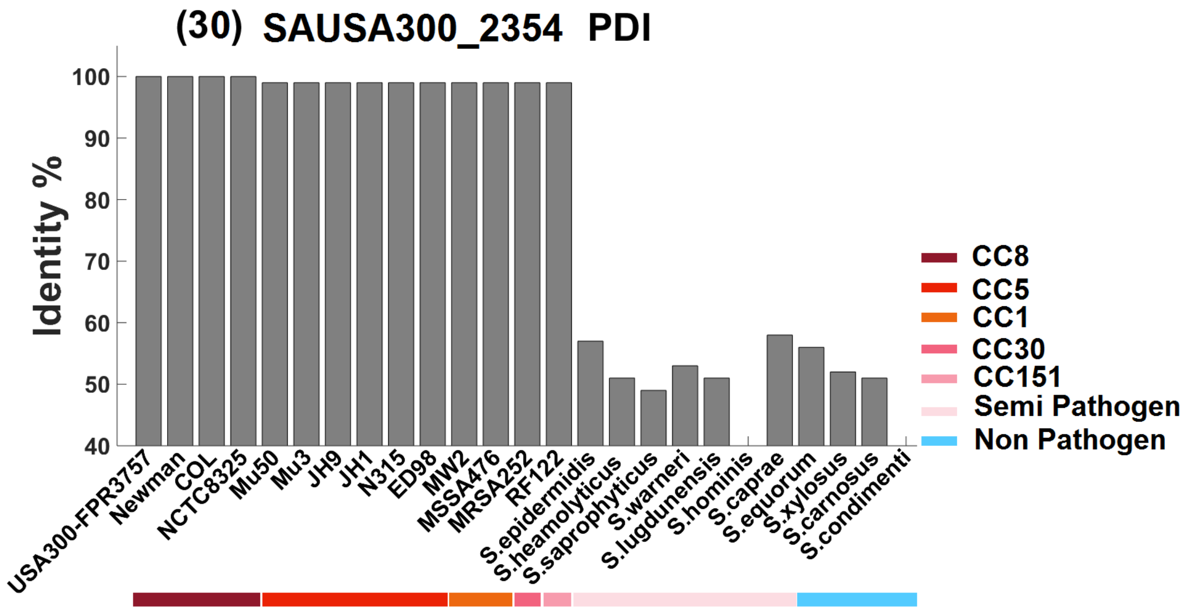
(30) SAUSA300_2354 PDI
Identity %
100
90
80
70
60
50
40
USA300-FPR3757
Newman
COL
NCTC8325
Mu50
Mu3
JH9
JH1
N315
ED98
MW2
MSSA476
MRSA252
RF122
S.epidermidis
S.heamolyticus
S.saprophyticus
S.warneri
S.lugdunensis
S.hominis
S.caprae
S.equorum
S.xylosus
S.carnosus
S.condimenti
CC8
CC5
CC1
CC30
CC151
Semi Pathogen
Non Pathogen

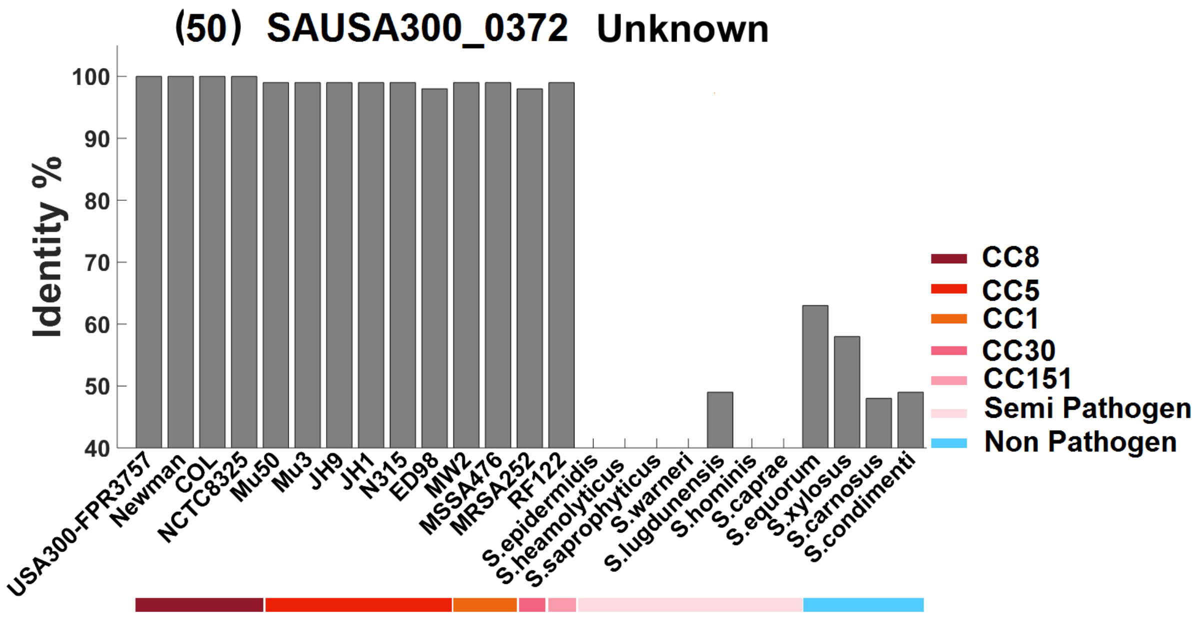
(50) SAUSA300_0372 Unknown
Identity %
100
90
80
70
60
50
40
USA300-FPR3757
Newman
COL
NCTC8325
Mu50
Mu3
JH9
JH1
N315
ED98
MW2
MSSA476
MRSA252
RF122
S.epidermidis
S.heamolyticus
S.saprophyticus
S.warneri
S.lugdunensis
S.hominis
S.caprae
S.equorum
S.xylosus
S.carnosus
S.condimenti
CC8
CC5
CC1
CC30
CC151
Semi Pathogen
Non Pathogen

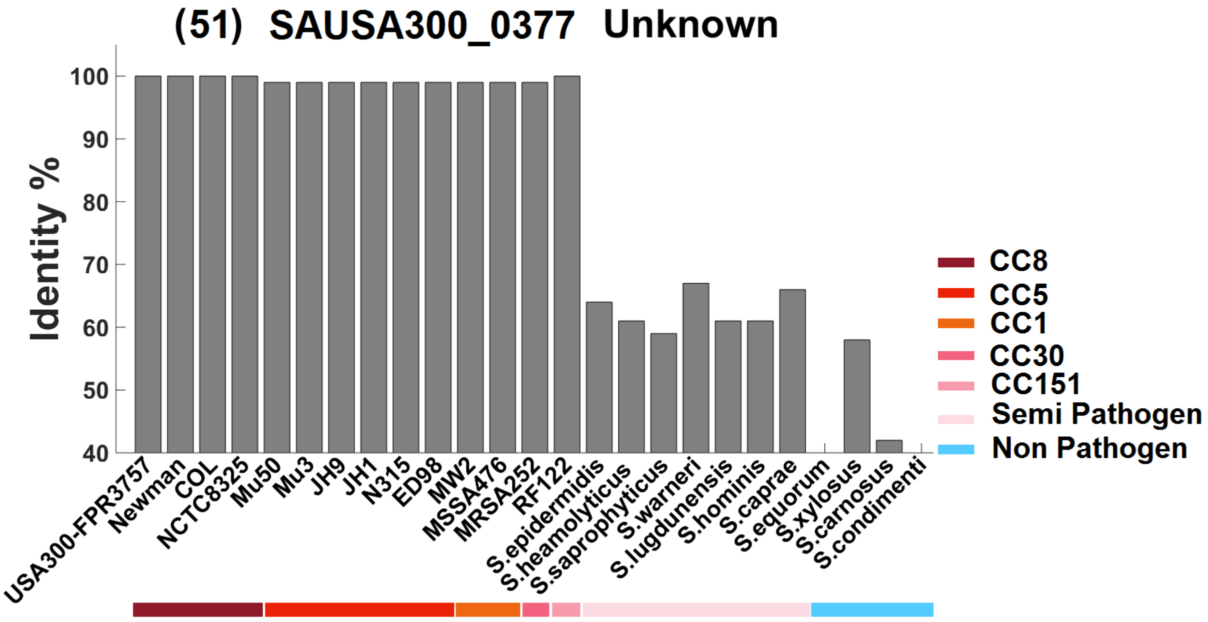
(51) SAUSA300_0377 Unknown
Identity %
100
90
80
70
60
50
40
USA300-FPR3757
Newman
COL
NCTC8325
Mu50
Mu3
JH9
JH1
N315
ED98
MW2
MSSA476
MRSA252
RF122
S.epidermidis
S.heamolyticus
S.saprophyticus
S.warneri
S.lugdunensis
S.hominis
S.caprae
S.equorum
S.xylosus
S.carnosus
S.condimenti
CC8
CC5
CC1
CC30
CC151
Semi Pathogen
Non Pathogen

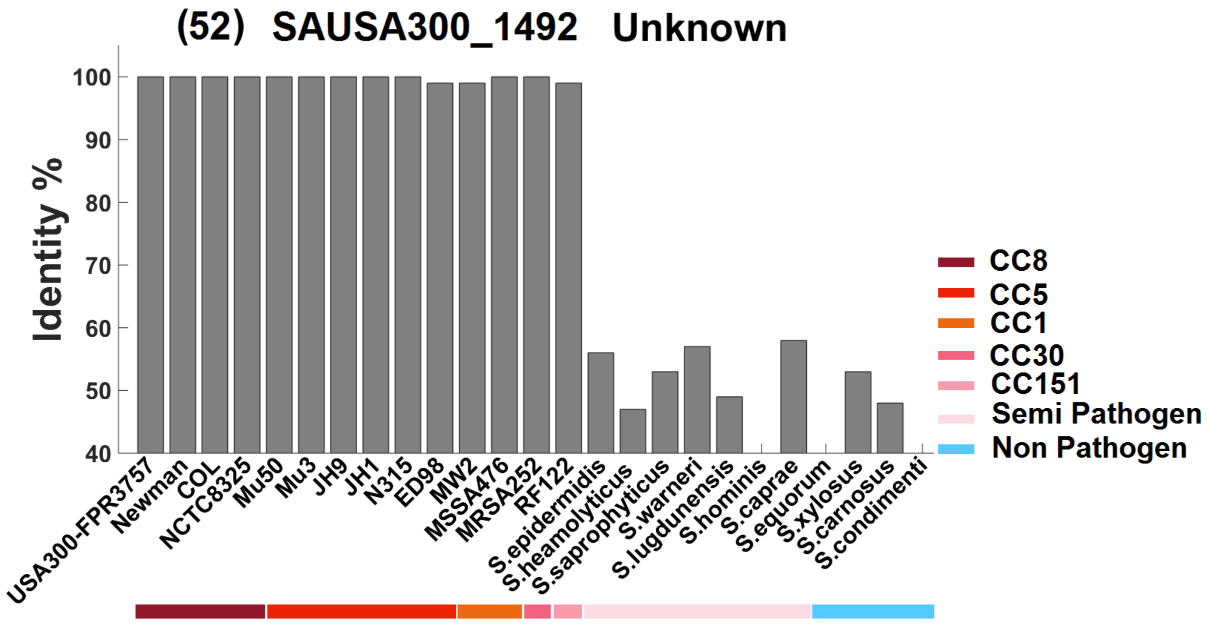
(52) SAUSA300_1492 Unknown
Identity %
100
90
80
70
60
50
40
USA300-FPR3757
Newman
COL
NCTC8325
Mu50
Mu3
JH9
JH1
N315
ED98
MW2
MSSA476
MRSA252
RF122
S.epidermidis
S.heamolyticus
S.saprophyticus
S.warneri
S.lugdunensis
S.hominis
S.caprae
S.equorum
S.xylosus
S.carnosus
S.condimenti
CC8
CC5
CC1
CC30
CC151
Semi Pathogen
Non Pathogen

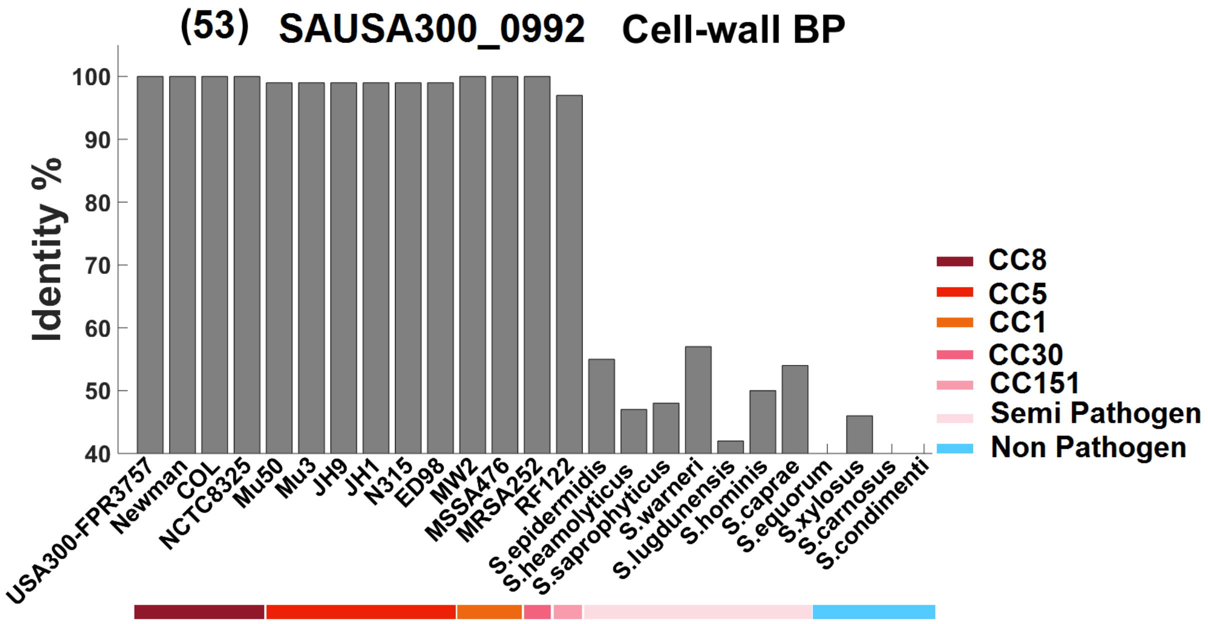
(53) SAUSA300_0992 Cell-wall BP
Identity %
100
90
80
70
60
50
40
USA300-FPR3757
Newman
COL
NCTC8325
Mu50
Mu3
JH9
JH1
N315
ED98
MW2
MSSA476
MRSA252
RF122
S.epidermidis
S.heamolyticus
S.saprophyticus
S.warneri
S.lugdunensis
S.hominis
S.caprae
S.equorum
S.xylosus
S.carnosus
S.condimenti
CC8
CC5
CC1
CC30
CC151
Semi Pathogen
Non Pathogen

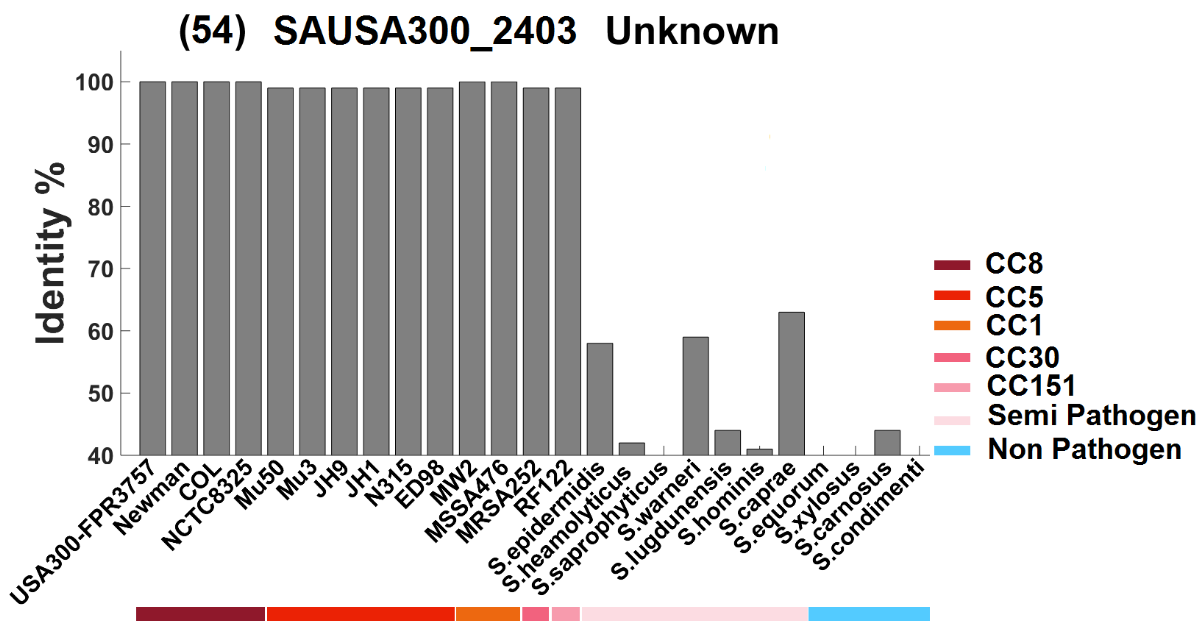
(54) SAUSA300_2403 Unknown
Identity %
100
90
80
70
60
50
40
USA300-FPR3757
Newman
COL
NCTC8325
Mu50
Mu3
JH9
JH1
N315
ED98
MW2
MSSA476
MRSA252
RF122
S.epidermidis
S.heamolyticus
S.saprophyticus
S.warneri
S.lugdunensis
S.hominis
S.caprae
S.equorum
S.xylosus
S.carnosus
S.condimenti
CC8
CC5
CC1
CC30
CC151
Semi Pathogen
Non Pathogen

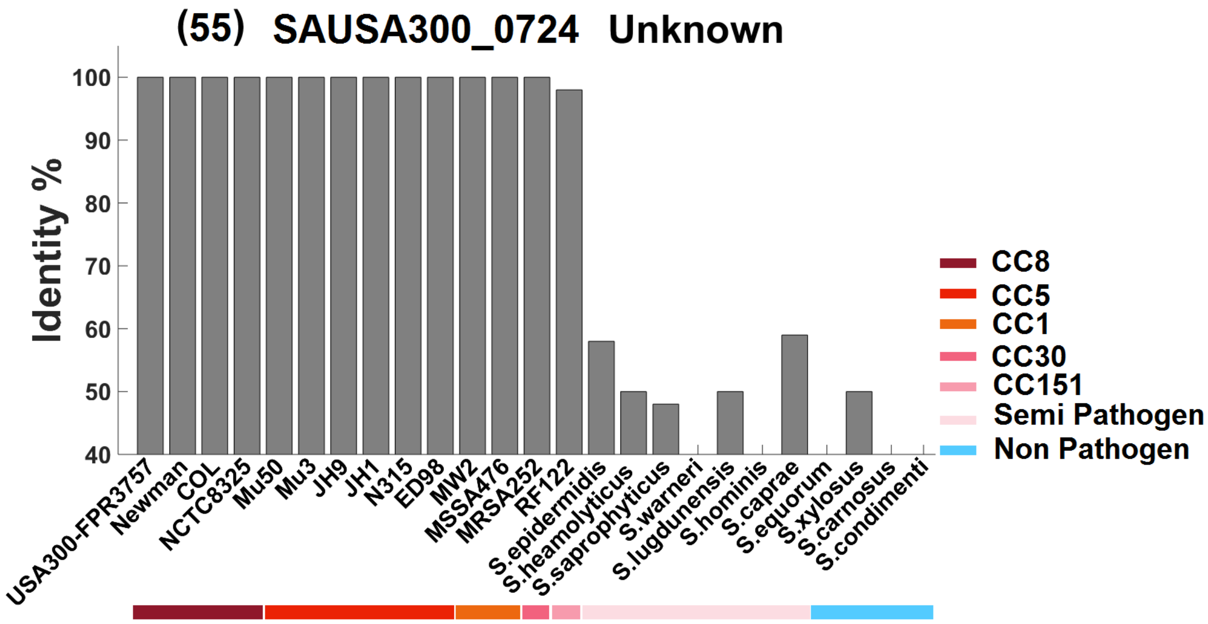
(55) SAUSA300_0724 Unknown
Identity %
100
90
80
70
60
50
40
USA300-FPR3757
Newman
COL
NCTC8325
Mu50
Mu3
JH9
JH1
N315
ED98
MW2
MSSA476
MRSA252
RF122
S.epidermidis
S.heamolyticus
S.saprophyticus
S.warneri
S.lugdunensis
S.hominis
S.caprae
S.equorum
S.xylosus
S.carnosus
S.condimenti
CC8
CC5
CC1
CC30
CC151
Semi Pathogen
Non Pathogen

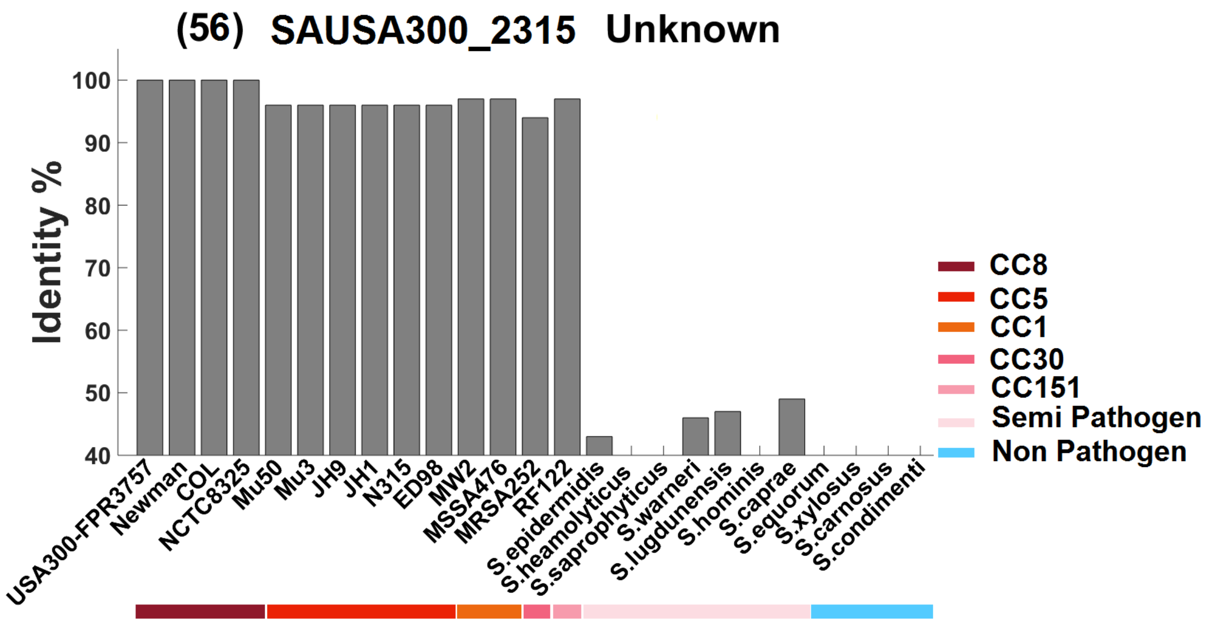
(56) SAUSA300_2315 Unknown
Identity %
100
90
80
70
60
50
40
USA300-FPR3757
Newman
COL
NCTC8325
Mu50
Mu3
JH9
JH1
N315
ED98
MW2
MSSA476
MRSA252
RF122
S.epidermidis
S.heamolyticus
S.saprophyticus
S.warneri
S.lugdunensis
S.hominis
S.caprae
S.equorum
S.xylosus
S.carnosus
S.condimenti
CC8
CC5
CC1
CC30
CC151
Semi Pathogen
Non Pathogen

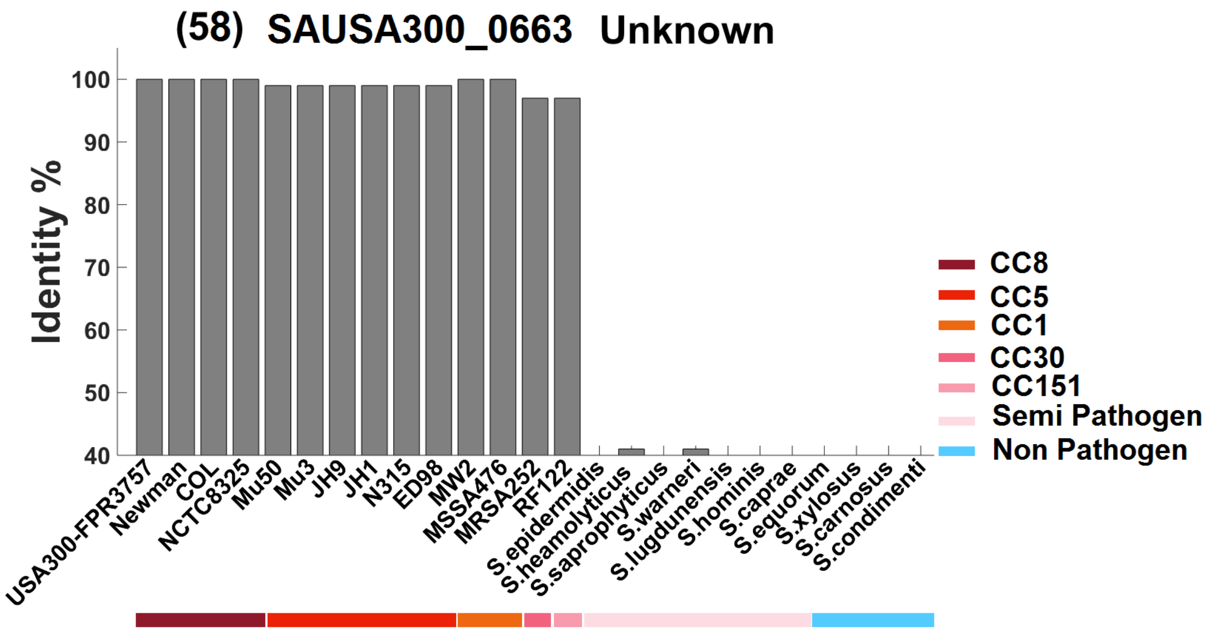
(58) SAUSA300_0663 Unknown
Identity %
100
90
80
70
60
50
40
USA300-FPR3757
Newman
COL
NCTC8325
Mu50
Mu3
JH9
JH1
N315
ED98
MW2
MSSA476
MRSA252
RF122
S.epidermidis
S.heamolyticus
S.saprophyticus
S.warneri
S.lugdunensis
S.hominis
S.caprae
S.equorum
S.xylosus
S.carnosus
S.condimenti
CC8
CC5
CC1
CC30
CC151
Semi Pathogen
Non Pathogen

(C)

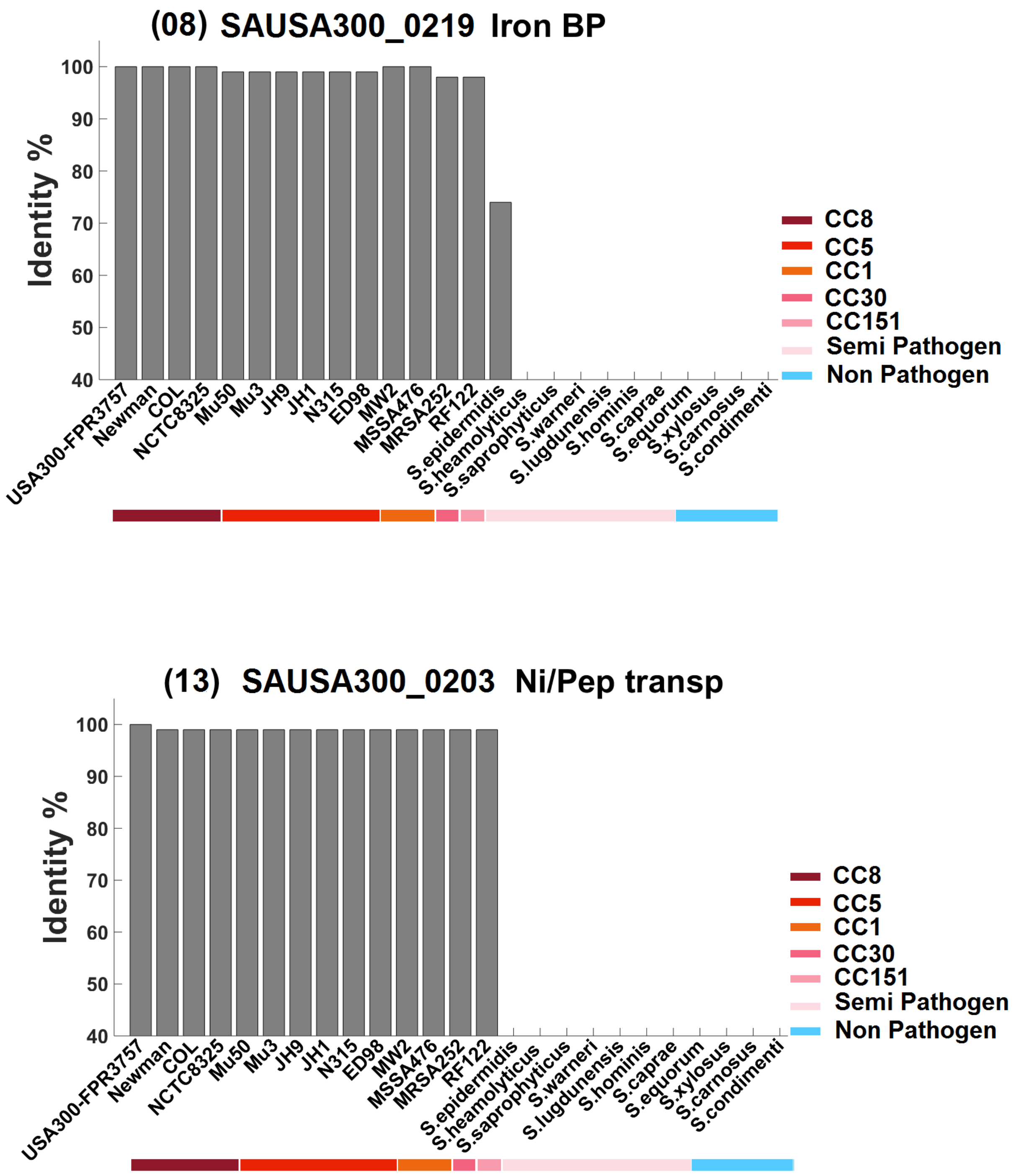

(08) SAUSA300_0219 Iron BP
Identity %
100
90
80
70
60
50
40
USA300-FPR3757
Newman
COL
NCTC8325
Mu50
Mu3
JH9
JH1
N315
ED98
MW2
MSSA476
MRSA252
RF122
S.epidermidis
S.heamolyticus
S.saprophyticus
S.warneri
S.lugdunensis
S.hominis
S.caprae
S.equorum
S.xylosus
S.carnosus
S.condimenti
CC8
CC5
CC1
CC30
CC151
Semi Pathogen
Non Pathogen
(13) SAUSA300_0203 Ni/Pep transp
Identity %
100
90
80
70
60
50
40
USA300-FPR3757
Newman
COL
NCTC8325
Mu50
Mu3
JH9
JH1
N315
ED98
MW2
MSSA476
MRSA252
RF122
S.epidermidis
S.heamolyticus
S.saprophyticus
S.warneri
S.lugdunensis
S.hominis
S.caprae
S.equorum
S.xylosus
S.carnosus
S.condimenti
CC8
CC5
CC1
CC30
CC151
Semi Pathogen
Non Pathogen

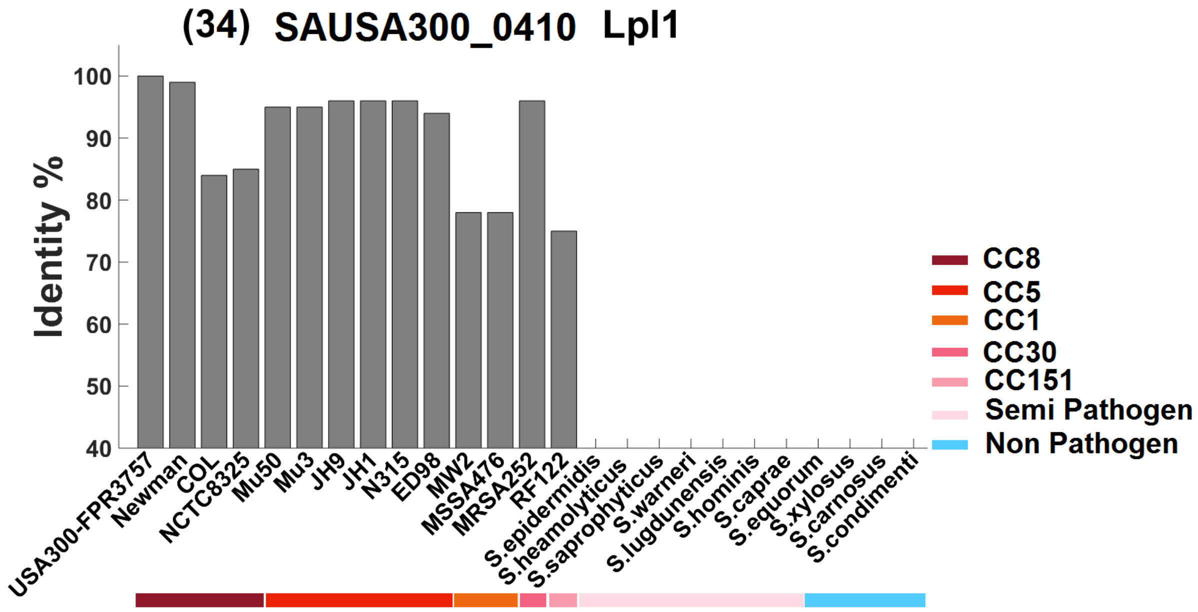
(34) SAUSA300_0410 Lpl1
Identity %
100
90
80
70
60
50
40
USA300-FPR3757
Newman
COL
NCTC8325
Mu50
Mu3
JH9
JH1
N315
ED98
MW2
MSSA476
MRSA252
RF122
S.epidermidis
S.heamolyticus
S.saprophyticus
S.warneri
S.lugdunensis
S.hominis
S.caprae
S.equorum
S.xylosus
S.carnosus
S.condimenti
CC8
CC5
CC1
CC30
CC151
Semi Pathogen
Non Pathogen

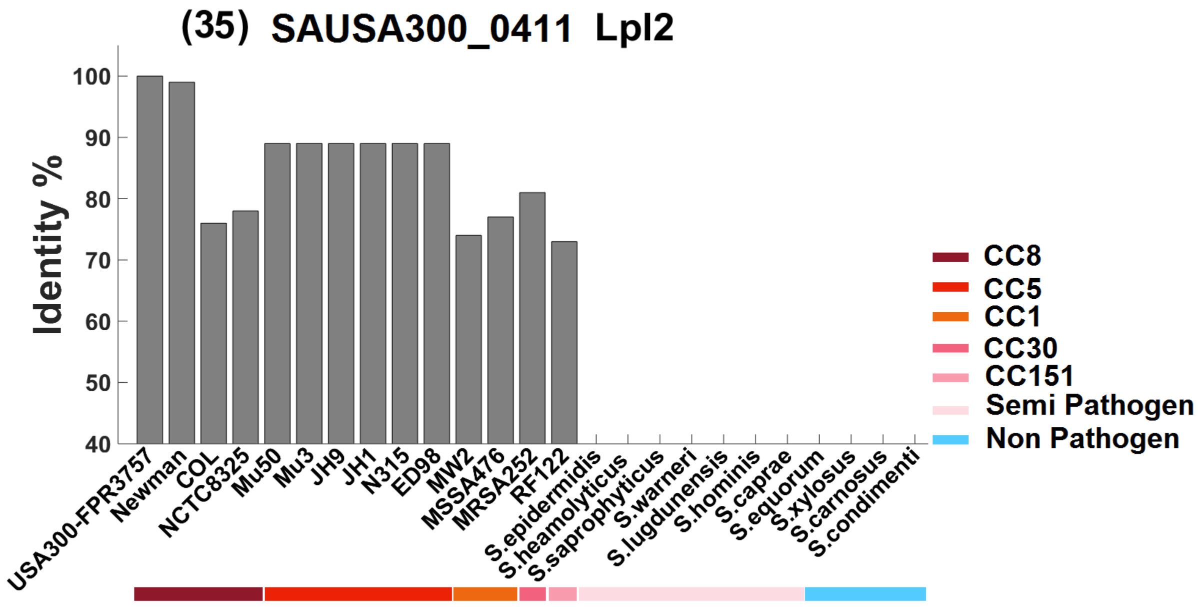
(35) SAUSA300_0411 Lpl2
Identity %
100
90
80
70
60
50
40
USA300-FPR3757
Newman
COL
NCTC8325
Mu50
Mu3
JH9
JH1
N315
ED98
MW2
MSSA476
MRSA252
RF122
S.epidermidis
S.heamolyticus
S.saprophyticus
S.warneri
S.lugdunensis
S.hominis
S.caprae
S.equorum
S.xylosus
S.carnosus
S.condimenti
CC8
CC5
CC1
CC30
CC151
Semi Pathogen
Non Pathogen

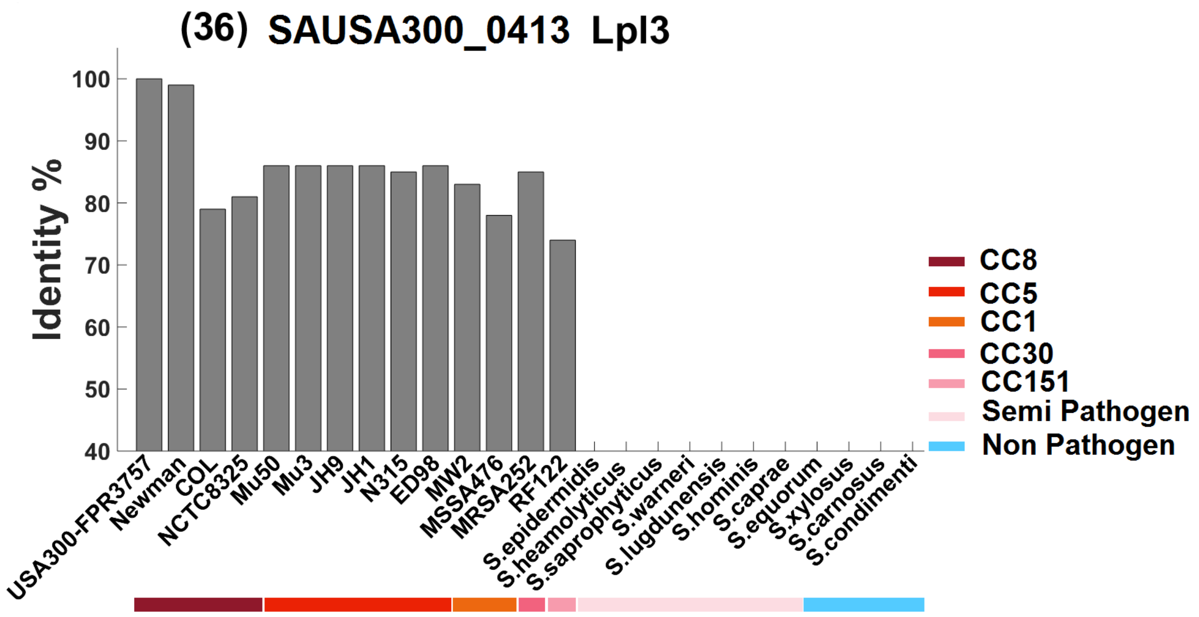
(36) SAUSA300_0413 Lpl3
Identity %
100
90
80
70
60
50
40
USA300-FPR3757
Newman
COL
NCTC8325
Mu50
Mu3
JH9
JH1
N315
ED98
MW2
MSSA476
MRSA252
RF122
S.epidermidis
S.heamolyticus
S.saprophyticus
S.warneri
S.lugdunensis
S.hominis
S.caprae
S.equorum
S.xylosus
S.carnosus
S.condimenti
CC8
CC5
CC1
CC30
CC151
Semi Pathogen
Non Pathogen

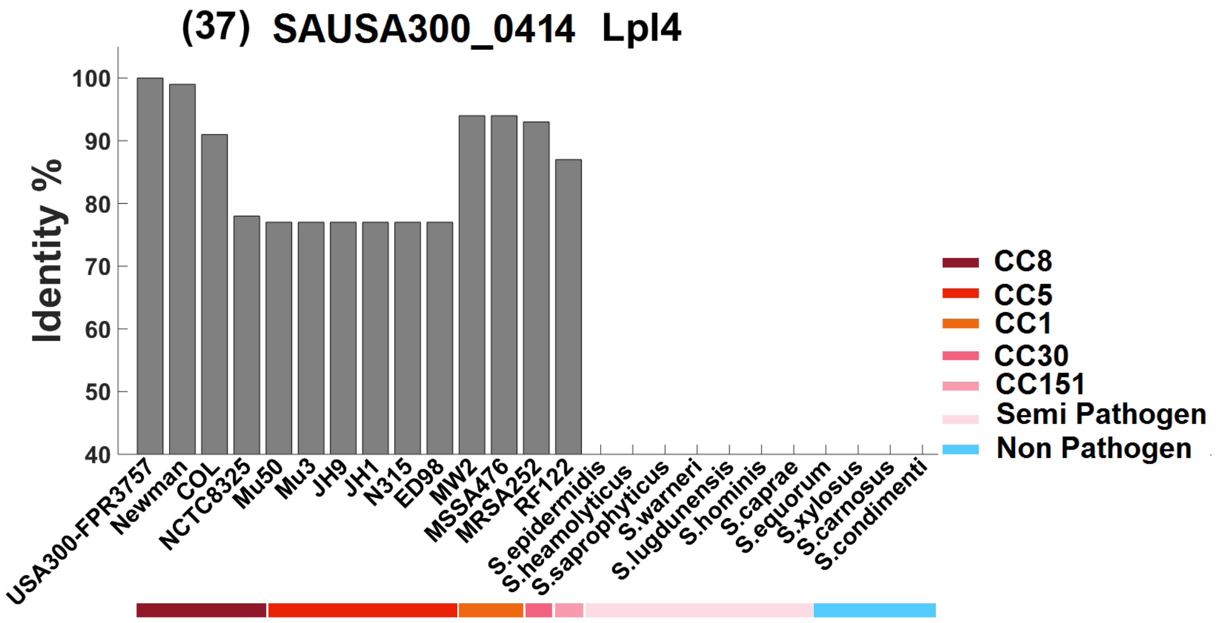
(37) SAUSA300_0414 Lpl4
Identity %
100
90
80
70
60
50
40
USA300-FPR3757
Newman
COL
NCTC8325
Mu50
Mu3
JH9
JH1
N315
ED98
MW2
MSSA476
MRSA252
RF122
S.epidermidis
S.heamolyticus
S.saprophyticus
S.warneri
S.lugdunensis
S.hominis
S.caprae
S.equorum
S.xylosus
S.carnosus
S.condimenti
CC8
CC5
CC1
CC30
CC151
Semi Pathogen
Non Pathogen

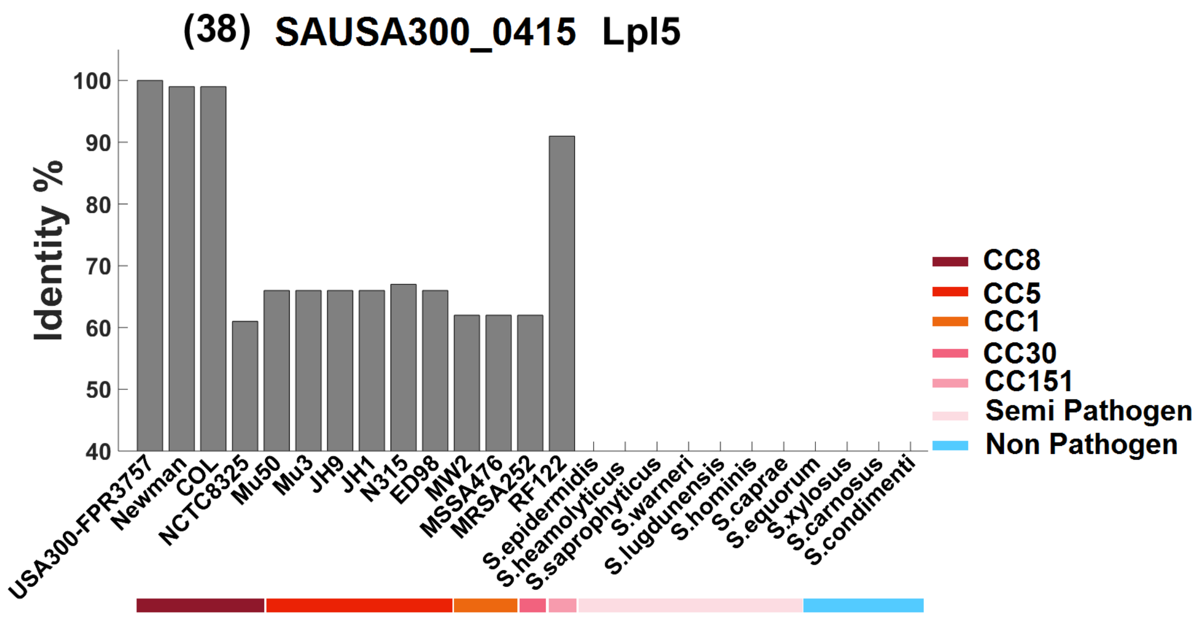

(38) SAUSA300_0415 Lpl5
Identity %
100
90
80
70
60
50
40
USA300-FPR3757
Newman
COL
NCTC8325
Mu50
Mu3
JH9
JH1
N315
ED98
MW2
MSSA476
MRSA252
RF122
S.epidermidis
S.heamolyticus
S.saprophyticus
S.warneri
S.lugdunensis
S.hominis
S.caprae
S.equorum
S.xylosus
S.carnosus
S.condimenti
CC8
CC5
CC1
CC30
CC151
Semi Pathogen
Non Pathogen

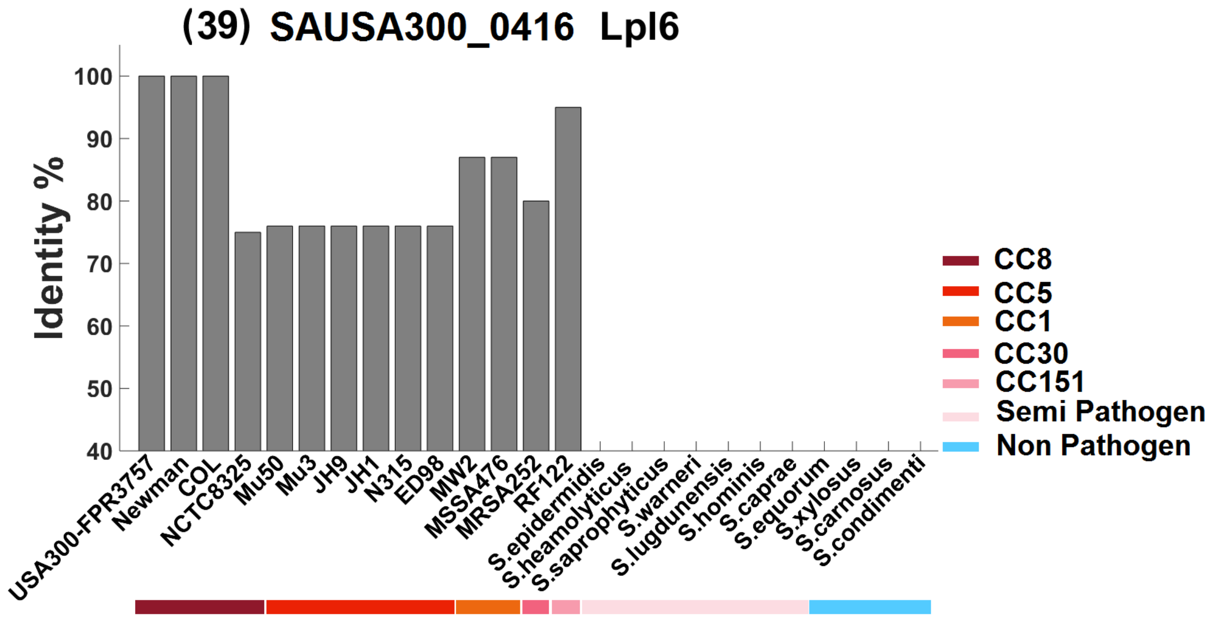

(39) SAUSA300_0416 Lpl6
Identity %
100
90
80
70
60
50
40
USA300-FPR3757
Newman
COL
NCTC8325
Mu50
Mu3
JH9
JH1
N315
ED98
MW2
MSSA476
MRSA252
RF122
S.epidermidis
S.heamolyticus
S.saprophyticus
S.warneri
S.lugdunensis
S.hominis
S.caprae
S.equorum
S.xylosus
S.carnosus
S.condimenti
CC8
CC5
CC1
CC30
CC151
Semi Pathogen
Non Pathogen

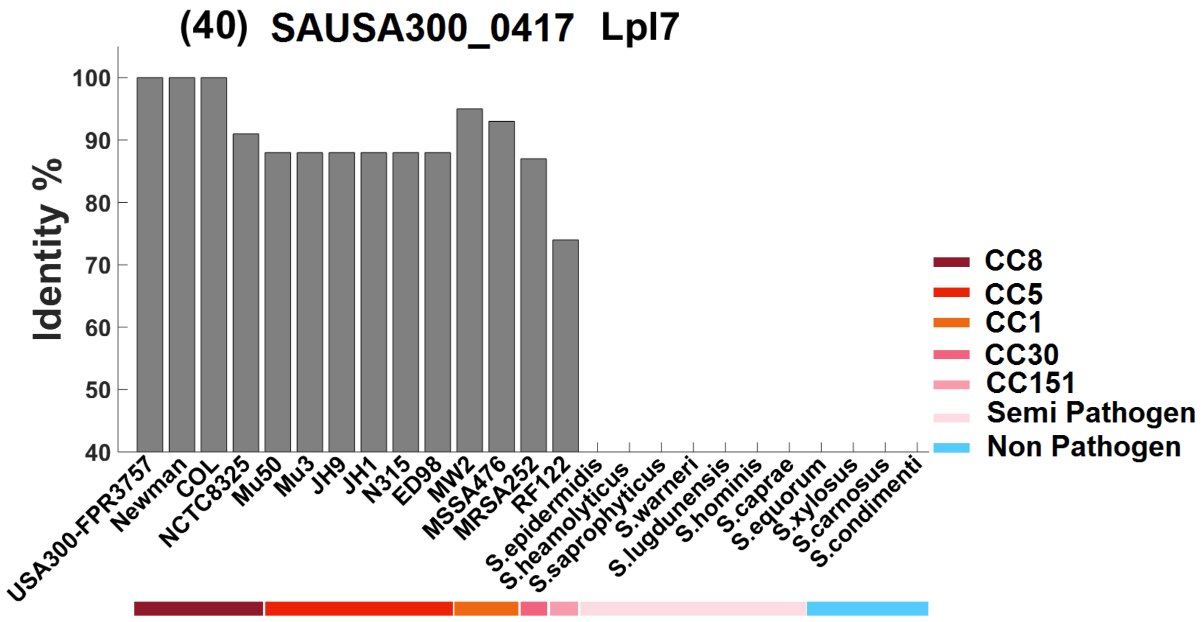

(40) SAUSA300_0417 Lpl7
Identity %
100
90
80
70
60
50
40
USA300-FPR3757
Newman
COL
NCTC8325
Mu50
Mu3
JH9
JH1
N315
ED98
MW2
MSSA476
MRSA252
RF122
S.epidermidis
S.heamolyticus
S.saprophyticus
S.warneri
S.lugdunensis
S.hominis
S.caprae
S.equorum
S.xylosus
S.carnosus
S.condimenti
CC8
CC5
CC1
CC30
CC151
Semi Pathogen
Non Pathogen

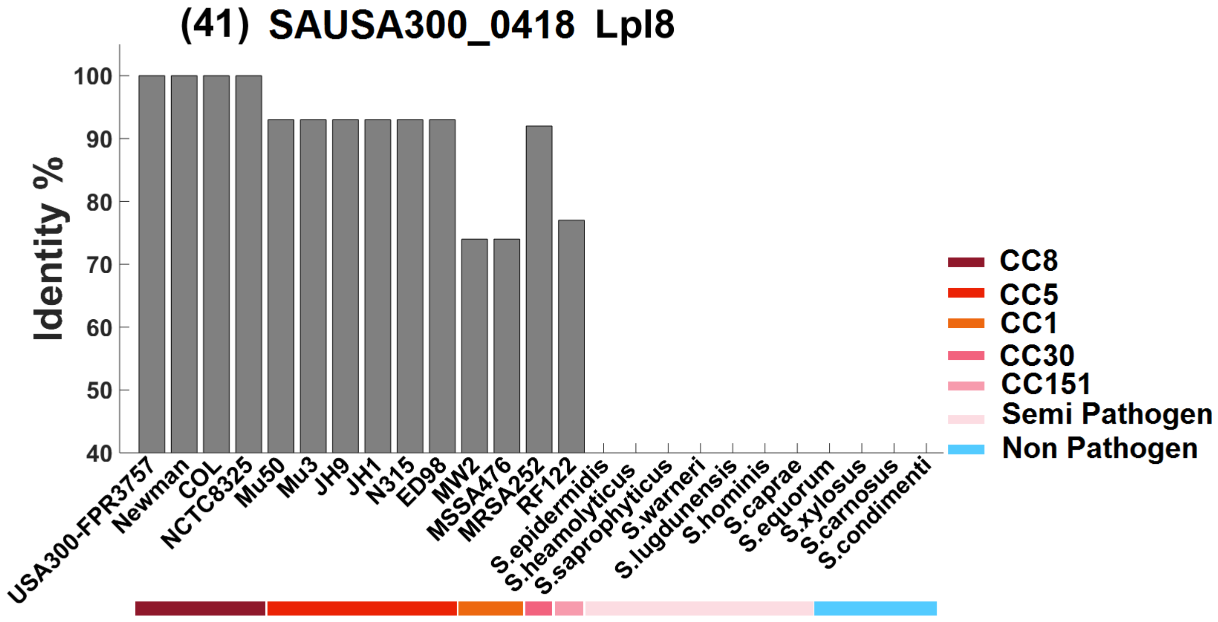

(41) SAUSA300_0418 Lpl8
Identity %
100
90
80
70
60
50
40
USA300-FPR3757
Newman
COL
NCTC8325
Mu50
Mu3
JH9
JH1
N315
ED98
MW2
MSSA476
MRSA252
RF122
S.epidermidis
S.heamolyticus
S.saprophyticus
S.warneri
S.lugdunensis
S.hominis
S.caprae
S.equorum
S.xylosus
S.carnosus
S.condimenti
CC8
CC5
CC1
CC30
CC151
Semi Pathogen
Non Pathogen

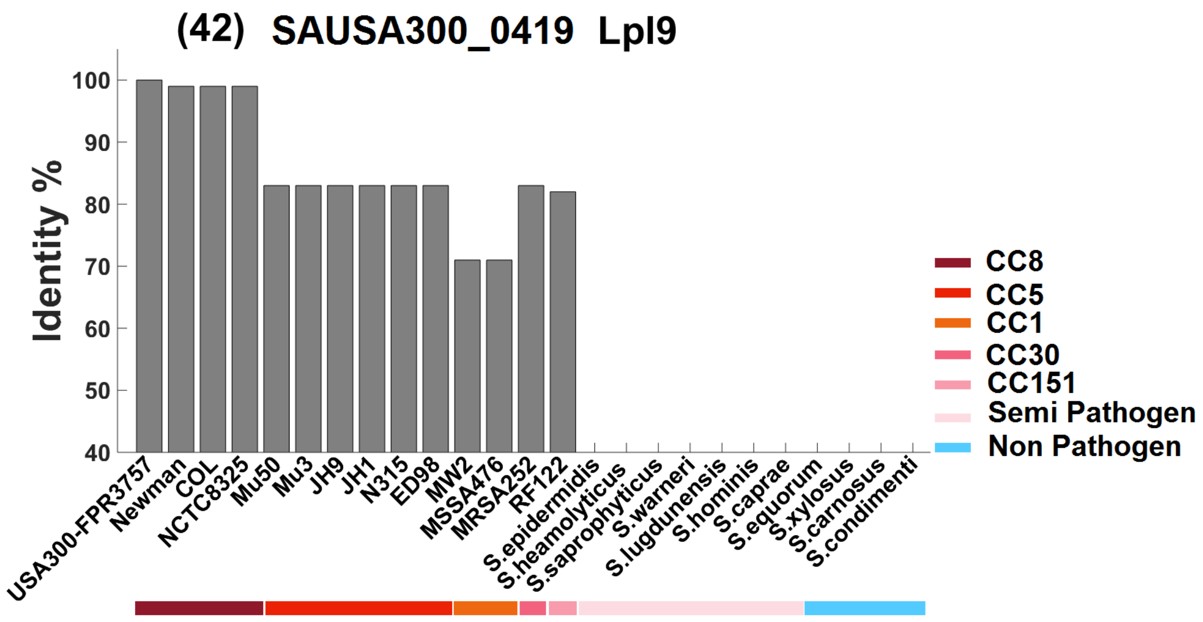
(42) SAUSA300_0419 Lpl9
Identity %
100
90
80
70
60
50
40
USA300-FPR3757
Newman
COL
NCTC8325
Mu50
Mu3
JH9
JH1
N315
ED98
MW2
MSSA476
MRSA252
RF122
S.epidermidis
S.heamolyticus
S.saprophyticus
S.warneri
S.lugdunensis
S.hominis
S.caprae
S.equorum
S.xylosus
S.carnosus
S.condimenti
CC8
CC5
CC1
CC30
CC151
Semi Pathogen
Non Pathogen

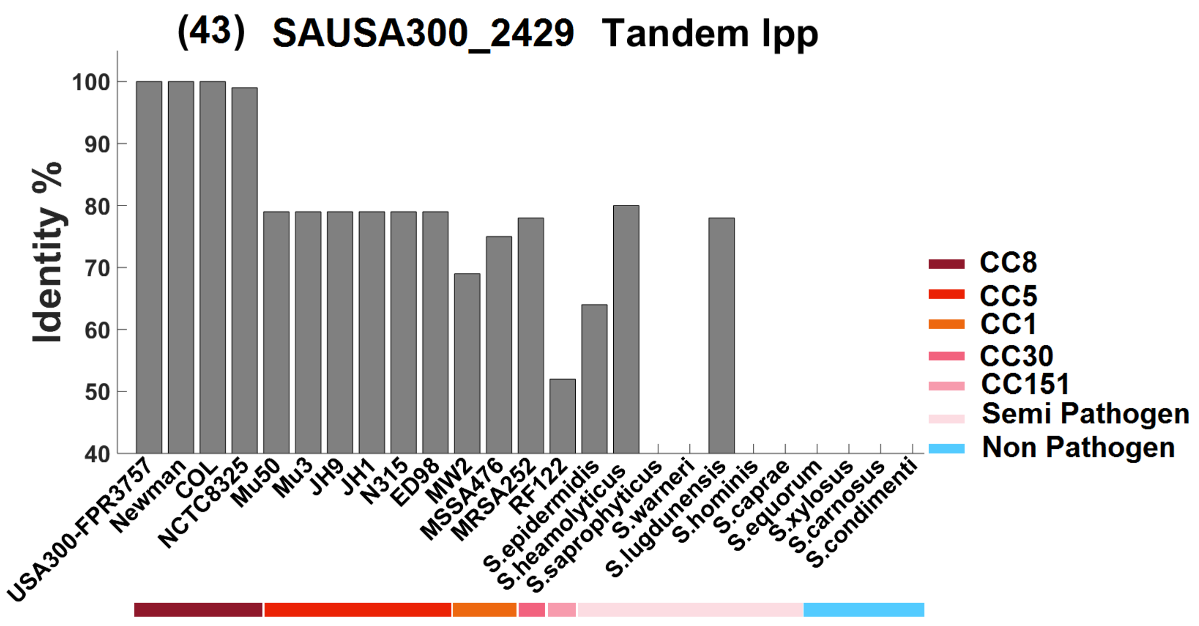
(43) SAUSA300_2429 Tandem lpp
Identity %
100
90
80
70
60
50
40
USA300-FPR3757
Newman
COL
NCTC8325
Mu50
Mu3
JH9
JH1
N315
ED98
MW2
MSSA476
MRSA252
RF122
S.epidermidis
S.heamolyticus
S.saprophyticus
S.warneri
S.lugdunensis
S.hominis
S.caprae
S.equorum
S.xylosus
S.carnosus
S.condimenti
CC8
CC5
CC1
CC30
CC151
Semi Pathogen
Non Pathogen

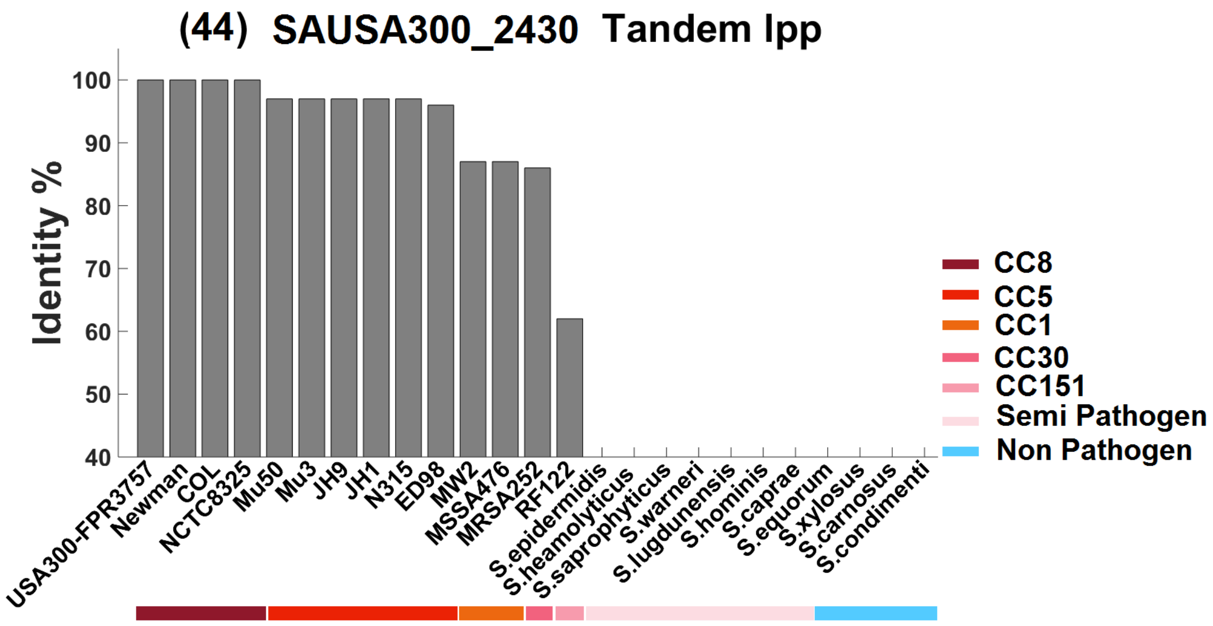
(44) SAUSA300_2430 Tandem lpp
Identity %
100
90
80
70
60
50
40
USA300-FPR3757
Newman
COL
NCTC8325
Mu50
Mu3
JH9
JH1
N315
ED98
MW2
MSSA476
MRSA252
RF122
S.epidermidis
S.heamolyticus
S.saprophyticus
S.warneri
S.lugdunensis
S.hominis
S.caprae
S.equorum
S.xylosus
S.carnosus
S.condimenti
CC8
CC5
CC1
CC30
CC151
Semi Pathogen
Non Pathogen

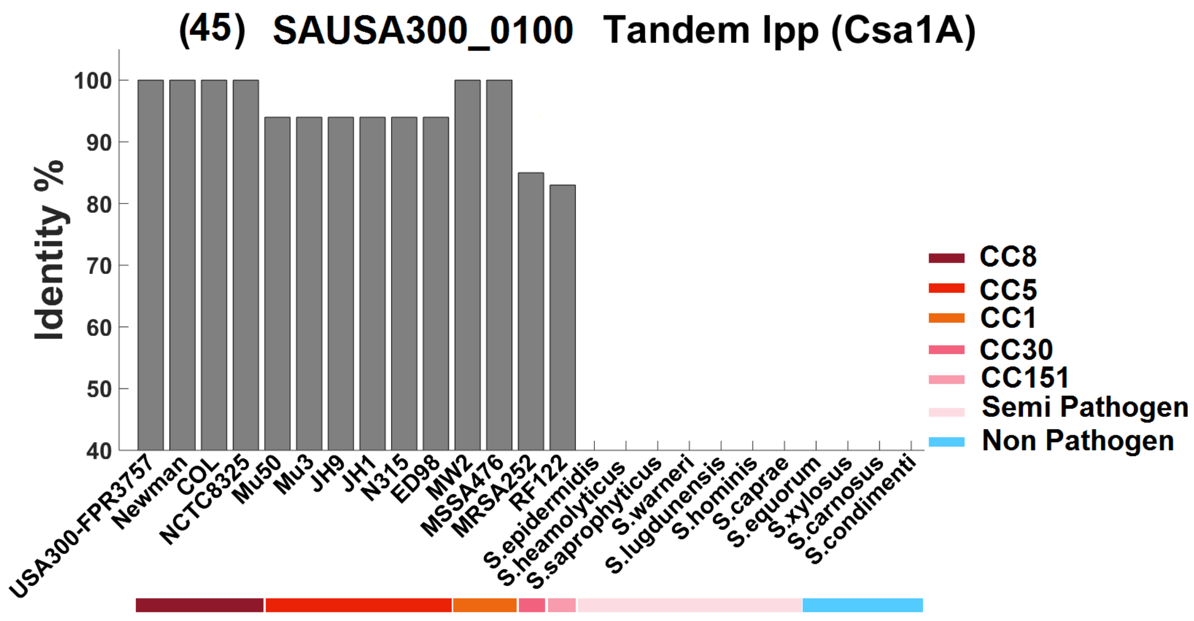
(45) SAUSA300_0100 Tandem lpp (Csa1A)
Identity %
100
90
80
70
60
50
40
USA300-FPR3757
Newman
COL
NCTC8325
Mu50
Mu3
JH9
JH1
N315
ED98
MW2
MSSA476
MRSA252
RF122
S.epidermidis
S.heamolyticus
S.saprophyticus
S.warneri
S.lugdunensis
S.hominis
S.caprae
S.equorum
S.xylosus
S.carnosus
S.condimenti
CC8
CC5
CC1
CC30
CC151
Semi Pathogen
Non Pathogen

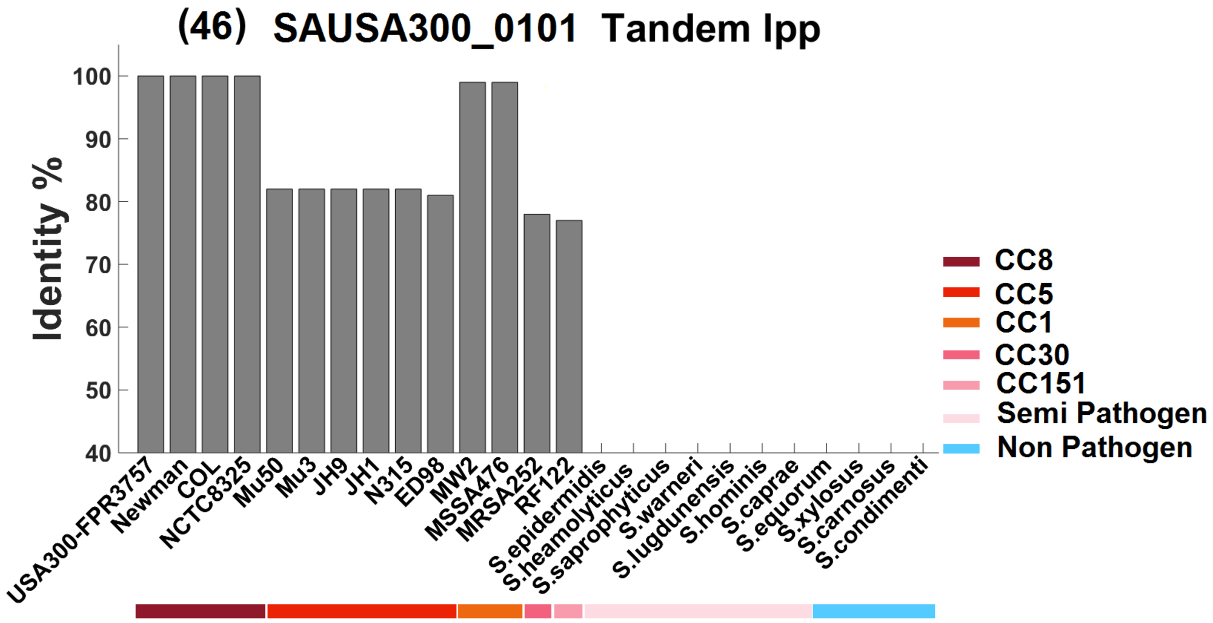
(46) SAUSA300_0101 Tandem lpp
Identity %
100
90
80
70
60
50
40
USA300-FPR3757
Newman
COL
NCTC8325
Mu50
Mu3
JH9
JH1
N315
ED98
MW2
MSSA476
MRSA252
RF122
S.epidermidis
S.heamolyticus
S.saprophyticus
S.warneri
S.lugdunensis
S.hominis
S.caprae
S.equorum
S.xylosus
S.carnosus
S.condimenti
CC8
CC5
CC1
CC30
CC151
Semi Pathogen
Non Pathogen

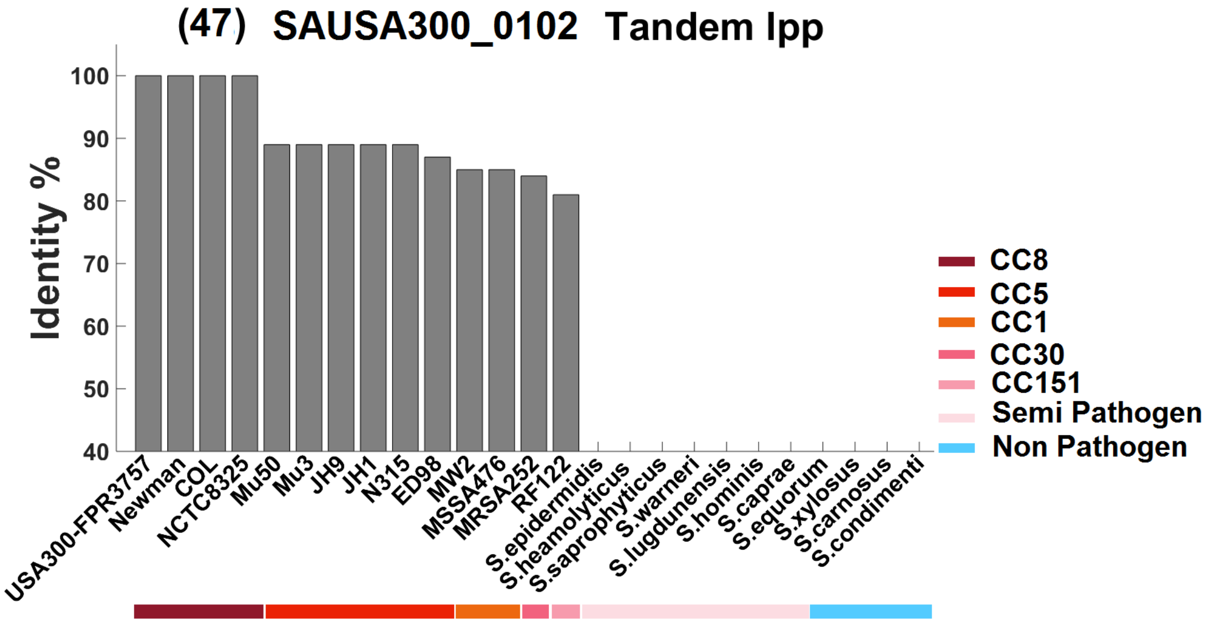
(47) SAUSA300_0102 Tandem lpp
Identity %
100
90
80
70
60
50
40
USA300-FPR3757
Newman
COL
NCTC8325
Mu50
Mu3
JH9
JH1
N315
ED98
MW2
MSSA476
MRSA252
RF122
S.epidermidis
S.heamolyticus
S.saprophyticus
S.warneri
S.lugdunensis
S.hominis
S.caprae
S.equorum
S.xylosus
S.carnosus
S.condimenti
CC8
CC5
CC1
CC30
CC151
Semi Pathogen
Non Pathogen

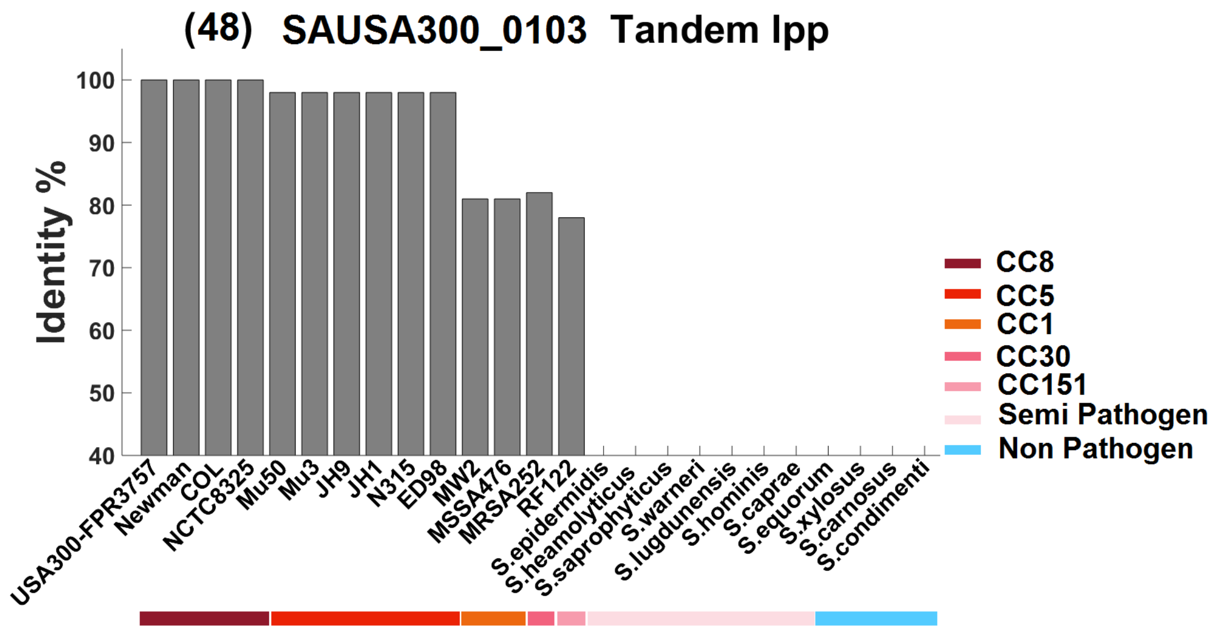
(48) SAUSA300_0103 Tandem lpp
Identity %
100
90
80
70
60
50
40
USA300-FPR3757
Newman
COL
NCTC8325
Mu50
Mu3
JH9
JH1
N315
ED98
MW2
MSSA476
MRSA252
RF122
S.epidermidis
S.heamolyticus
S.saprophyticus
S.warneri
S.lugdunensis
S.hominis
S.caprae
S.equorum
S.xylosus
S.carnosus
S.condimenti
CC8
CC5
CC1
CC30
CC151
Semi Pathogen
Non Pathogen

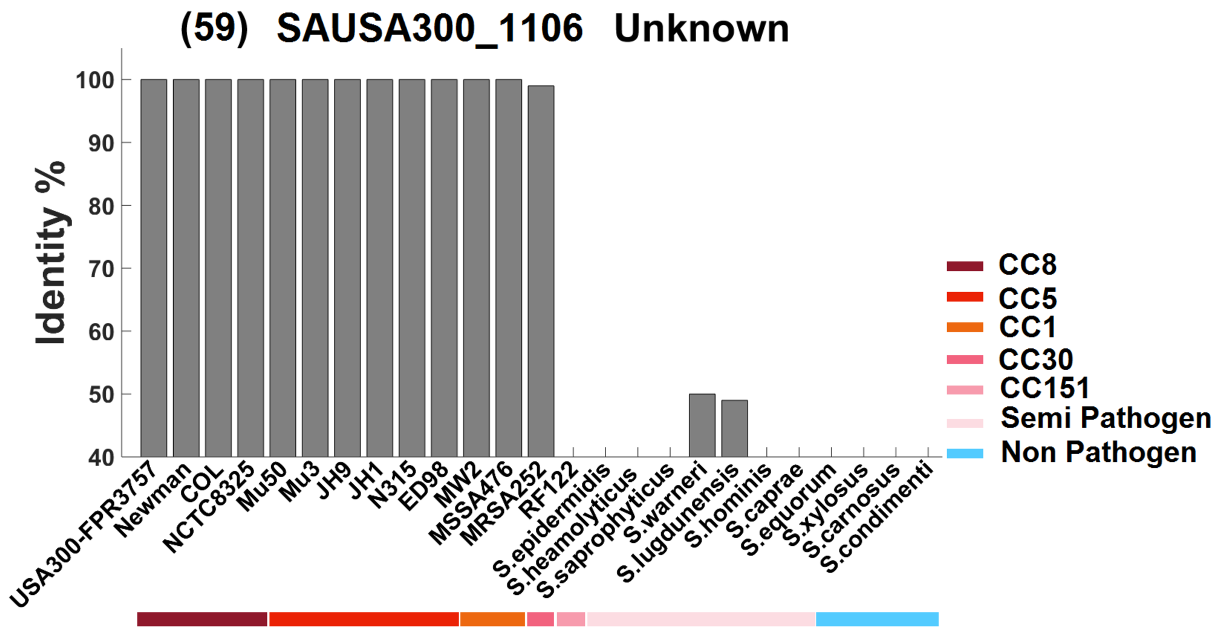
(59) SAUSA300_1106 Unknown
Identity %
100
90
80
70
60
50
40
USA300-FPR3757
Newman
COL
NCTC8325
Mu50
Mu3
JH9
JH1
N315
ED98
MW2
MSSA476
MRSA252
RF122
S.epidermidis
S.heamolyticus
S.saprophyticus
S.warneri
S.lugdunensis
S.hominis
S.caprae
S.equorum
S.xylosus
S.carnosus
S.condimenti
CC8
CC5
CC1
CC30
CC151
Semi Pathogen
Non Pathogen

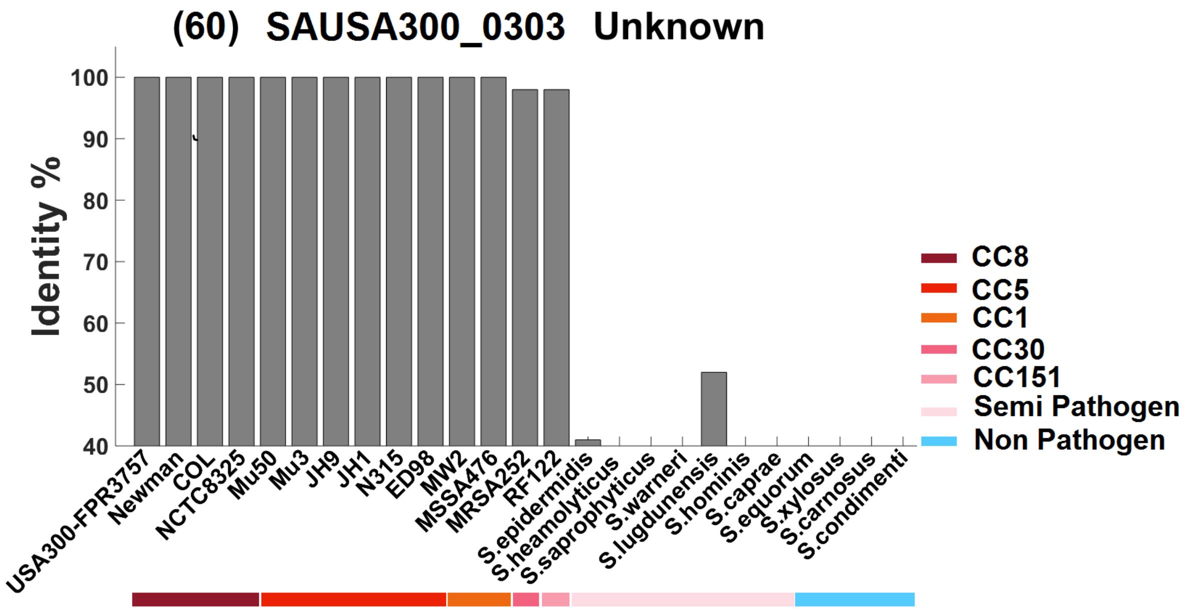

(60) SAUSA300_0303 Unknown
Identity %
100
90
80
70
60
50
40
USA300-FPR3757
Newman
COL
NCTC8325
Mu50
Mu3
JH9
JH1
N315
ED98
MW2
MSSA476
MRSA252
RF122
S.epidermidis
S.heamolyticus
S.saprophyticus
S.warneri
S.lugdunensis
S.hominis
S.caprae
S.equorum
S.xylosus
S.carnosus
S.condimenti
CC8
CC5
CC1
CC30
CC151
Semi Pathogen
Non Pathogen

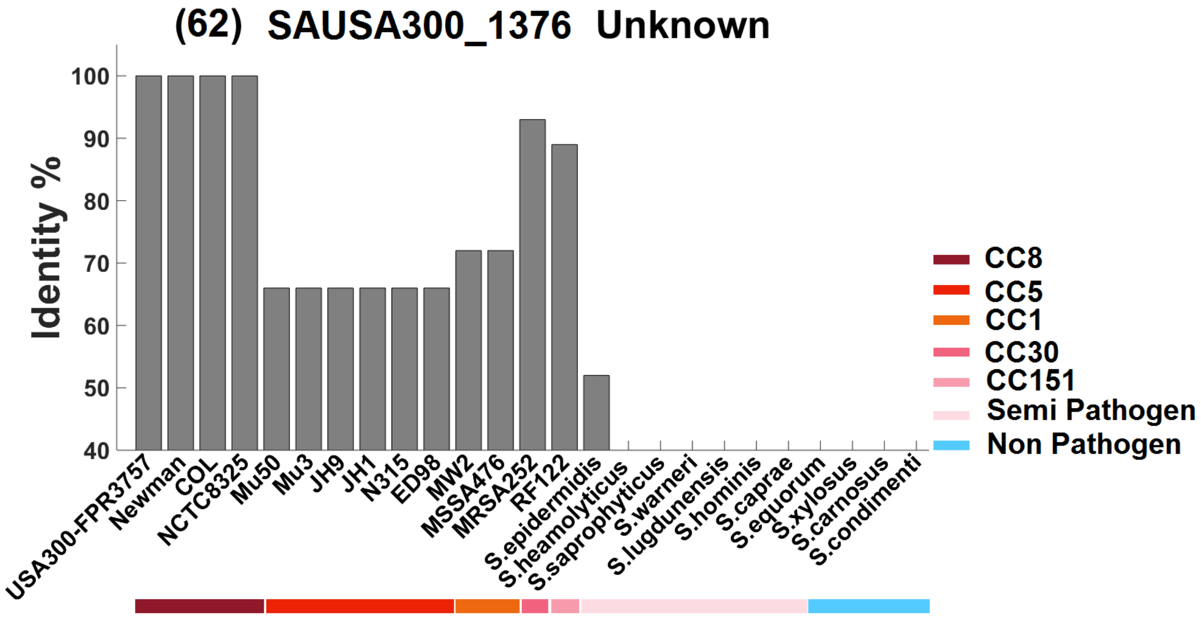

(62) SAUSA300_1376 Unknown
Identity %
100
90
80
70
60
50
40
USA300-FPR3757
Newman
COL
NCTC8325
Mu50
Mu3
JH9
JH1
N315
ED98
MW2
MSSA476
MRSA252
RF122
S.epidermidis
S.heamolyticus
S.saprophyticus
S.warneri
S.lugdunensis
S.hominis
S.caprae
S.equorum
S.xylosus
S.carnosus
S.condimenti
CC8
CC5
CC1
CC30
CC151
Semi Pathogen
Non Pathogen

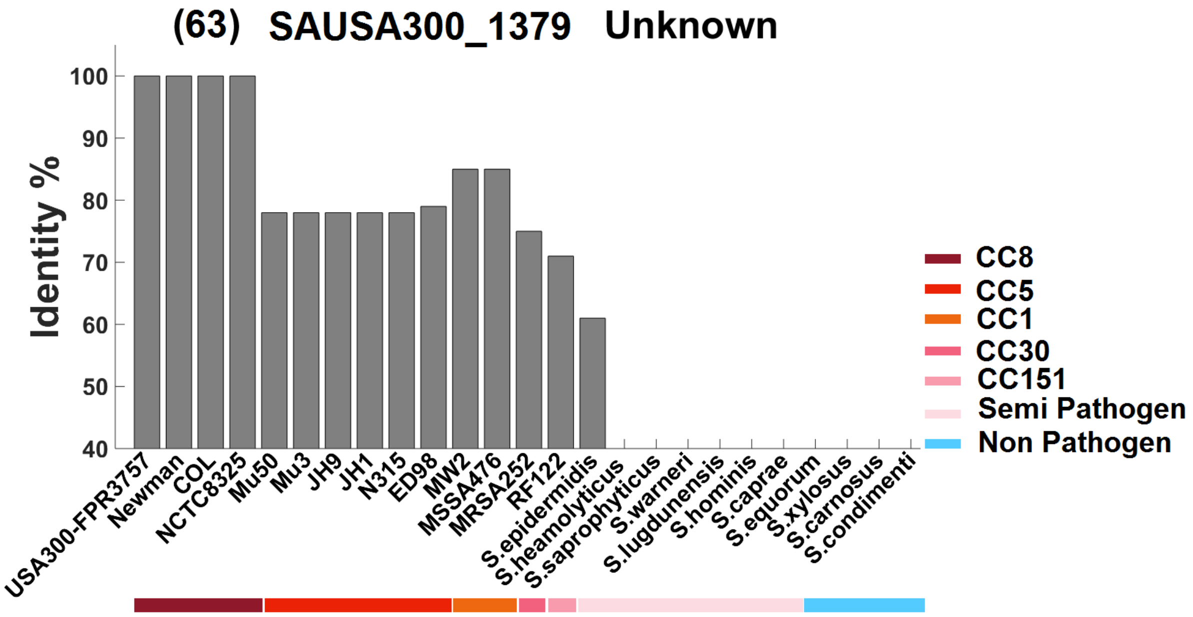
(63) SAUSA300_1379 Unknown
Identity %
100
90
80
70
60
50
40
USA300-FPR3757
Newman
COL
NCTC8325
Mu50
Mu3
JH9
JH1
N315
ED98
MW2
MSSA476
MRSA252
RF122
S.epidermidis
S.heamolyticus
S.saprophyticus
S.warneri
S.lugdunensis
S.hominis
S.caprae
S.equorum
S.xylosus
S.carnosus
S.condimenti
CC8
CC5
CC1
CC30
CC151
Semi Pathogen
Non Pathogen

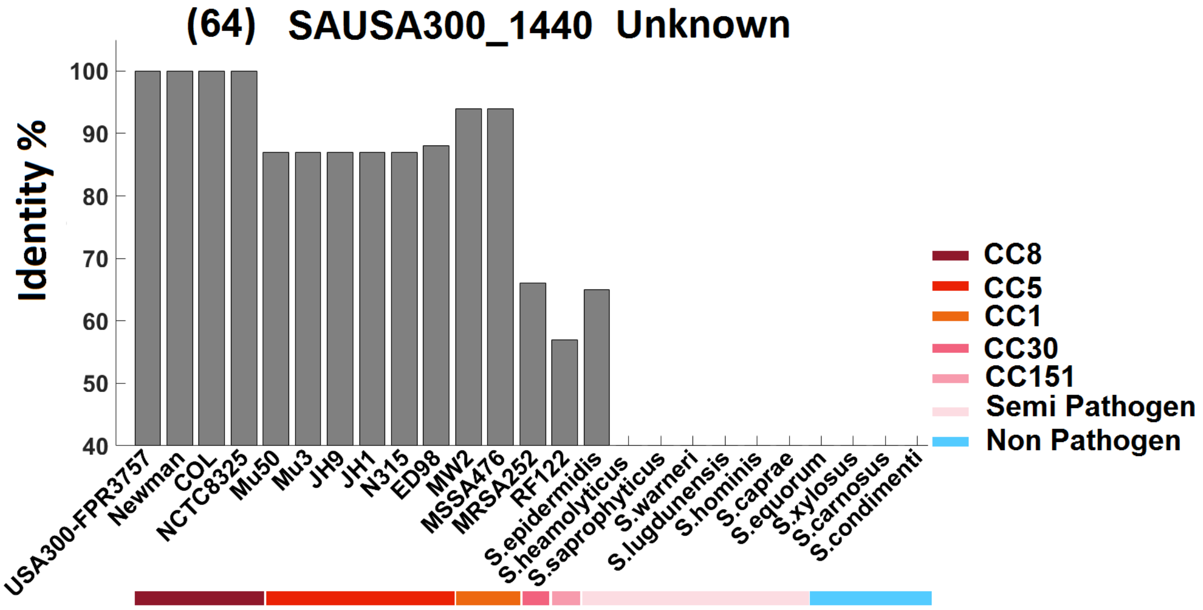
(64) SAUSA300_1440 Unknown
Identity %
100
90
80
70
60
50
40
USA300-FPR3757
Newman
COL
NCTC8325
Mu50
Mu3
JH9
JH1
N315
ED98
MW2
MSSA476
MRSA252
RF122
S.epidermidis
S.heamolyticus
S.saprophyticus
S.warneri
S.lugdunensis
S.hominis
S.caprae
S.equorum
S.xylosus
S.carnosus
S.condimenti
CC8
CC5
CC1
CC30
CC151
Semi Pathogen
Non Pathogen

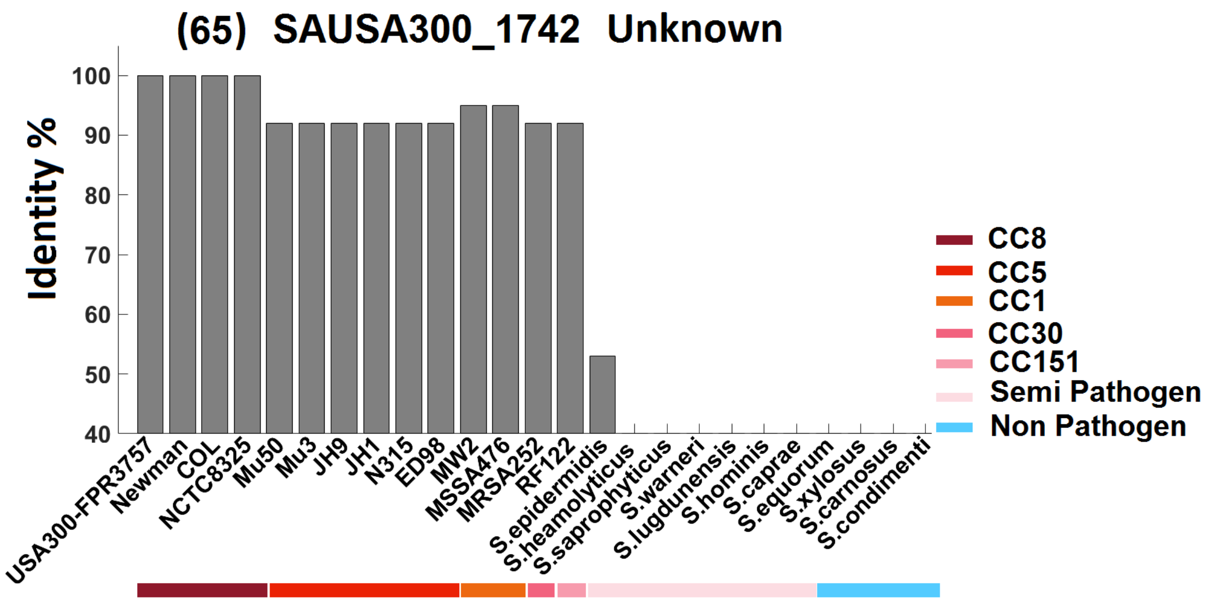

(65) SAUSA300_1742 Unknown
Identity %
100
90
80
70
60
50
40
USA300-FPR3757
Newman
COL
NCTC8325
Mu50
Mu3
JH9
JH1
N315
ED98
MW2
MSSA476
MRSA252
RF122
S.epidermidis
S.heamolyticus
S.saprophyticus
S.warneri
S.lugdunensis
S.hominis
S.caprae
S.equorum
S.xylosus
S.carnosus
S.condimenti
CC8
CC5
CC1
CC30
CC151
Semi Pathogen
Non Pathogen

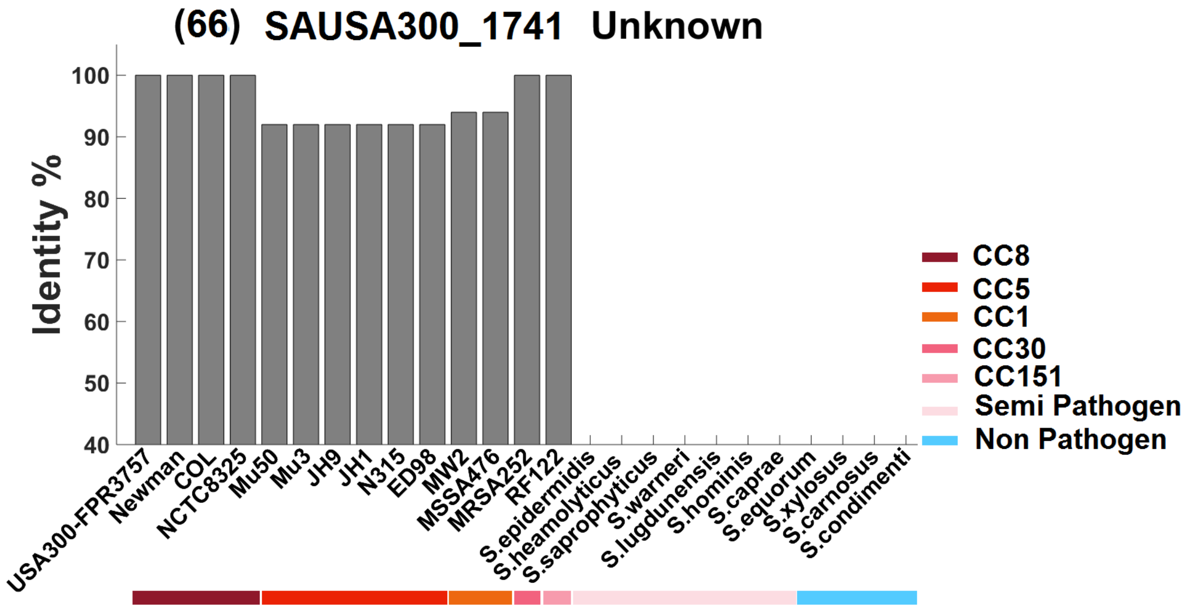

(66) SAUSA300_1741 Unknown
Identity %
100
90
80
70
60
50
40
USA300-FPR3757
Newman
COL
NCTC8325
Mu50
Mu3
JH9
JH1
N315
ED98
MW2
MSSA476
MRSA252
RF122
S.epidermidis
S.heamolyticus
S.saprophyticus
S.warneri
S.lugdunensis
S.hominis
S.caprae
S.equorum
S.xylosus
S.carnosus
S.condimenti
CC8
CC5
CC1
CC30
CC151
Semi Pathogen
Non Pathogen

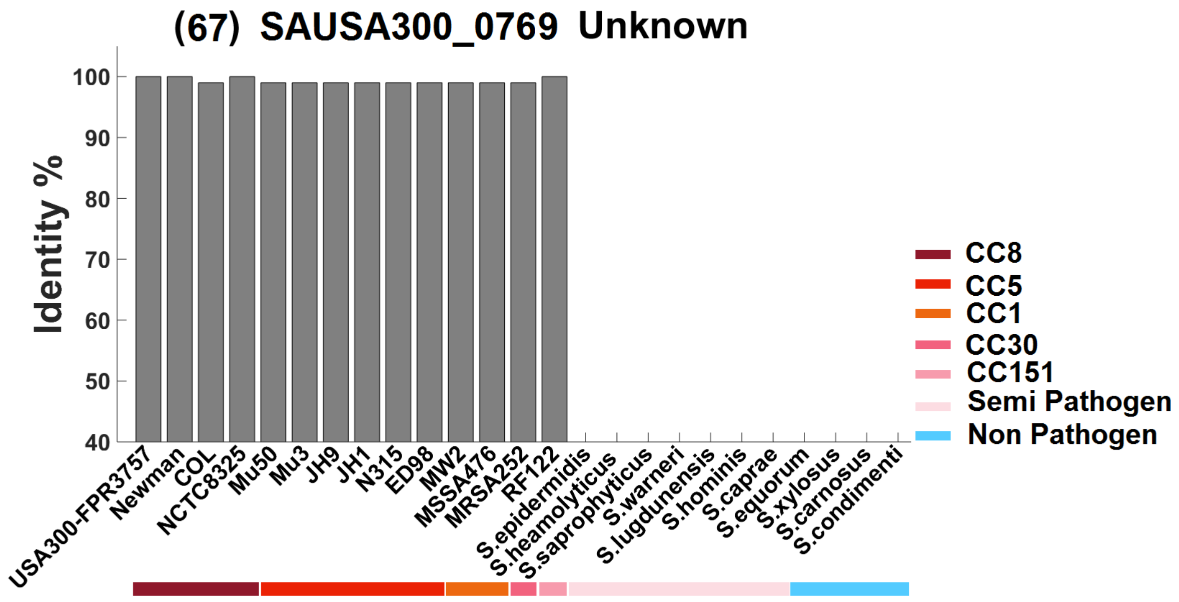

(67) SAUSA300_0769 Unknown
Identity %
100
90
80
70
60
50
40
USA300-FPR3757
Newman
COL
NCTC8325
Mu50
Mu3
JH9
JH1
N315
ED98
MW2
MSSA476
MRSA252
RF122
S.epidermidis
S.heamolyticus
S.saprophyticus
S.warneri
S.lugdunensis
S.hominis
S.caprae
S.equorum
S.xylosus
S.carnosus
S.condimenti
CC8
CC5
CC1
CC30
CC151
Semi Pathogen
Non Pathogen

(D)

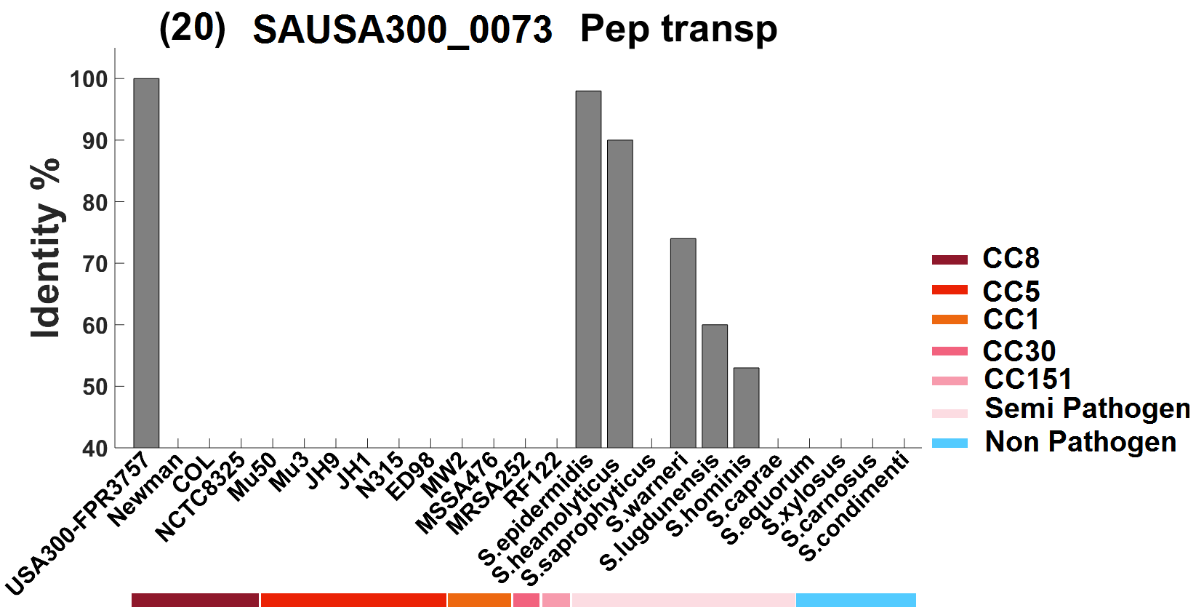
(20) SAUSA300_0073 Pep transp
Identity %
100
90
80
70
60
50
40
USA300-FPR3757
Newman
COL
NCTC8325
Mu50
Mu3
JH9
JH1
N315
ED98
MW2
MSSA476
MRSA252
RF122
S.epidermidis
S.heamolyticus
S.saprophyticus
S.warneri
S.lugdunensis
S.hominis
S.caprae
S.equorum
S.xylosus
S.carnosus
S.condimenti
CC8
CC5
CC1
CC30
CC151
Semi Pathogen
Non Pathogen

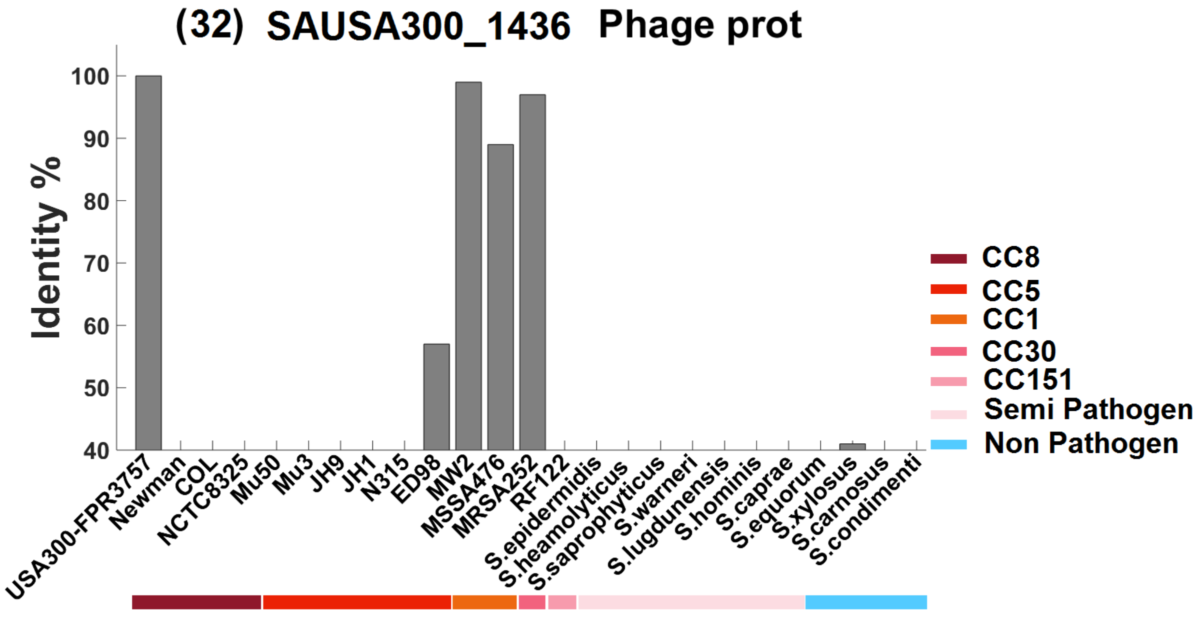
(32) SAUSA300_1436 Phage prot
Identity %
100
90
80
70
60
50
40
USA300-FPR3757
Newman
COL
NCTC8325
Mu50
Mu3
JH9
JH1
N315
ED98
MW2
MSSA476
MRSA252
RF122
S.epidermidis
S.heamolyticus
S.saprophyticus
S.warneri
S.lugdunensis
S.hominis
S.caprae
S.equorum
S.xylosus
S.carnosus
S.condimenti
CC8
CC5
CC1
CC30
CC151
Semi Pathogen
Non Pathogen

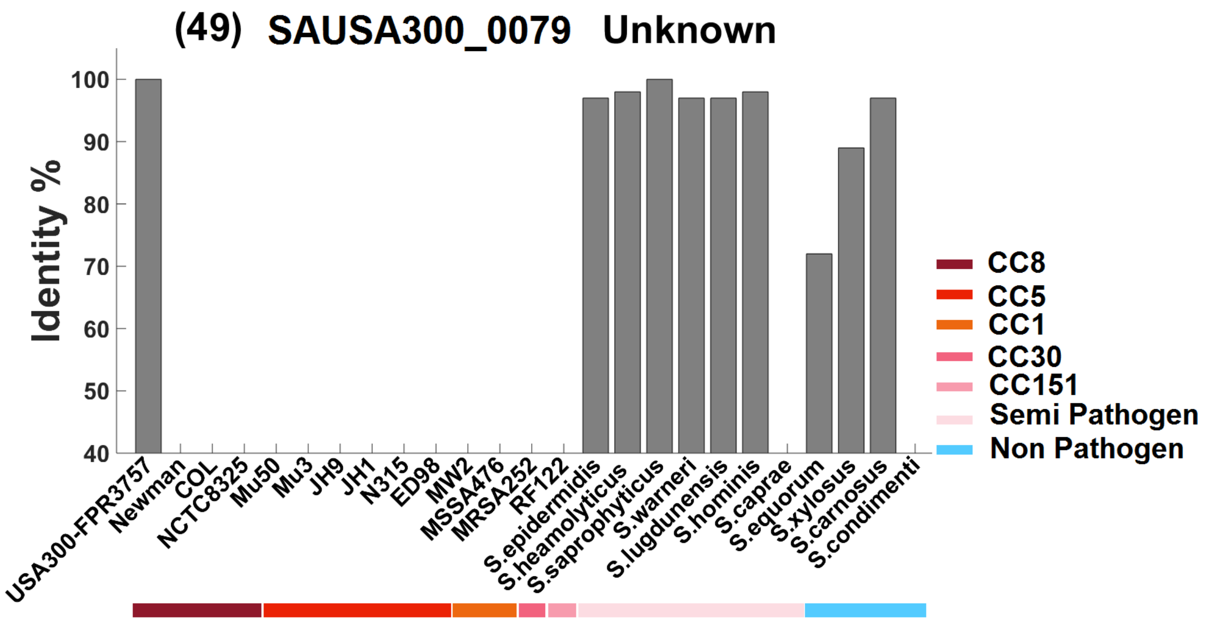

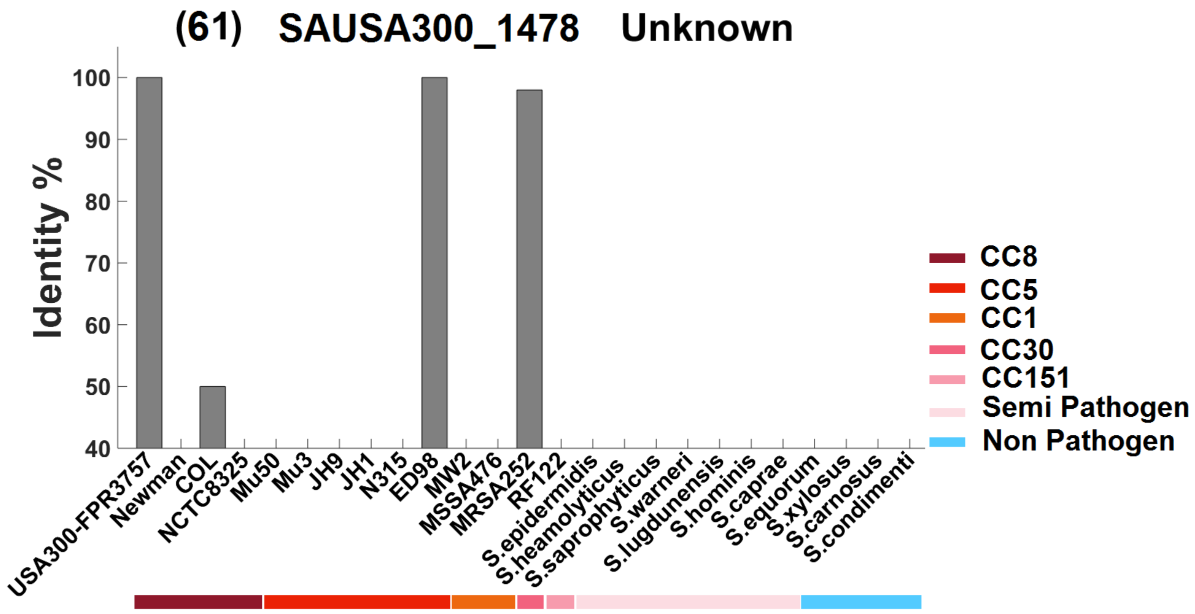

Supplementary Figure 1. Comparative analyses of amino acid sequences of corresponding Lpp in different *S. aureus* strains and staphylococcal species. (A) Group of highly conserved Lpp in Gram-positive, Gram-negative bacteria; **(B)** Group of Lpp in *S. aureus* occurring mainly in the genus *Staphylococcus*; **(C)** Group of Lpp specific only for *S.*

aureus, some coagulase-negative species and few other genera ; **(D)** Group of Lpp strain specific for *S. aureus* USA300

The cut-off for amino acid sequence identity was 40%. Color gradient ranged from dark red (highly pathogenic) to blue (non pathogenic). CC, clonal complexes of *S. aureus* lineages. Numbering and function correlates with the numbering and proposed function listed in Tab. 1.
